# On the relation between the cause‐specific hazard and the subdistribution rate for competing risks data: The Fine–Gray model revisited

**DOI:** 10.1002/bimj.201800274

**Published:** 2020-03-04

**Authors:** Hein Putter, Martin Schumacher, Hans C. van Houwelingen

**Affiliations:** ^1^ Department of Biomedical Data Sciences Leiden University Medical Center Leiden The Netherlands; ^2^ Institute for Medical Biometry and Statistics Faculty of Medicine and Medical Center University of Freiburg Freiburg im Breisgau Germany

**Keywords:** cause‐specific hazard, competing risks, cumulative incidence, proportional hazards, subdistribution hazard

## Abstract

The Fine–Gray proportional subdistribution hazards model has been puzzling many people since its introduction. The main reason for the uneasy feeling is that the approach considers individuals still at risk for an event of cause 1 after they fell victim to the competing risk of cause 2. The subdistribution hazard and the extended risk sets, where subjects who failed of the competing risk remain in the risk set, are generally perceived as unnatural . One could say it is somewhat of a riddle why the Fine–Gray approach yields valid inference. To take away these uneasy feelings, we explore the link between the Fine–Gray and cause‐specific approaches in more detail. We introduce the reduction factor as representing the proportion of subjects in the Fine–Gray risk set that has not yet experienced a competing event. In the presence of covariates, the dependence of the reduction factor on a covariate gives information on how the effect of the covariate on the cause‐specific hazard and the subdistribution hazard relate. We discuss estimation and modeling of the reduction factor, and show how they can be used in various ways to estimate cumulative incidences, given the covariates. Methods are illustrated on data of the European Society for Blood and Marrow Transplantation.

## 1 INTRODUCTION

Competing risks are common in medical survival data, when patients can experience one of a number of mutually exclusive competing events. Examples are death of different causes, or recurrence of disease, when death is a competing risk (Prentice et al., 1978; Putter, Fiocco, & Geskus, 2007; Beyersmann, Allignol, & Schumacher, 2011; Andersen, Geskus, de Witte, & Putter, 2012; Geskus, 2015). When interest is in the effect of a covariate on an event of interest in the presence of competing risks, two main approaches are in use, both based on proportional hazards. The first approach imposes a proportional hazards assumption on the cause‐specific hazards. After estimating the regression coefficients and baseline hazards, probability calculations can be made to quantify the effect of the covariate on the probability scale, the cumulative incidence (Andersen et al., 2002). The situation can occur that, with an estimated positive regression coefficient for the cause‐specific hazard of interest, higher values of the covariate do not coincide with higher probabilities of the event of interest. The reason for this, perhaps unexpected, behaviour, is that the probability of occurrence of the event of interest depends on *both* cause‐specific hazards. Motivated by this complication, Fine and Gray (1999) developed their proportional subdistribution hazards model, nowadays commonly known as the Fine–Gray model.

The Fine–Gray model has been puzzling many people since its introduction. The main reason for the uneasy feeling is that the approach considers individuals still at risk for an event of cause 1 after they fell victim to cause 2 (the competing risk). The “explanation” is that the subdistribution is only interested in risk 1 and does not want any information about the occurrence of other competing events. The uneasiness is about not using the information that an individual is not at risk any more for other events if one particular event has occurred. One might say that in the extended risk sets of the Fine–Gray partial likelihood there is heterogeneity among the members of the risk set, one subgroup still can get the event, while the other subgroup cannot experience any further events. One might wonder whether this heterogeneity might introduce bias or lead to loss of efficiency. The subdistribution “hazard” and the extended risks sets of the Fine–Gray approach are perceived by most as unnatural concepts; the riddle is why the Fine–Gray approach works and why the partial likelihood based on the extended risk sets, and subsequent inference based on it is valid.

A related issue is that the cumulative incidence function that is directly estimated in the Fine–Gray approach cannot be used dynamically. In conditional (landmark) models one wants to condition on still being “alive” and not on not having experienced the event of interest. That explains why Cortese, Gerds, and Andersen (2013) have to compute separate Fine–Gray models for each landmark point they consider.

To take away these uneasy feelings our aim is to explore the link between the Fine–Gray and cause‐specific approaches in more detail. In Section 2, we study the relation between cause‐specific and subdistribution hazards, and we introduce the *reduction factor*. Section 3 discusses estimation and modeling of the reduction factor. Section 4 shows how the reduction factor can play a role in estimating cumulative incidence functions. Section 5 illustrates estimation of the reduction factor and its use in modeling cumulative incidence functions in a data set of CML patients. The paper ends with a discussion in Section 6.

## 2 ON THE RELATION BETWEEN THE CAUSE‐SPECIFIC AND SUBDISTRIBUTION HAZARD

### 2.1 Multistate description of the problem

To simplify the discussion we consider only two competing risks, risk 1 is the risk of interest and risk 2 is the union of all other competing risks. We can use a multistate model with the following three states to represent the competing risks problem:

State 0 (S0) The initial state at t=0;
State 1 (S1) The absorbing state of risk 1;
State 2 (S2) The absorbing state of risk 2.

**Figure 1:**
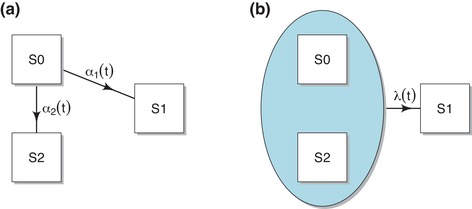
Graphical presentation of the (a) cause‐specific (multistate) approach and the (b) Fine–Gray approach

See Figure 1a for a graphical representation.

Let Z(t) denote the multistate model process. The transition rates from state 0 to states 1 and 2 are the *cause‐specific* hazard rates, denoted by $\alpha_{1}\left(t\right)=\lim_{\Delta t↓0}P\left(Z\left(t+\Delta t\right)=1\;|\;Z\left(t\right)=0\right)/\Delta t$ and $\alpha_{2}\left(t\right)=\lim_{\Delta t↓0}P\left(Z\left(t+\Delta t\right)=2\;|\;Z\left(t\right)=0\right)/\Delta t$. The state occupation probabilities are denoted by S(t)=P(Z(t)=0), $F_{1}\left(t\right)=P\left(Z\left(t\right)=1\right)$ and $F_{2}\left(t\right)=P\left(Z\left(t\right)=2\right)$; S(t) is the probability of being event‐free up to time *t*, and $F_{1}\left(t\right)$ and $F_{2}\left(t\right)$ are the cause‐specific *cumulative incidences* of cause 1 and 2, respectively. Assuming that the $\alpha_{k}$'s are continuous, the relation between $F_{k}\left(t\right)$ and the cause‐specific hazards are given by

(1) $$\begin{array}{ccc} S(t) & = & \exp[-\left\{A_{1}\left(t\right)+A_{2}\left(t\right)\right\}],\\ F_{k}\left(t\right) & = & \int_{0}^{t}\alpha_{k}\left(s\right)\exp\left[-\left\{A_{1}\left(s\right)+A_{2}\left(s\right)\right\}\right]ds,\;k=1,2, \end{array}$$

where $A_{k}\left(t\right)=\int_{0}^{t}\alpha_{k}\left(s\right)ds$ is the cumulative cause‐specific hazard.

To explain the Fine–Gray approach for risk 1 we define ${\mathrm{State}}_{\mathrm{not}\;1}=\bar{\mathrm{State}\;1}=\mathrm{State}0\cup \mathrm{State}2$, the complement of State 1. The Fine–Gray approach is based on directly modeling λ(t), the rate of moving from ${\mathrm{State}}_{\mathrm{not}\;1}$ to State 1. See Figure 1b for a graphical presentation.

The rate λ(t) is referred to as the subdistribution hazard. That term might cause confusion with the cause‐specific hazard and it can be considered the hazard only of something that violates the three principles of Andersen and Keiding (2012), but we will the term nevertheless because it is well‐established in the literature. It is given by

$$\lambda \left(t\right)=-\frac{d\log\{1-F_{1}\left(t\right)\}}{dt}.$$

In principle there is also a subdistribution hazard of the competing risk, cause 2, but since the Fine–Gray model focuses on the cause of interest, cause 1, and *F*_1_ depends only on one subdistribution hazard, we omit the subscript 1 from the notation of λ(t). The advantage of the approach is that the cumulative incidence function $F_{1}\left(t\right)$ can be derived directly from λ(t), as $F_{1}\left(t\right)=1-\exp\left(-\int_{0}^{t}\lambda \left(s\right)ds\right)$. The subdistribution hazard λ(t) can be estimated by partial likelihood using ${\mathrm{State}}_{\mathrm{not}\;1}$ as the risk set. As stated in the introduction the uneasy elements are that the information about the occurrence of risk 2 is not used and that the risk set contains individuals that are only artificially at risk, because they already experienced the competing risk.

The link between λ(t) and $\alpha_{1}\left(t\right)$ is given by

(2) $$\lambda \left(t\right)=r\left(t\right)\alpha_{1}\left(t\right),\mathrm{with}r\left(t\right)=\frac{P(Z(t)=0)}{P(Z(t)=0)+P(Z(t)=2)},$$

see also Latouche, Boisson, Chevret, and Porcher (2007) and Beyersmann and Scheike (2013), among others.

### 2.2 The reduction factor

The identity (2) is crucial for understanding the relation between the $\alpha_{k}$'s and λ. We call r(t) the *reduction factor*. It represents the proportion of subjects in the extended risk set that has not yet experienced a competing event, and can be expressed as

(3) $$r\left(t\right)=\frac{S(t)}{1-F_{1}\left(t\right)}.$$

Observe that r(0)=1, 0≤r(t)≤1, and that r(t) is decreasing. An explanation of the fact that r(t) is decreasing is that P(T>t) decreases and $1-F_{1}\left(t\right)-P\left(T>t\right)$ increases over time, as it is the fraction that experienced the competing event before time *t*. Mathematically, it follows from the fact that $\log\left\{r\left(t\right)\right\}=-\left\{A_{1}\left(t\right)+A_{2}\left(t\right)\right\}-\log\left\{1-F_{1}\left(t\right)\right\}$, which yields

(4) $$\begin{array}{ccc} -\frac{d\log\{r(t)\}}{dt} & = & \alpha_{1}\left(t\right)+\alpha_{2}\left(t\right)-\frac{\alpha_{1}\left(t\right)\exp\left[-\left\{A_{1}\left(t\right)+A_{2}\left(t\right)\right\}\right]}{1-F_{1}\left(t\right)}\\ & = & \alpha_{1}\left(t\right)\left\{1-r\left(t\right)\right\}+\alpha_{2}\left(t\right). \end{array}$$

Note that the term $\frac{\alpha_{1}\left(t\right)\exp\left[-\left\{A_{1}\left(t\right)+A_{2}\left(t\right)\right\}\right]}{1-F_{1}\left(t\right)}$, coming from $\frac{d\log\{1-F_{1}\left(t\right)\}}{dt}$, is the subdistribution hazard λ(t). Notice also that r(0)=1 implies that the subdistribution hazard and the cause‐specific hazard are identical at t=0 and will start diverging later on. The negative derivative of log{r(t)} at t=0 equals α_2_(0); the larger the competing cause‐specific hazard, the larger the initial exponential decrease of r(t). In some sense r(t) initially behaves like a survival function with cumulative hazard rate $\alpha_{2}\left(t\right)$.

**Figure 2:**
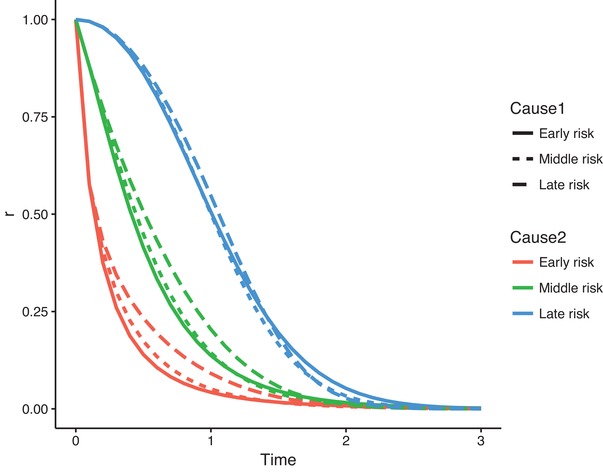
The reduction factor r(t) for different choices of Weibull hazards

Figure 2 illustrates the behavior of r(t) for a number of choices of cause‐specific hazards from the Weibull family, with hazard rate $h\left(t;a,b\right)=abt^{b-1}$, for a,b>0. We choose three values of the shape parameter *b*, namely b=0.5 (a decreasing hazard), b=1 (constant hazard), and b=2 (increasing hazard) and choose the corresponding rate parameters *a* in such a way that S(2.5;a,b)=0.05, with $S\left(t;a,b\right)=\exp(-\int_{0}^{t}h\left(s;a,b\right)ds)$ the survival function corresponding to h(t;a,b). This leads to the values 1.895, 1.198, and 0.479, for *a*, when b=0.5,1,2, respectively. We consider a competing risks situation with two causes, where the cause‐specific hazards of cause 1 and 2 are both chosen from these three Weibull hazards, with b=0.5,1,2 and corresponding *a* values. We denote the hazard with b=0.5 as “Early risk,” with b=1 as “Middle risk,” and with b=2 as “Late risk.” The reduction factor r(t) was obtained by numerically solving the differential equation (4). The general behavior seen from Figure 2 is that r(t) decreases more rapidly when the competing cause‐specific hazard has an earlier risk. The effect of the shape of the cause‐specific hazards of the cause of interest is less pronounced.

Of course, the particular figure is highly dependent on the choice of parameters. The Supporting Information contains R code to reproduce the figure, and to change the settings of the parameters.

Remark 1 Equation (4) not only gives a formula for r(t) in terms of $\alpha_{1}\left(t\right)$ and $\alpha_{2}\left(t\right)$, but also of $\alpha_{2}\left(t\right)$ in terms of r(t) and $\alpha_{1}\left(t\right)$, and even of $\alpha_{1}\left(t\right)$ in terms of r(t) and $\alpha_{2}\left(t\right)$. The usefulness of these relations will be discussed later.

### 2.3 The reduction factor and covariates

The reduction factor describes the relation between the subdistribution hazard and cause‐specific hazards. This implies that the dependence of the reduction factor on a covariate *x* gives information on how the effect of *x* on the cause‐specific and subdistribution hazards relate. To illustrate this point, suppose that the cause‐specific hazard is given by

$$\alpha_{1}\left(t\;|\;x\right)=\alpha_{10}\left(t\right)\exp\left\{\beta_{\mathrm{CS}}\left(t\right)x\right\},$$

and the subdistribution hazard by

$$\lambda \left(t\;|\;x\right)=\lambda_{0}\left(t\right)\exp\left\{\beta_{\mathrm{FG}}\left(t\right)x\right\}.$$

Then the reduction factor has a similar form

$$r\left(t\;|\;x\right)=r_{0}\left(t\right)\exp\left\{\beta_{\mathrm{RF}}\left(t\right)x\right\},$$

with $r_{0}\left(t\right)=\lambda_{0}\left(t\right)/\alpha_{10}\left(t\right)$, and

(5) $$\beta_{\mathrm{RF}}\left(t\right)=\beta_{\mathrm{FG}}\left(t\right)-\beta_{\mathrm{CS}}\left(t\right).$$

Note that valid reduction factors have r(0)=1, so we must have $r_{0}\left(0\right)=1$ and $\beta_{\mathrm{RF}}\left(0\right)=0$.

Equation (5) quantifies how the regression coefficients in a Fine–Gray model and in a cause‐specific hazards model differ. In particular, if $\beta_{\mathrm{CS}}$ and $\beta_{\mathrm{RF}}$ are time‐fixed, we get the simple relation $\beta_{\mathrm{RF}}=\beta_{\mathrm{FG}}-\beta_{\mathrm{CS}}$. For this to really hold, in view of the above we must have $\beta_{\mathrm{RF}}=0$. Cause‐specific and subdistribution hazards can typically not both fulfill the proportional hazards assumption at the same time, unless $\beta_{\mathrm{FG}}=\beta_{\mathrm{CS}}$ (Grambauer, Schumacher, & Beyersmann, 2010).

**Figure 3:**
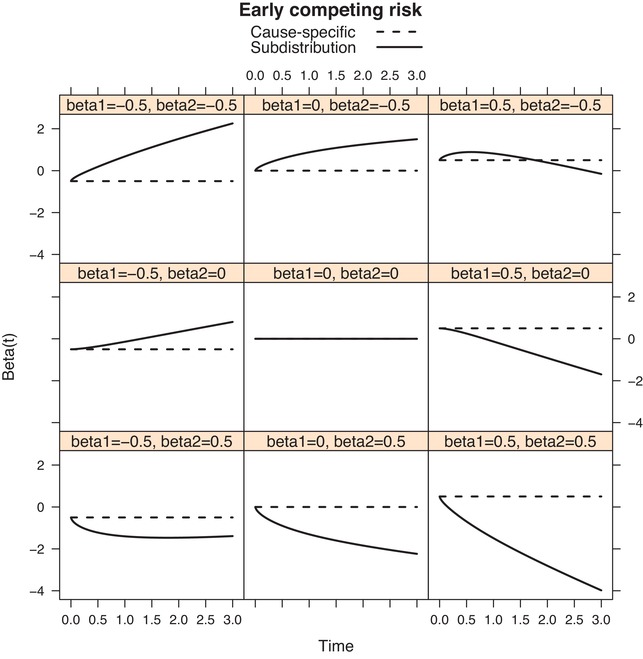
“Early” competing risk; the regression coefficients $\beta_{\mathrm{CS}}\left(t\right)$ (dashed lines) and $\beta_{\mathrm{FG}}\left(t\right)$ (solid lines) for the cause‐specific and subdistribution hazards respectively, for different choices of β_1_ and β_2_

**Figure 4:**
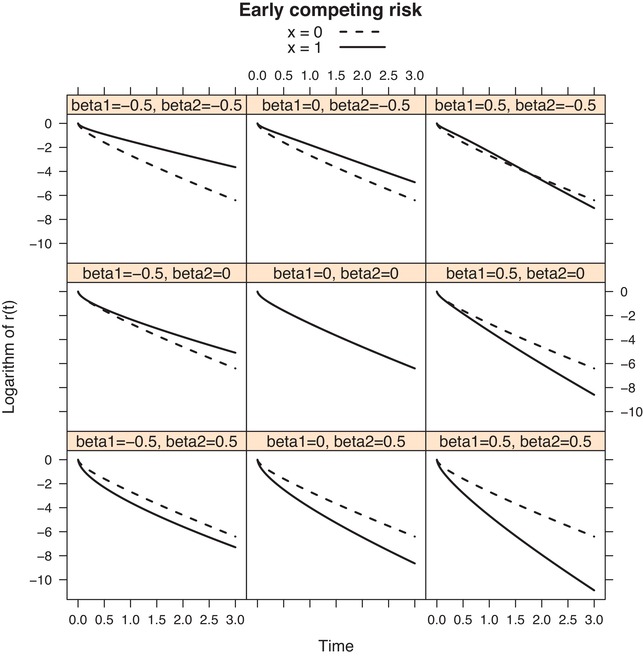
“Early” competing risk; the log reduction factor r(t|x) for different choices of β_1_ and β_2_

Figures 3 and 4 show the behaviour of $\beta_{\mathrm{FG}}\left(t\right)$ and of the logarithm of r(t|x) in the case of proportional cause‐specific hazards models, for the Weibull hazards we used in Figure 2. The three choices of Weibull hazards (early, middle, and late risk) were used as baseline cause‐specific hazards for the competing risk, while for cause 1 the middle risk (constant hazard) was selected as baseline hazard. A single binary covariate *x* with mean p=0.5 was used with a proportional effect for both cause‐specific hazards; with regression coefficients β_1_ and $\beta_{2}=-0.5$, 0, 0.5, for cause 1 and 2, respectively. The solid curves in Figure 3 show the behaviour of $\beta_{\mathrm{FG}}\left(t\right)$ implied by each of the nine different choices of β_1_ and β_2_ (referred to as beta1 and beta2 in the figure), for the case where the competing risk is “Early risk.” The dotted lines represent $\beta_{\mathrm{CS}}\left(t\right)$, which is time‐constant and trivially equals $\beta_{1}=-0.5$, 0 and 0.5 in the left, middle, and right column, respectively. Note that $\beta_{\mathrm{FG}}\left(0\right)=\beta_{1}$, and that $\beta_{\mathrm{FG}}\left(t\right)$ initially increases if $\beta_{2}<0$ and initially decreases if $\beta_{2}>0$. This can be explained from the fact that $-\frac{d\log\{r(t)\}}{dt}{|}_{t=0}=\alpha_{2}\left(0\right)$, which implies that here $\beta_{\mathrm{FG}}^{\prime }\left(0\right)=\alpha_{20}\left(0\right)\left\{\exp\left(\beta_{2}\right)-1\right\}$. Note also that $\beta_{1}=\beta_{2}=0$ is the only case when $\beta_{\mathrm{FG}}\left(t\right)$ is time‐constant (and equal to 0). Figure 4 shows the resulting r(t|x) for x=0 and x=1 on a logarithmic scale, for each of the possibilities of β_1_ and β_2_, also for the case where the competing risk is “Early risk.” It is seen that the reduction curves behave like survival curves (they start in 1 and decrease monotonically). The main reason for showing the logarithmic scale is that the difference between the logarithms of r(t|x=1) and r(t|x=0) equals $\beta_{\mathrm{FG}}\left(t\right)$. Similar figures for “Middle risk” and “Late risk” can be obtained by running the code available in the Supporting Information. For later risk, both $\beta_{\mathrm{FG}}\left(t\right)$ and r(t) start to diverge later.

Equation (2) allows estimating the subdistribution hazard without using the partial likelihood on the extended risk set, the main cause of concern about the Fine–Gray approach. It needs a model for the reduction factor r(t) which is not a transition rate, but a ratio of the prevalent cumulative incidence functions $r\left(t\right)=\left\{1-F_{1}\left(t\right)-F_{2}\left(t\right)\right\}/\left\{1-F_{1}\left(t\right)\right\}$ that can be estimated through binomial models or empirical proportions. The drawback is that the resulting model for the subdistribution hazard will be far from simple. A simple model can be obtained by a little trick. Let the desired simple model for the subdistribution hazard be $\lambda \left(t\;|\;x\right)=\lambda_{0}\left(t\right)\exp\left(\beta_{\mathrm{FG}}x\right)$. From Equation (2), we can write

(6) $$\alpha_{1}\left(t\;|\;x\right)=\lambda \left(t\;|\;x\right)/r\left(t\;|\;x\right)=\lambda_{0}\left(t\right)\exp\left\{\beta_{\mathrm{FG}}x-\log r\left(t\;|\;x\right)\right\}.$$

Equation (6) implies that, once r(t|x) has been estimated, $\beta_{\mathrm{FG}}$ can be estimated by fitting a model on the *cause‐specific hazard*, using the negative of log{r(t|x)} as offset. Partial likelihood in the “proper” risk sets is used, although some programming is needed to handle the offset term. Note that using −log{r(t|x)} as offset is different from inverse probability weighting, although it has a similar flavour. Note also that, although the cause‐specific hazards model on the right hand side of Equation (6) looks like a proportional cause‐specific hazards model because of the presence of $\beta_{\mathrm{FG}}x$ in the exponent, it is not because the offset term, although not associated with a parameter, is time‐dependent and depends on *x*. In the next section we show the remarkable fact that when *x* is a categorical covariate and the negative logarithm of the non‐parametric estimate of the reduction factor is used as offset in the partial likelihood for cause‐specific hazards, the resulting estimate equals the Fine–Gray estimate. In order to show this, we consider estimation and modeling of the reduction factor in the next section.

## 3 HAZARD REGRESSION MODELS AND THE REDUCTION FACTOR

### 3.1 Notation, risk sets, and partial likelihoods

We assume that there are no ties in the data and initially we also assume there is no censoring before the observation horizon, in other words we consider estimation before that horizon. Censoring will be discussed at the end of this section. For simplicity, we assume there are two competing risks, and we suppose again that cause 1 is the event of primary interest.

The data are given by realizations $\left(t_{i},d_{i},x_{i}\right)$ of $\left(T_{i},D_{i},X_{i}\right)$, for i=1,…,n, with $t_{i}$ the event or censoring time, and $d_{i}$ the type of event. In the case of right censoring, considered later, we have $d_{i}=0$. Define $D_{1}$ as the set of all cause 1 event time points, that is, the distinct $t_{i}$ for which the corresponding $d_{i}$ equals 1. Denote the “usual” risk set at time *t*, used for inference on the cause‐specific hazards, by $R_{\mathrm{CS}}\left(t\right)=\left\{i;T_{i}\geq t\right\}$, and the extended Fine–Gray risk set by $R_{\mathrm{FG}}\left(t\right)=\left\{i;T_{i}\geq t\;\text{or}\;D_{i}\neq 1\right\}$. In the absence of censoring, the Fine–Gray risk set can be obtained by replacing any $\left(t_{i},d_{i}=2,x_{i}\right)$ by $\left(t_{i}=\infty ,d_{i}=0,x_{i}\right)$. In case of random censoring, more subtle inverse probability of censoring weighting has to be applied, which will be considered at the end of this section. Define the corresponding at risk indicators for subject *i*, $Y_{\mathrm{CS},i}\left(t\right)=1\left\{i\in R_{\mathrm{CS}}\left(t\right)\right\}$ and $Y_{\mathrm{FG},i}\left(t\right)=1\left\{i\in R_{\mathrm{FG}}\left(t\right)\right\}$ and their sums $Y_{\mathrm{CS},•}\left(t\right)=\sum_{i=1}^{n}Y_{\mathrm{CS},i}\left(t\right)$ and $Y_{\mathrm{FG},•}\left(t\right)=\sum_{i=1}^{n}Y_{\mathrm{FG},i}\left(t\right)$.

A proportional hazards model for the cause‐specific hazards specifies, for instance for cause 1,

$$\alpha_{1}\left(t\;|\;x\right)=\alpha_{10}\left(t\right)\exp\left(\beta_{\mathrm{CS}}x\right).$$

The regression coefficient estimate is found by maximizing the partial likelihood

$$L_{\mathrm{CS}}\left(\beta \right)=\prod_{j:t_{j}^{*}\in D_{1}}\frac{\exp(\beta x_{j})}{\sum_{i}Y_{\mathrm{CS},i}\left(t_{j}^{*}\right)\exp\left(\beta x_{i}\right)},$$

where the product is over all cause 1 event time points $t_{j}^{*}$. In practice this partial likelihood can be maximized by standard Cox software by censoring the competing events.

The Fine–Gray approach is based on the wish to obtain a simple model for the cumulative incidence. It specifies a proportional hazards model for the subdistribution hazard, given by

(7) $$\lambda \left(t\;|\;x\right)=\lambda_{0}\left(t\right)\exp\left(\beta_{\mathrm{FG}}x\right).$$

The regression parameter $\beta_{\mathrm{FG}}$ is estimated using partial likelihood on the extended risk set, so including individuals that have experienced the competing event. The partial likelihood that is being maximized is given by

$$L_{\mathrm{FG}}\left(\beta \right)=\prod_{j:t_{j}^{*}\in D_{1}}\frac{\exp(\beta x_{j})}{\sum_{i}Y_{\mathrm{FG},i}\left(t_{j}^{*}\right)\exp\left(\beta x_{i}\right)}.$$

### 3.2 Non‐parametric estimation

In the multistate model, an estimate of r(t) is obtained implicitly from the models for $\alpha_{1}\left(t\right)$ and $\alpha_{2}\left(t\right)$. Here, we explore the possibility of estimating it directly from the data. Some feeling for how this works can be obtained by looking at the simplest case where there are no covariates and one is only interested in estimating the cumulative incidence function. The non‐parametric estimates of $\alpha_{1}\left(t\right)$ and λ(t) are defined at the same time points, namely the set of all cause 1 event time points, denoted by $D_{1}$. Let $t_{j}^{*}\in D_{1}$. The Nelson–Aalen estimate of the cause‐specific hazard at that point (assuming no ties) is given by

$${\hat{\alpha }}_{1}\left(t_{j}^{*}\right)=\frac{1}{\#\{T_{i}\geq t_{j}^{*}\}}=\frac{1}{Y_{\mathrm{CS},•}\left(t_{j}^{*}\right)}.$$

The reduction factor can be estimated by

(8) $$\hat{r}\left(t_{j}^{*}\right)=\frac{\#\{T_{i}\geq t_{j}^{*}\}}{\#\{T_{i}\geq t_{j}^{*}\;\text{or}\;D_{i}\neq 1\}}=\frac{Y_{\mathrm{CS},•}\left(t_{j}^{*}\right)}{Y_{\mathrm{FG},•}\left(t_{j}^{*}\right)},$$

which, using the relation $\lambda \left(t\right)=r\left(t\right)\alpha_{1}\left(t\right)$, leads to a Nelson–Aalen‐type estimator for the subdistribution hazard

$$\hat{\lambda }\left(t_{j}^{*}\right)=\frac{1}{\#\{T_{i}\geq t_{j}^{*}\;\text{or}\;D_{i}\neq 1\}}=\frac{1}{Y_{\mathrm{FG},•}\left(t_{j}^{*}\right)}.$$

So, in this situation the result is the same as a Nelson–Aalen estimator based directly on the Fine–Gray risk set. The only advantage is that the hazard estimate ${\hat{\alpha }}_{1}\left(t_{j}^{*}\right)$ is based on the risk set $R_{\mathrm{CS}}\left(t_{j}^{*}\right)$ where all individuals are at risk for the event and avoids the use of the risk set $R_{\mathrm{FG}}\left(t_{j}^{*}\right)$ where it is known that some individuals cannot have the event because they already experienced the competing event.

Remark 2 As seen from (2), the subdistribution hazard depends on the past through r(t) and on the future through $\alpha_{1}\left(t\right)$. That makes that is of no use in dynamic models where the conditioning is on T≥s and not on “T≥s or a competing event having happened before time *s*.” Cause‐specific hazards are much more natural in this context; the conditional cumulative incidence function $F_{1}\left(t\;|\;s\right)=P\left(T\leq t,D=1\;|\;T>s\right)$ depends naturally on both cause‐specific hazards, between *s* and *t*, through

$$F_{1}\left(t\;|\;s\right)=\int_{s}^{t}\alpha_{1}\left(u\right)\exp\left[-\int_{s}^{u}\left\{\alpha_{1}\left(v\right)+\alpha_{2}\left(v\right)\right\}dv\right]du.$$

In terms of the subdistribution hazard, one would have to “forget” the competing risks that occurred before time *s*. One could work with

$$F_{1}\left(t\;|\;s\right)=\exp\left\{-\int_{s}^{t}\lambda \left(u\;|\;s\right)du\right\},$$

where (cf. Equation (2))

$$\lambda \left(t\;|\;s\right)=r\left(t\;|\;s\right)\alpha_{1}\left(t\right),\mathrm{with}r\left(t\;|\;s\right)=\frac{P(Z(t)=0\;|\;Z(s)=0)}{P(Z(t)=0\;|\;Z(s)=0)+P(Z(t)=2\;|\;Z(s)=0)}.$$

Dynamic modeling using subdistribution hazards would basically require reestimation of r(t|s) for all time points *s* where dynamic prediction probabilities are required. For dynamic modeling of cumulative incidence, it is more practical to use landmarking (Cortese & Andersen, 2010; Cortese et al., 2013), possibly combined with pseudo‐values (Nicolaie, van Houwelingen, de Witte, & Putter, 2013b) instead.

### 3.3 Covariates

In case of a categorical covariate *x*, the richest model for r(t|x) would be obtained by separate non‐parametric estimates for each possible value *c* of the covariate, as discussed above. If we let $Y_{\mathrm{CS},i}^{c}\left(t\right)=Y_{\mathrm{CS},i}\left(t\right)1\left\{x_{i}=c\right\}$ and $Y_{\mathrm{FG},i}^{c}\left(t\right)=Y_{\mathrm{FG},i}\left(t\right)1\left\{x_{i}=c\right\}$ denote the at risk indicators for subjects with covariate value equal to *c*, a saturated model for r(t|x) would have as estimate

(9) $$\hat{r}\left(t_{j}^{*}\;|\;x=c\right)=\frac{\sum_{i=1}^{n}Y_{\mathrm{CS},i}^{c}\left(t_{j}^{*}\right)}{\sum_{i=1}^{n}Y_{\mathrm{FG},i}^{c}\left(t_{j}^{*}\right)},$$

for $t_{j}^{*}\in D_{1}$.

Remark 3 Interestingly, when *x* is a categorical covariate and when the estimate of the reduction factor r(t|x) is based on a saturated model, given by (9), it turns out that, in the absence of ties, the result of fitting a proportional hazards model on the cause‐specific hazard, using $-\log\{\hat{r}\left(t\;|\;x\right)\}$ as offset, suggested at the end of Section 2, is identical to the estimate obtained by maximizing the partial likelihood of the Fine–Gray model. This can be seen by starting out from the Fine–Gray partial likelihood, separating the terms in the Fine–Gray risk set according to the value of the covariates, and applying (9). We obtain

(10) $$\begin{array}{ccc} L_{\mathrm{FG}}\left(\beta \right) & = & \prod_{j\in D_{1}}\frac{\exp(\beta x_{j})}{\sum_{i}Y_{\mathrm{FG},i}\left(t_{j}^{*}\right)\exp\left(\beta x_{i}\right)}\\ & = & \prod_{j\in D_{1}}\frac{\exp(\beta x_{j})}{\sum_{i}\sum_{c}Y_{\mathrm{FG},i}^{c}\left(t_{j}^{*}\right)\exp\left(\beta c\right)}\\ & = & \prod_{j\in D_{1}}\frac{\exp(\beta x_{j})}{\sum_{c}\exp\left(\beta c\right)\sum_{i}Y_{\mathrm{CS},i}^{c}\left(t_{j}^{*}\right){\hat{r}}^{-1}\left(t_{j}^{*}\;|\;x_{i}=c\right)}\\ & = & \prod_{j\in D_{1}}\frac{\exp(\beta x_{j})}{\sum_{i}Y_{\mathrm{CS},i}\left(t_{j}^{*}\right)\exp\left(\beta x_{i}\right){\hat{r}}^{-1}\left(t_{j}^{*}\;|\;x_{i}\right)}. \end{array}$$

This sheds some light on the Fine–Gray riddle, in the sense that it shows that the Fine–Gray partial likelihood is not so unnatural as it may appear, and that it can be maximized based on the natural cause‐specific risk sets, using appropriate offsets.

Since r(t) has all the properties of a probability, an obvious way to start fitting more parsimonious models for r(t|x) is with a binomial GLM. Fix $t_{j}^{*}\in D_{1}$, and let $R_{\mathrm{FG}}\left(t_{j}^{*}\right)$ be the extended risk set at $t_{j}^{*}$. Within that set let $Y_{i}=1\left\{T_{i}\geq t_{j}^{*}\right\}$. Then $P\left(Y_{i}=1\;|\;x_{i}\right)=r\left(t_{j}^{*}\;|\;x_{i}\right)$. For a given link function *g*, we can postulate the model

$$g\left\{P\left(Y_{i}=1\;|\;x_{i}\right)\right\}=\gamma_{0}\left(t_{j}^{*}\right)+\gamma x_{i}.$$

Models for the different risk sets can be joined by specifying a parametric function or using splines for the “baseline” $\gamma_{0}\left(t\right)$. Depending on the choice of link function, time‐varying effects γ(t) of the covariates can be incorporated by interactions of the covariates with (functions of) time. Practically, these models can be fitted using standard generalized estimating equations (GEE) software, where the risk sets, given by all time points where a cause 1 event occurs, are considered to be independent. Standard errors may be obtained by sandwiching.

Possible choices for link functions are log(p), $\mathrm{logit}\left(p\right)=\log\left(\frac{p}{1-p}\right)$, and cloglog(p)=log{−log(p)}. It is useful to discuss the pro's and cons of different link functions in the context of what they imply for the subdistribution hazards, in relation to the models for the cause‐specific hazards. We already saw that the log link has the advantage that combining it with a proportional hazards model for the cause‐specific hazards leads to a proportional hazards model for the subdistribution hazard. The disadvantage of the log link is that it cannot deal with r(t|x) for *t* close to 0, because of the restriction that r(0|x)=1 for all covariate values *x*. It would be advisable to use time‐dependent effects $\beta_{\mathrm{RF}}\left(t\right)$ of the covariates, preferably with the restriction $\beta_{\mathrm{RF}}\left(0\right)=0$. The logit and cloglog links can deal with probabilities close to 1 for *t* close to 0. The disadvantage of the logit and cloglog links is that combining it with a proportional cause‐specific hazards model will lead to non‐proportional models for the subdistribution hazard.

Finally, as argued in Stijnen and van Houwelingen (1993), we can also estimate the regression coefficients in a GLM model by acting as if *Y* has a Poisson distribution. The Poisson model with log link will share the same advantages and disadvantages of the binomial GLM with log link. They may be easier to fit than binomial GLM with log link because also expected values higher than one are allowed.

### 3.4 Censoring

Right censoring complicates estimation in the Fine–Gray regression model, and also estimation of the reduction factor. The reason is that we do not know whether a censored observation will belong to the Fine–Gray risk set after the time of censoring. The potential observations of subject *i* are the pair $\left({\tilde{T}}_{i},{\tilde{D}}_{i}\right)$, with ${\tilde{T}}_{i}$ the event time and ${\tilde{D}}_{i}$ the cause of failure. The event time is right censored by a censoring time $C_{i}$, with censoring function $G\left(t\right)=P\left(C_{i}>t\right)$. We now observe $T_{i}=\min\left({\tilde{T}}_{i},C_{i}\right)$ and the event indicator $D_{i}$, which equals ${\tilde{D}}_{i}$, in case $T_{i}\leq C_{i}$, or 0, in case $C_{i}<T_{i}$. We assume that *C* is stochastically independent of the pair $\left(\tilde{T},\tilde{D}\right)$ and *X*. Alternatively, it can be assumed that *T* is independent of *C*, conditional on covariates. In that case the estimate of the censoring distribution $\hat{G}\left(t\right)$ below depends on *X*. Define $\delta_{i}\left(t\right)=1\left\{C_{i}\geq \min\left\{T_{i},t\right\}\right\}$. Following Fine and Gray (1999), we introduce time‐dependent weights $w_{i}\left(t\right)=\delta_{i}\left(t\right)\hat{G}\left(t\right)/\hat{G}\left(\min\left\{T_{i}-,t\right\}\right)$ for subject *i* at time *t*, where $\hat{G}\left(t\right)$ is the reverse Kaplan–Meier estimate of the censoring distribution (or a consistent estimate of *G*, given *X*). The idea behind the weighting is that the usable observations are upweighted to compensate for the censored ones; the weighted observations give a representation of what would be observed in the case of no censoring. By definition, we have $w_{i}\left(t_{i}\right)=1$, for $t_{i}\in D_{1}$.

In the case of categorical covariates, we redefine (9) as

(11) $$\begin{array}{ccc} \hat{r}\left(t_{j}^{*}\;|\;x=c\right) & = & \frac{\sum_{i=1}^{n}Y_{\mathrm{CS},i}^{c}\left(t_{j}^{*}\right)w_{i}\left(t_{j}^{*}\right)}{\sum_{i=1}^{n}Y_{\mathrm{FG},i}^{c}\left(t_{j}^{*}\right)w_{i}\left(t_{j}^{*}\right)}\\ & = & \frac{\sum_{i=1}^{n}Y_{\mathrm{CS},i}^{c}\left(t_{j}^{*}\right)}{\sum_{i=1}^{n}Y_{\mathrm{CS},i}^{c}\left(t_{j}^{*}\right)+\sum_{i=1}^{n}\left\{Y_{\mathrm{FG},i}^{c}\left(t_{j}^{*}\right)-Y_{\mathrm{CS},i}^{c}\left(t_{j}^{*}\right)\right\}\hat{G}\left(t_{j}^{*}\right)/\hat{G}\left(T_{i}-\right)}. \end{array}$$

The latter equality follows because for $i\in R_{\mathrm{CS}}\left(t_{j}^{*}\right)$, we have $w_{i}\left(t_{j}^{*}\right)=1$, while for $i\in R_{\mathrm{FG}}\left(t_{j}^{*}\right)\setminus R_{\mathrm{CS}}\left(t_{j}^{*}\right)$, we have $w_{i}\left(t_{j}^{*}\right)=\hat{G}\left(t_{j}^{*}\right)/\hat{G}\left(T_{i}-\right)$. The GLM's can also be extended to incorporate these weights. Standard errors could be obtained using the methods outlined in the Appendix or through bootstrapping.

The equivalence, noted earlier (Remark 3), of the Fine–Gray partial likelihood estimator and the result of fitting a proportional hazards model on the cause‐specific hazard, using $-\log\{\hat{r}\left(t\;|\;x\right)\}$ as offset for categorical covariates, remains valid in the presence of right censoring. In Equation (10), each contribution of $Y_{\mathrm{FG},i}\left(t_{j}^{*}\right)$ and $Y_{\mathrm{CS},i}\left(t_{j}^{*}\right)$ needs to be multiplied by $w_{i}\left(t_{j}^{*}\right)$, and the argument remains valid. Note that inverse probability of censoring weighting is only needed at the stage of estimation of the reduction factor, not when maximizing the partial likelihood.

## 4 MODELING THE EFFECT OF COVARIATES ON THE CUMULATIVE INCIDENCE FUNCTION

There are a number of ways of modeling the effect of covariates on the cumulative incidence of a given cause. In this section, we focus on models based on the cause‐specific hazards, subdistribution hazards, and/or reduction factor. Other approaches exist, such as vertical modeling (Nicolaie, van Houwelingen, & Putter, 2013a), but they fall outside the scope of this paper. Approaches 1 and 3 below are standard; approaches 2 and 4 are included to illustrate the use of the reduction factor. An extensive investigation of the relative performance of the estimators is outside the scope of this paper.

1. **Multistate approach (Multistate)**: Model both cause‐specific hazards by a proportional hazards model
  $$\alpha_{k}\left(t\;|\;x\right)=\alpha_{k0}\left(t\right)\exp\left(\beta_{k}x\right),$$
  for k=1,2. Estimate the parameters β_1_ and β_2_, and the baseline cause‐specific hazards α_10_ and α_20_ by partial likelihood, using the cause‐specific hazards risk set. Using relation (1), the implied effect of the covariates on the cumulative incidence can be calculated. For given covariate values $x^{*}$, using the estimates ${\hat{\beta }}_{1}$, ${\hat{\beta }}_{2}$, ${\hat{\alpha }}_{10}\left(t\right)$, ${\hat{\alpha }}_{20}\left(t\right)$, we obtain ${\hat{\alpha }}_{k}\left(t\;|\;x^{*}\right)={\hat{\alpha }}_{k0}\left(t\right)\exp\left({\hat{\beta }}_{k}x^{*}\right)$, ${\hat{A}}_{k}\left(t\;|\;x^{*}\right)=\sum_{0<s\leq t}{\hat{\alpha }}_{k}\left(s\;|\;x^{*}\right)$, and finally
  $${\hat{F}}_{1}\left(t\;|\;x^{*}\right)=\sum_{0<s\leq t}{\hat{\alpha }}_{1}\left(s\;|\;x^{*}\right)\exp\left[-\{{\hat{A}}_{1}\left(s\;|\;x^{*}\right)+{\hat{A}}_{2}\left(s\;|\;x^{*}\right)\}\right].$$
  Since the cause‐specific hazards coincide with the transition hazards in the multistate model depicted in Figure 1a, this approach is known as the multistate approach to competing risks (Andersen, Abildstrom, & Rosthøj, 2002). It has been implemented in the multistate model package **mstate** (de Wreede, Fiocco, & Putter, 2010). The resulting effect of the covariates on the cumulative incidence may not always be increasing or decreasing in *x*.
2. **Subdistribution implied by cause‐specific hazard and reduction factor (Subdistribution through** r(t|x)): Use the cause‐specific hazards risk set. Model $\alpha_{1}\left(t\right)$ by a proportional hazards model, estimate the parameters by partial likelihood using the cause‐specific hazards risk set, as in approach 1. Model r(t) by some GLM. Fit it by GEE or a similar approach. Calculate, for a given value $x^{*}$ of the covariates,
  $$\hat{\lambda }\left(t\;|\;x^{*}\right)=\hat{r}\left(t\;|\;x^{*}\right){\hat{\alpha }}_{1}\left(t\;|\;x^{*}\right),\;\mathrm{and}\;{\hat{F}}_{1}\left(t\;|\;x^{*}\right)=1-\exp\left\{-\sum_{t_{j}^{*}\leq t}\hat{\lambda }\left(t_{j}^{*}\;|\;x^{*}\right)\right\}.$$
  This approach has not been implemented in statistical software. Example code is provided in the Supporting Information.
3. **Fine–Gray approach (Fine–Gray)**: The Fine–Gray model specifies a proportional hazards model for the subdistribution hazard, given by
  (12) $$\lambda \left(t\;|\;x\right)=\lambda_{0}\left(t\right)\exp\left(\beta_{\mathrm{FG}}x\right).$$
  After having estimated $\beta_{\mathrm{FG}}$ by maximizing the Fine–Gray partial likelihood, the cumulative incidence function is obtained by
  $$\begin{array}{ccc} {\hat{\lambda }}_{0}\left(t_{j}^{*}\right) & = & \frac{1}{\sum_{i}Y_{\mathrm{FG},i}\left(t_{j}^{*}\right)w_{i}\left(t_{j}^{*}\right)\exp\left({\hat{\beta }}_{\mathrm{FG}}x_{i}\right)},\\ \hat{\lambda }\left(t_{j}^{*}\;|\;x^{*}\right) & = & {\hat{\lambda }}_{0}\left(t_{j}^{*}\right)\exp\left({\hat{\beta }}_{\mathrm{FG}}x^{*}\right),\;{\hat{F}}_{1}\left(t\;|\;x^{*}\right)=1-\exp\left\{-\sum_{t_{j}^{*}\leq t}\hat{\lambda }\left(t_{j}^{*}\;|\;x^{*}\right)\right\}. \end{array}$$
  This approach has been implemented in the **cmprsk** package (Gray, 2014).
4. **Fine–Gray with cause‐specific hazards partial likelihood (Fine–Gray with CSH PL)**: The objective again is to have a simple relation for the subdistribution hazard, given by (12). The parameter $\beta_{\mathrm{FG}}$ is now estimated based on an initial estimate of r(t|x), and subsequently based on maximizing the cause‐specific partial likelihood with $-\log\{\hat{r}\left(t\;|\;x\right)\}$ as offset. By Remark 3, if *x* is categorical and the estimate of r(t|x) is based on a saturated model, then this approach yields the same estimate of $\beta_{\mathrm{FG}}$ as the Fine–Gray approach, and also the same cumulative incidence function for a given value $x^{*}$ of the covariates. If not, we obtain a different estimate ${\tilde{\beta }}_{\mathrm{FG}}$ of the same estimand $\beta_{\mathrm{FG}}$. The cumulative incidence for fixed covariate value $x^{*}$, ${\hat{F}}_{1}\left(t\;|\;x^{*}\right)$, can be obtained from the estimates of Equation (6) and those of r(t|x), by going back to Equation (2), and obtaining the subdistribution hazard as
  $$\hat{\lambda }\left(t_{j}^{*}\;|\;x^{*}\right)=\hat{r}\left(t_{j}^{*}\;|\;x^{*}\right)\cdot {\hat{\alpha }}_{1}\left(t_{j}^{*}\;|\;x^{*}\right),$$
  for each event time point $t_{j}^{*}$ of the cause of interest, which, by Equation (6), yields
  $$\hat{\lambda }\left(t_{j}^{*}\;|\;x^{*}\right)={\hat{\alpha }}_{0}\left(t_{j}^{*}\right)\exp\left({\tilde{\beta }}_{\mathrm{FG}}x^{*}\right).$$
  Since
  $${\hat{\alpha }}_{0}\left(t_{j}^{*}\right)=\frac{1}{\sum_{k\in R_{\mathrm{CS}}\left(t_{j}^{*}\right)}\exp({\tilde{\beta }}_{\mathrm{FG}}x_{k}-\log\left\{\hat{r}\left(t_{j}^{*}\;|\;x_{k}\right)\right\}},$$
  we obtain
  $$\hat{\lambda }\left(t_{j}^{*}\;|\;x^{*}\right)=\frac{\exp({\tilde{\beta }}_{\mathrm{FG}}x^{*})}{\sum_{k\in R_{\mathrm{CS}}\left(t_{j}^{*}\right)}\exp\left[{\tilde{\beta }}_{\mathrm{FG}}x_{k}-\log\left\{\hat{r}\left(t_{j}^{*}\;|\;x_{k}\right)\right\}\right]}.$$
  The cumulative incidence function is given by
  $${\hat{F}}_{1}\left(t\;|\;x^{*}\right)=1-\exp\left\{-\sum_{0<t_{j}^{*}\leq t}\hat{\lambda }\left(t_{j}^{*}\;|\;x^{*}\right)\right\},$$
  where the sum is over all cause 1 event time points before time *t*.
  This approach has not been implemented in statistical software. Example code is provided in the Supporting Information.

## 5 ILLUSTRATION

**Figure 5:**
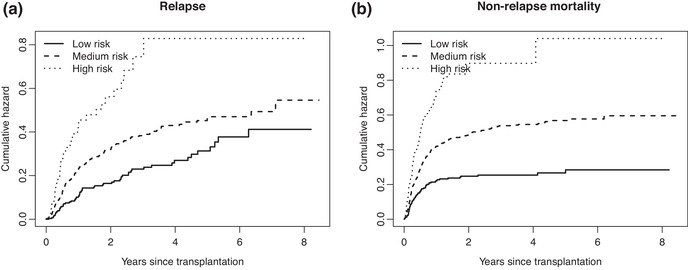
Non‐parametric estimates of the (a) cause‐specific hazards of relapse and (b) non‐relapse mortality, for each of the three EBMT risk score groups

We illustrate the different methods of Sections 3 and 4 on a data set of 1977 patients with chronic myeloid leukemia (CML), collected by the European Society for Blood and Marrow Transplantation (EBMT), and available in the **mstate** package (de Wreede et al., 2010) as data set ebmt1. After removing the children (age below 18) from the data, 1835 patients remain. The competing risks involved are relapse (421 events), and death before relapse (641 events), more commonly called non‐relapse mortality (NRM). The remaining 773 observations are right censored. The data contain ties, these have been broken for the analysis. The covariates that are used here for illustration are age (centered at 40, reported per decade for analysis) and the EBMT risk score, which is a grouping into “Low risk” (n=321), “Medium risk” (n=1349), and “High risk” (n=165). The age range was 18–64, with a median of 36. Figure 5 shows the (a) estimated cumulative hazards for relapse and (b) non‐relapse mortality, per risk group.

They are comparable in terms of their values at the end of follow‐up and in terms of differences between the risk groups, but the shape of the cumulative hazards is somewhat different; those for non‐relapse mortality increase more steeply in the beginning, while those of relapse increase more gradually. In terms of the early, middle, and late risk terminology, used in Section 2, non‐relapse mortality would correspond to early risk and relapse would tend more toward middle risk. The EBMT risk score has comparable effects on both causes of failure. Proportional hazards models on the cause‐specific hazards yields, with age and risk group with “Low risk” as reference category, regression coefficients (standard errors) of 0.476 (0.149) and 1.139 (0.205) for “Medium risk” and “High risk,” respectively and 0.002 (0.053) for age (per decade) for relapse, and 0.658 (0.134), 1.206 (0.173) and 0.040 (0.043), again for “Medium risk,” “High risk” and age (per decade), respectively for non‐relapse mortality.

**Figure 6:**
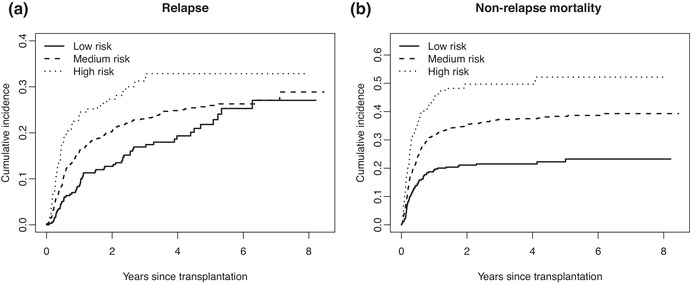
Non‐parametric estimates of the (a) cumulative incidences of relapse and (b) non‐relapse mortality, for each of the three EBMT risk score groups

Figure 6 shows the (a) estimated cumulative incidence curves for relapse and (b) non‐relapse mortality, separately for each risk group.

Relapse seems to be more affected by the competing risk NRM than reversely; the effect of the EBMT risk score is now seen to be smaller for relapse than for NRM. Fine–Gray regression with age and risk group yields regression coefficients (standard errors) of 0.287 (0.147), 0.619 (0.208), and −0.012 (0.056) for “Medium risk,” “High risk,” and age (decade), respectively for relapse, and 0.584 (0.135), 1.004 (0.174), and 0.041 (0.042), again for “Medium risk,” “High risk” and age (decade), respectively for non‐relapse mortality. The fact that, compared to the cause‐specific log hazard ratios, the Fine–Gray estimates for relapse are much more reduced than those for NRM, can be understood by noting that relapse tends more toward later risk than NRM.

**Figure 7:**
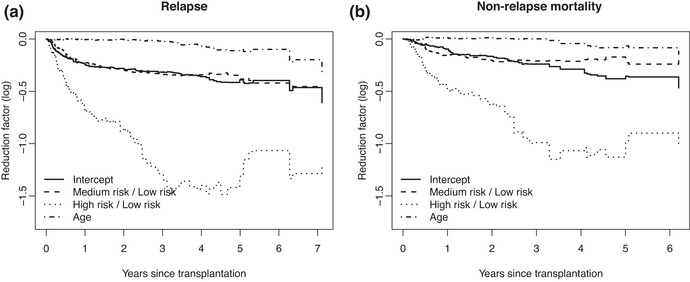
Estimates of the baseline and effects of score and age on the log reduction factor for (a) relapse and (b) non‐relapse mortality

To model the reduction factor we fitted, separately for event time point $t_{j}^{*}$ of the cause of interest, a Poisson GLM model with

$$\log\left\{E\left(Y_{t_{j}^{*}}|x\right)\right\}=\log\left\{P\left(Y_{t_{j}^{*}}=1|x\right)\right\}=\gamma_{j0}+\gamma_{j1}x_{1}+\gamma_{j2}x_{2}+\gamma_{j3}x_{3},$$

with $Y_{t_{j}^{*}}$ defined (with all subjects in the Fine–Gray risk set at $t_{j}^{*}$) as 1 if also in the cause‐specific hazards risk set at $t_{j}^{*}$ and 0 otherwise, *x*_1_ and *x*_2_ the dummy variables for medium and high risk scores (compared to low risk) and *x*_3_ age, centered by 40, by decade. Inverse probability of censoring weights, described in Section 3, have been used in this analysis. Figure 7 shows the estimates of $\gamma_{j0}$ (Intercept), and of $\gamma_{j1}$ (Medium risk / Low risk), $\gamma_{j2}$ (High risk / Low risk) and $\gamma_{j3}$ (Age) for relapse (a) and non‐relapse mortality (b).

Recalling Equation (5), the fact that for instance the $\gamma \left(t\right)=\beta_{\mathrm{RF}}\left(t\right)$ for High risk / Low risk is quite negative implies that the Fine–Gray regression coefficient will be lower than the corresponding regression coefficient for the cause‐specific hazards for this factor.

**Figure 8:**
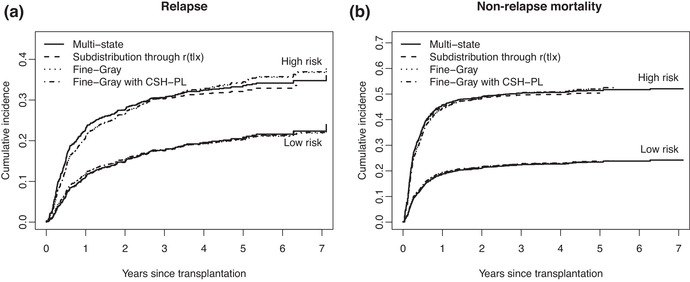
Estimates of the cumulative incidences of (a) relapse and (b) non‐relapse mortality for the low and high risk groups and age equal to 40, based on four modeling approaches

Finally, Figure 8 shows the estimates of the cumulative incidences of (a) relapse and (b) non‐relapse mortality for the two extreme risk groups (the middle one was omitted because no real differences could be seen) and age equal to 40, based on the four modeling approaches of Section 4.

Note that the model we used for estimating the reduction factor was almost saturated (it would be if age was not included), since we have full interactions of all covariates with time. This causes the curves for Fine–Gray (method 3) and “Fine–Gray with CSH‐PL” (method 4) to be almost indiscernible. The regression coefficients for “Fine–Gray with CSH‐PL” were almost identical to Fine–Gray (results can be seen in the Supporting Information). The underlying assumptions for Fine–Gray (and “Fine–Gray with CSH‐PL”) and “Multistate” (method 1) are different. Both assume proportional hazards, but Fine–Gray assumes proportional hazards on the subdistribution hazards, while “Multistate” assumes proportional hazards on the cause‐specific hazards. The difference in the shape of the cumulative incidence curves for the high risk group can be clearly seen. Looking at Figures 5 and 6a, one could question the validity of the proportional hazards assumption, both for the cause‐specific hazards and for the subdistribution hazard of relapse. Despite the apparent violation of the proportional hazards assumption for the cause‐specific hazard and subdistribution hazard of relapse, we chose to retain the proportional models, because it illustrates the influence of the proportional hazards assumptions on the estimated cumulative incidences. The “Subdistribution through r(t|x)” method (method 2) is close to the proportional subdistribution hazards approach, in terms of assumptions. The cause‐specific hazards have been estimated based on a proportional hazards assumption on the cause‐specific hazards, but subsequently subdistribution hazards are obtained by multiplying with an estimate of the reduction factor, where this estimate does not make that assumption (nor of proportionality on the subdistribution hazard).

**Figure 9:**
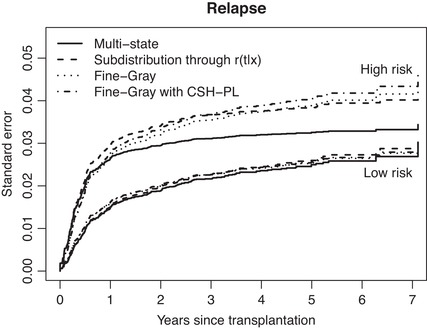
Estimates of the standard errors of the cumulative incidences of relapse for the low and high risk groups and age equal to 40, based on four modeling approaches

Figure 9 shows pointwise standard errors of the four model‐based cumulative incidence curves of relapse, also for the low and high risk groups and age equal to 40. They are quite similar, with the exception of the cumulative incidence curves for relapse for high risk based on the multistate model, which shows lower standard errors than the other curves from one year after stem cell transplantation onward.

## 6 DISCUSSION

Our primary aim in this paper was to study the relation between the cause‐specific hazards and the subdistribution hazard. For this purpose, we defined the reduction factor as the ratio between subdistribution hazard and cause‐specific hazard. The effect of a covariate on the log reduction factor (possibly time‐varying) quantifies the difference between the regression coefficients obtained by Fine–Gray regression and Cox regression on the cause‐specific hazard.

Estimating and modeling the reduction factor offers alternative ways of modeling the cumulative incidence function as a function of covariates. One attractive approach is to model both the cause‐specific hazard and the reduction factor and obtain the subdistribution hazard as the product of these. It is not quite clear at present what are the properties of this estimator, especially in situations where the proportional hazards assumption on cause‐specific hazard and/or subdistribution hazard is violated. In general we recommend making the models for the reduction factor rich, as we did in the application, for instance separate Poisson GLM's for each time point. Although the target for estimation is very different, the modeling approach resembles that for additive hazards. Reasons for the recommendation of making rich models are given in the following two paragraphs.

We showed that a Fine–Gray‐type coefficient can be obtained by using the negative logarithm of the estimated reduction factor as time‐dependent offset in a cause‐specific hazards model. For a single categorical covariate, when the model for the reduction factor is fully saturated, in other words when separate non‐parametric estimates of the reduction factor for each level of the covariate are used, maximizing the partial likelihood of the cause‐specific hazard with the negative logarithm of this estimated reduction factor as offset, yields exactly the Fine–Gray estimator, maximizing the Fine–Gray partial likelihood. Thus, in a sense this solves the Fine–Gray riddle: the reduction factor is also the adjustment factor needed to justify the Fine–Gray likelihood.

In the Appendix, we outline how to obtain standard errors for the estimators of cumulative incidence functions given covariates, for the four methods discussed in Section 4. Methods 2 and 4 use an initial estimate of the reduction factor r(t|x), given the covariates. We derive standard errors of the cumulative incidences given covariates, ignoring the uncertainty in the estimate of r(t|x). Despite our recommendation to make the models for r(t|x) very rich, we think that as far as the standard errors of the estimates of the parameters of the reduction factor are concerned, the uncertainty in these estimates can be ignored in practice, but this requires further study. Using standard Cox software for the cause‐specific hazards with the negative logarithm of the reduction factor as offset, we obtain very similar (although not exactly identical) estimates of the standard errors of the Fine–Gray coefficients, compared to the results from Fine–Gray, see Supporting Information. The situation is not unlike marginal structural models or propensity score matching, where models for inverse probability weights and propensity scores are also advised to be sufficiently rich, and where uncertainty in these intermediate models is negligible for the end results where the results of these models are used for weighting or adjusting/matching. More research is needed to assess the behaviour of the estimators of the cumulative incidence functions and their estimated standard errors, in terms of accuracy and coverage.

## CONFLICT OF INTEREST

The authors have declared no conflict of interest.

## Supporting information

Supporting Information

## Appendix

In this appendix, we outline how asymptotic standard errors of the cumulative incidence for given covariates *x* can be obtained, for the four methods in Section 4. We do not discuss the first approach (multistate) in detail, since asymptotic theory for this approach can be found in textbooks (Beyersmann et al., 2011; Geskus, 2015) and has been implemented in *mstate* (de Wreede et al., 2010). Calculation of the standard errors in the other three cases follows a very similar structure that is well known (Andersen, Borgan, Gill, & Keiding, 1993; Therneau & Grambsch, 2000; van Houwelingen & Putter, 2012). Throughout, we ignore the uncertainty in the estimation of the reduction factor r(t|x), see Discussion. We also do not specify how r(t|x) is modeled, but we assume that the model is correctly specified and that r(t|x) is consistently estimated for all *x*. Key for all three remaining methods is to derive the asymptotic variance of

$$-\log\left\{1-{\hat{F}}_{1}\left(t\;|\;x^{*}\right)\right\}=\sum_{0<t_{j}^{*}\leq t}\hat{\lambda }\left(t_{j}^{*}\;|\;x^{*}\right),$$

the sum being over all cause 1 event time points before time *t*. These can be used to construct confidence intervals for ${\hat{F}}_{1}\left(t\;|\;x^{*}\right)$ on the log‐scale, or on the probability scale, after applying the delta method, which yields

$$\mathrm{Var}\left\{{\hat{F}}_{1}\left(t\;|\;x^{*}\right)\right\}={\left\{1-{\hat{F}}_{1}\left(t\;|\;x^{*}\right)\right\}}^{2}\;\mathrm{Var}\left[\log\left\{1-{\hat{F}}_{1}\left(t\;|\;x^{*}\right)\right\}\right].$$

The estimates of $\hat{\lambda }\left(t_{j}^{*}\;|\;x^{*}\right)$ are obtained in different ways for the three remaining methods, leading to slightly different ways of deriving the asymptotic variance of $\sum_{0<t_{j}^{*}\leq t}\hat{\lambda }\left(t_{j}^{*}\;|\;x^{*}\right)$.

### Subdistribution through r(t|x)

Denote the estimate of $\beta_{\mathrm{CS}}$ in the proportional hazards model for the cause‐specific hazard of cause 1 as ${\hat{\beta }}_{\mathrm{CS}}$. The subdistribution rate $\hat{\lambda }\left(t\;|\;x^{*}\right)$ is estimated as the product of $\hat{r}\left(t\;|\;x^{*}\right)$ and ${\hat{\alpha }}_{1}\left(t\;|\;x^{*}\right)$.

Define ${\bar{x}}_{\mathrm{CS}}\left(t_{j}^{*}\right)$ as a weighted average of the *x*'s in the risk set $R_{\mathrm{CS}}\left(t_{j}^{*}\right)$, that is

$${\bar{x}}_{\mathrm{CS}}\left(t_{j}^{*}\right)=\frac{\sum_{k\in R_{\mathrm{CS}}\left(t_{j}^{*}\right)}x_{k}\exp\left({\hat{\beta }}_{\mathrm{CS}}x_{k}\right)}{\sum_{k\in R_{\mathrm{CS}}\left(t_{j}^{*}\right)}\exp\left({\hat{\beta }}_{\mathrm{CS}}x_{k}\right)}.$$

The Fisher information of the partial likelihood, assuming no ties, is given by $I_{\mathrm{pl}}\left({\hat{\beta }}_{\mathrm{CS}}\right)=\sum_{j}{\mathrm{Var}}_{\mathrm{CS}}\left(t_{j}^{*}\right)$, with

$${\mathrm{Var}}_{\mathrm{CS}}\left(t_{j}^{*}\right)=\frac{\sum_{k\in R_{\mathrm{CS}}\left(t_{j}^{*}\right)}\left\{x_{k}-{\bar{x}}_{\mathrm{CS}}\left(t_{j}^{*}\right)\right\}{\left\{x_{k}-{\bar{x}}_{\mathrm{CS}}\left(t_{j}^{*}\right)\right\}}^{⊤}\exp\left({\hat{\beta }}_{\mathrm{CS}}x_{k}\right)}{\sum_{k\in R_{\mathrm{CS}}\left(t_{j}^{*}\right)}\exp\left({\hat{\beta }}_{\mathrm{CS}}x_{k}\right)}$$

the weighted covariance matrix in the risk set at event time $t_{j}^{*}$. The asymptotic variance of $-\log\left\{1-{\hat{F}}_{1}\left(t\;|\;x^{*}\right)\right\}=\sum_{0<t_{j}^{*}\leq t}\hat{\lambda }\left(t_{j}^{*}\;|\;x^{*}\right)$ may now be estimated consistently by

∑tj∗≤ttj∗∈D1r^(tj∗|x∗)exp(β^CSx∗)∑k∈RCS(tj∗)exp(β^CSxk)2+q^(t|x∗)⊤Ipl−1(β^CS)q^(t|x∗),

with

q^(t|x∗)=∑tj∗≤ttj∗∈D1{x∗−x¯CS(tj∗)}r^(tj∗|x∗)exp(β^CSx∗)∑k∈RCS(tj∗)exp(β^CSxk).

### Fine–Gray

The Fine–Gray model is based on the partial likelihood of the Fine–Gray risk set, using inverse probability of censoring weighting to account for the censoring. Recall the time‐dependent weights $w_{i}\left(t\right)=\delta_{i}\left(t\right)\hat{G}\left(t\right)/\hat{G}\left(\min\left\{T_{i}-,t\right\}\right)$ for subject *i* at time *t*, where $\hat{G}\left(t\right)$ is the reverse Kaplan–Meier estimate of the censoring distribution. Let ${\hat{\beta }}_{\mathrm{FG}}$ be the Fine–Gray estimate of the effect of *x* on the cumulative incidence of cause 1. The formulas need to be adjusted in the following way.

Define ${\bar{x}}_{\mathrm{FG}}\left(t_{j}^{*}\right)$ as a weighted average of the *x*'s in the risk set $R_{\mathrm{FG}}\left(t_{j}^{*}\right)$, that is

$${\bar{x}}_{\mathrm{FG}}\left(t_{j}^{*}\right)=\frac{\sum_{k\in R_{\mathrm{FG}}\left(t_{j}^{*}\right)}x_{k}\exp\left({\hat{\beta }}_{\mathrm{FG}}x_{k}\right)w_{k}\left(t_{j}^{*}\right)}{\sum_{k\in R_{\mathrm{FG}}\left(t_{j}^{*}\right)}\exp\left({\hat{\beta }}_{\mathrm{FG}}x_{k}\right)w_{k}\left(t_{j}^{*}\right)}.$$

The Fisher information of the partial likelihood, assuming no ties, is given by $I_{\mathrm{pl}}\left({\hat{\beta }}_{\mathrm{FG}}\right)=\sum_{j}{\mathrm{Var}}_{\mathrm{FG}}\left(t_{j}^{*}\right)$, with

$${\mathrm{Var}}_{\mathrm{FG}}\left(t_{j}^{*}\right)=\frac{\sum_{k\in R_{\mathrm{FG}}\left(t_{j}^{*}\right)}\left\{x_{k}-{\bar{x}}_{\mathrm{FG}}\left(t_{j}^{*}\right)\right\}{\left\{x_{k}-{\bar{x}}_{\mathrm{FG}}\left(t_{j}^{*}\right)\right\}}^{⊤}\exp\left({\hat{\beta }}_{\mathrm{FG}}x_{k}\right)w_{k}\left(t_{j}^{*}\right)}{\sum_{k\in R_{\mathrm{FG}}\left(t_{j}^{*}\right)}\exp\left({\hat{\beta }}_{\mathrm{FG}}x_{k}\right)w_{k}\left(t_{j}^{*}\right)}$$

the weighted covariance matrix in the risk set at event time $t_{j}^{*}$. The asymptotic variance of $-\log\left\{1-{\hat{F}}_{1}\left(t\;|\;x^{*}\right)\right\}=\sum_{0<t_{j}^{*}\leq t}\hat{\lambda }\left(t_{j}^{*}\;|\;x^{*}\right)$ may now be estimated consistently by

∑tj∗≤ttj∗∈D1exp(β^FGx∗)∑k∈RFG(tj∗)exp(β^FGxk)wk(tj∗)2+q^(t|x∗)⊤Ipl−1(β^FG)q^(t|x∗),

with

q^(t|x∗)=∑tj∗≤ttj∗∈D1{x∗−x¯FG(tj∗)}exp(β^FGx∗)∑k∈RFG(tj∗)exp(β^FGxk)wk(tj∗).

### Fine–Gray with CSH PL

Define ${\tilde{x}}_{\mathrm{CS}}\left(t_{j}^{*}\right)$ as a weighted average of the *x*'s in the risk set $R_{\mathrm{CS}}\left(t_{j}^{*}\right)$, accounted for the offset, that is

$${\tilde{x}}_{\mathrm{CS}}\left(t_{j}^{*}\right)=\frac{\sum_{k\in R_{\mathrm{CS}}\left(t_{j}^{*}\right)}x_{k}\exp\left[{\tilde{\beta }}_{\mathrm{FG}}x_{k}-\log\left\{\hat{r}\left(t_{j}^{*}\;|\;x_{k}\right)\right\}\right]}{\sum_{k\in R_{\mathrm{CS}}\left(t_{j}^{*}\right)}\exp\left[{\tilde{\beta }}_{\mathrm{FG}}x_{k}-\log\left\{\hat{r}\left(t_{j}^{*}\;|\;x_{k}\right)\right\}\right]}.$$

The Fisher information of the partial likelihood, assuming no ties, is given by $I_{\mathrm{pl}}\left({\tilde{\beta }}_{\mathrm{FG}}\right)=\sum_{j}{\tilde{\mathrm{Var}}}_{\mathrm{CS}}\left(t_{j}^{*}\right)$, with

$${\tilde{\mathrm{Var}}}_{\mathrm{CS}}\left(t_{j}^{*}\right)=\frac{\sum_{k\in R_{\mathrm{CS}}\left(t_{j}^{*}\right)}\left\{x_{k}-{\tilde{x}}_{\mathrm{CS}}\left(t_{j}^{*}\right)\right\}{\left\{x_{k}-{\tilde{x}}_{\mathrm{CS}}\left(t_{j}^{*}\right)\right\}}^{⊤}\exp\left[{\tilde{\beta }}_{\mathrm{FG}}x_{k}-\log\left\{\hat{r}\left(t_{j}^{*}\;|\;x_{k}\right)\right\}\right]}{\sum_{k\in R_{\mathrm{CS}}\left(t_{j}^{*}\right)}\exp\left[{\tilde{\beta }}_{\mathrm{FG}}x_{k}-\log\left\{\hat{r}\left(t_{j}^{*}\;|\;x_{k}\right)\right\}\right]}$$

the weighted covariance matrix in the risk set at event time $t_{j}^{*}$. The asymptotic variance of $-\log\left\{1-{\hat{F}}_{1}\left(t\;|\;x^{*}\right)\right\}=\sum_{0<t_{j}^{*}\leq t}\hat{\lambda }\left(t_{j}^{*}\;|\;x^{*}\right)$ may now be estimated consistently by

∑tj∗≤ttj∗∈D1exp(β∼FGx∗)∑k∈RCS(tj∗)exp[β∼FGxk−log{r^(tj∗|xk)}]2+q^(t|x∗)⊤Ipl−1(β∼FG)q^(t|x∗),

with

q^(t|x∗)=∑tj∗≤ttj∗∈D1{x∗−x∼CS(tj∗)}exp(β∼FGx∗)∑k∈RCS(tj∗)exp[β∼FGxk−log{r^(tj∗|xk)}].
