# Supplementary material for: On the relation between the cause‐specific hazard and the subdistribution rate for competing risks data: The Fine–Gray model revisited
Source: Biom J. 2020 Mar 4;62(3):790–807. doi: 10.1002/bimj.201800274 (PMC7216972; doi:10.1002/bimj.201800274)
Supplement: Supplementary file 1 — Supporting Information [file BIMJ-62-790-s001.zip › EBMT1.pdf]

EBMT data example

Hein Putter

23 September, 2019

1 Introduction

This is an online companion to the paper "On the relation betwen the cause-specific hazard and the subdistribution rate for competing risks data: the Fine-Gray model revisited". It provides a data example of estimating and modeling the reduction factor. The data are from the EBMT, in the `mstate` package, data set `ebmt1`.

1.1 Covariates

There are two covariates in the data, age and the EBMT risk score, which is a risk score that was developed for relapse, but it is known that it is also predictive of non-relapse mortality. Originally it has 7 levels, but this data has only three levels, denoted as Low risk, Medium risk and High risk (we don't have the finer version).

Descriptive statistics of age, along with a histogram, is shown below.

```
##    Min. 1st Qu.  Median    Mean 3rd Qu.    Max.
##    0.00   28.00   36.00   35.81   45.00   64.00
```

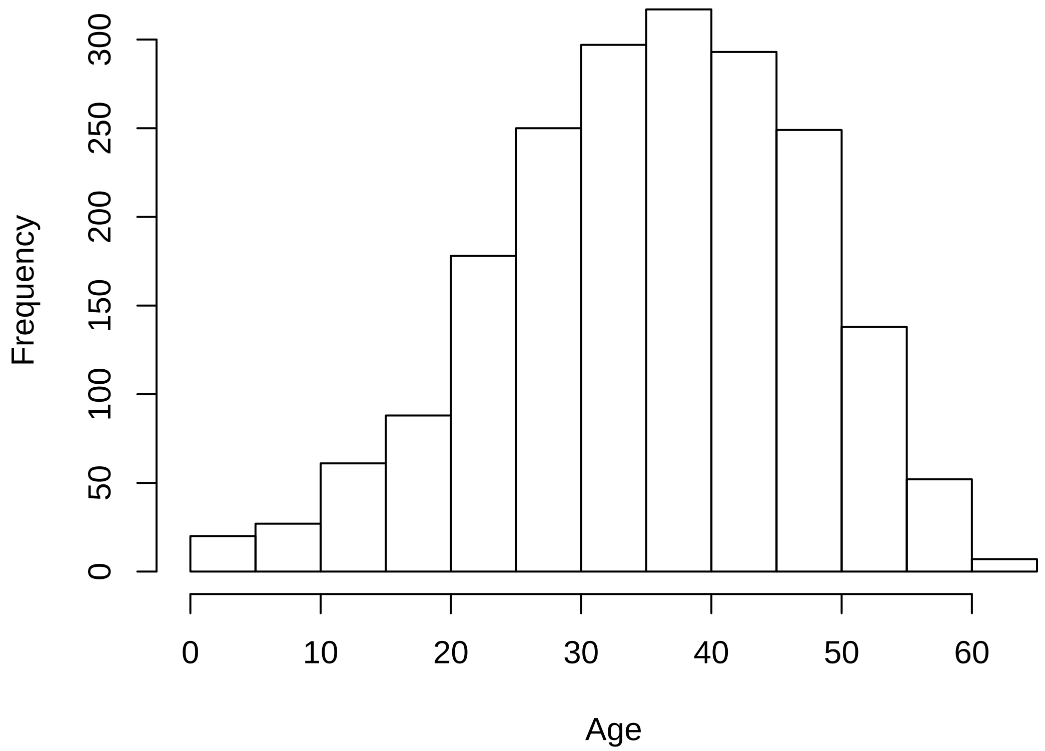

We remove subjects younger than age 18, and redo summary and histogram without those.

```
##    Min. 1st Qu.  Median    Mean 3rd Qu.    Max.
##   18.00   30.00   38.00   37.69   45.00   64.00
```

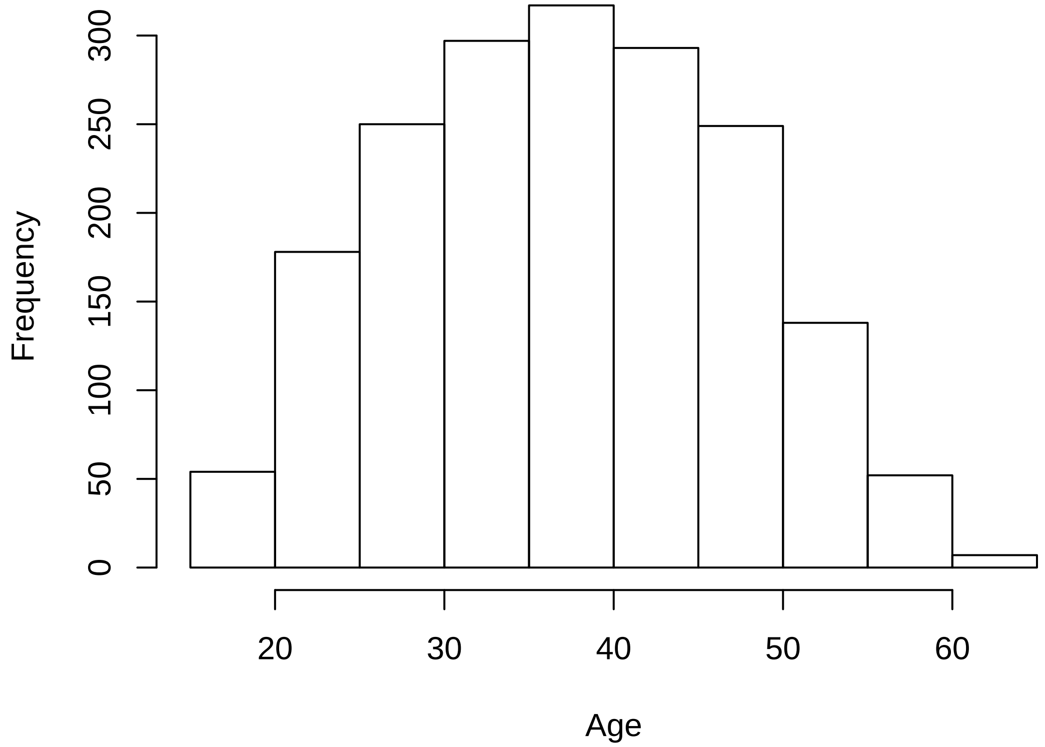

The working covariate will be `age10`, which is age, centered at 40, divided by 10 (so effects of age are per decade).

The second covariate is `score`. Here is a frequency table.

```
##
##    Low risk Medium risk   High risk
##         321        1349         165
```

1.2 Competing risks descriptive

The competing risks involved are relapse, and death before relapse, called non-relapse mortality or NRM in the field.

```
##
##                Censored                Relapse Non-relapse mortality
##                     773                    421                   641
```

In the data set there are ties, these are broken before continuing.

2 Non-parametric estimation

2.1 Cumulative hazards

We start with plots of the non-parametric estimates of the cause-specific hazards for each of the three risk scores.

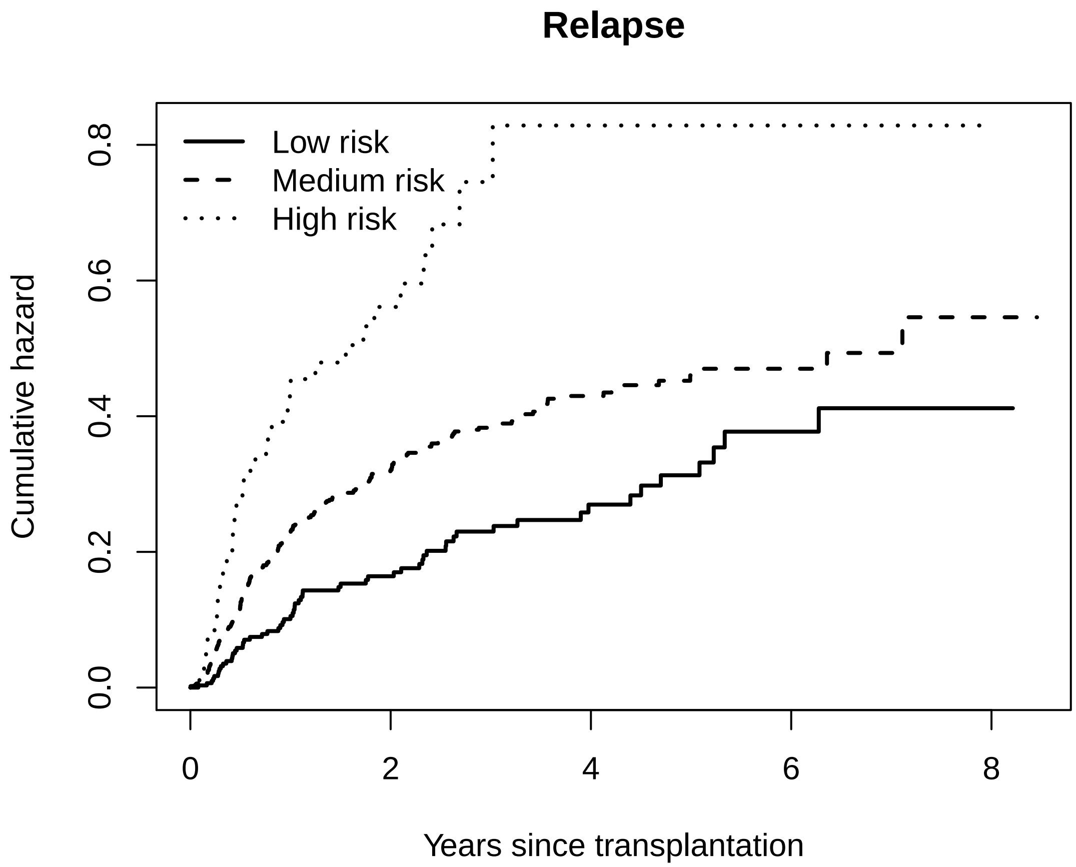
Relapse
Low risk
Medium risk
High risk
Cumulative hazard
0.0
0.2
0.4
0.6
0.8
0
2
4
6
8
Years since transplantation

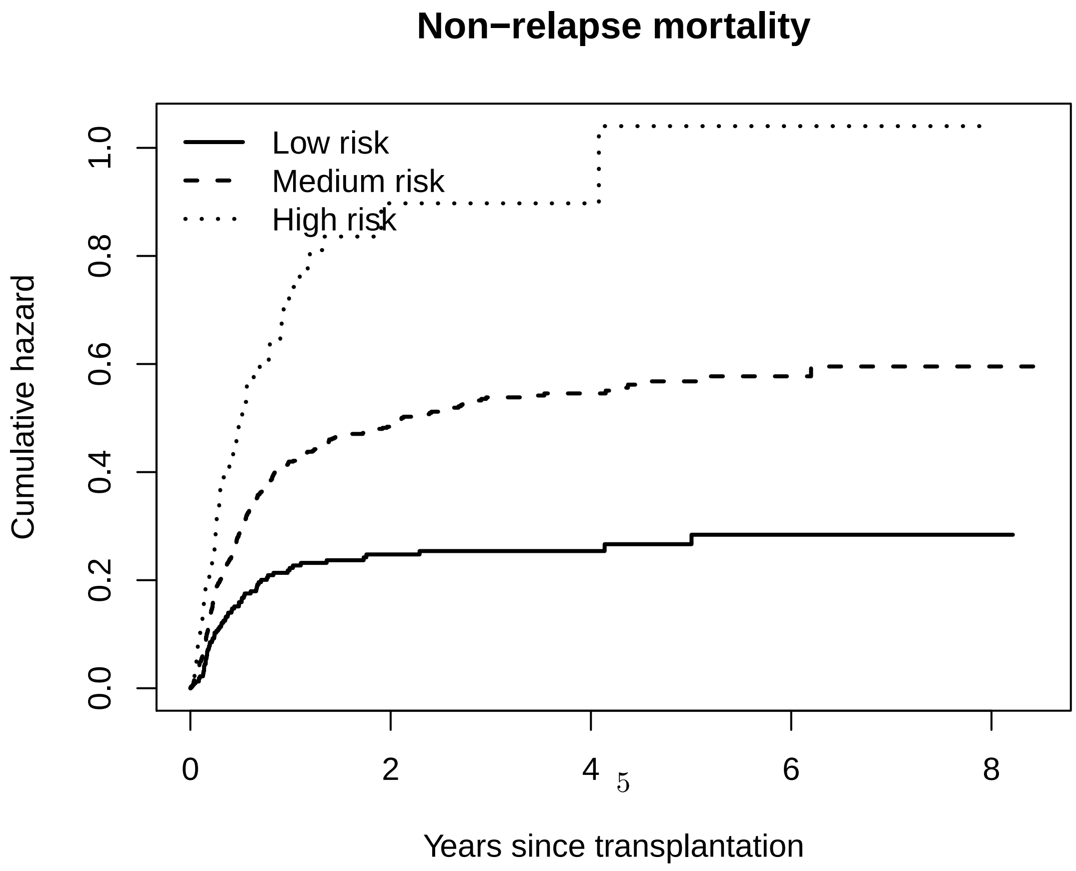
Non−relapse mortality
Low risk
Medium risk
High risk
Cumulative hazard
0.0
0.2
0.4
0.6
0.8
1.0
0
2
4
6
8
Years since transplantation

Cox models for the cause-specific hazards give the following. Only the three risk groups (first cause 1, then cause 2).

```
## Call:
## coxph(formula = Surv(rfs, rfsstat == 1) ~ score, data = ebmt1)
##
##                     coef exp(coef) se(coef)     z        p
## scoreMedium risk 0.4775    1.6120   0.1412 3.382  0.00072
## scoreHigh risk   1.1410    3.1298   0.1948 5.856 4.73e-09
##
## Likelihood ratio test=32.52  on 2 df, p=8.676e-08
## n= 1835, number of events= 421
```

```
## Call:
## coxph(formula = Surv(rfs, rfsstat == 2) ~ score, data = ebmt1)
##
##                     coef exp(coef) se(coef)     z        p
## scoreMedium risk 0.6937    2.0012   0.1287 5.392 6.98e-08
## scoreHigh risk   1.2539    3.5038   0.1649 7.604 2.86e-14
##
## Likelihood ratio test=61.2  on 2 df, p=5.131e-14
## n= 1835, number of events= 641
```

Now we repeat the same thing with risk groups + `age10`.

```
## Call:
## coxph(formula = Surv(rfs, rfsstat == 1) ~ score + age10, data = ebmt1)
##
##                       coef exp(coef) se(coef)     z        p
## scoreMedium risk 0.475958  1.609556 0.149170 3.191  0.00142
## scoreHigh risk   1.139020  3.123705 0.204680 5.565 2.62e-08
## age10            0.001663  1.001665 0.053184 0.031  0.97505
##
## Likelihood ratio test=32.52  on 3 df, p=4.064e-07
## n= 1835, number of events= 421
```

```
## Call:
## coxph(formula = Surv(rfs, rfsstat == 2) ~ score + age10, data = ebmt1)
##
##                      coef exp(coef) se(coef)     z        p
## scoreMedium risk 0.65782   1.93057  0.13438 4.895 9.82e-07
## scoreHigh risk   1.20582   3.33948  0.17279 6.979 2.98e-12
## age10            0.03988   1.04069  0.04256 0.937    0.349
##
## Likelihood ratio test=62.08  on 3 df, p=2.11e-13
## n= 1835, number of events= 641
```

Note that there is some evidence of non-proportional hazards for the EBMT risk score on relapse (first output), not on non-relapse mortality (second output).

```
##                       rho chisq       p
```

```
## scoreMedium risk -0.1283  7.13 0.00756
## scoreHigh risk   -0.1110  5.25 0.02196
## age10             0.0302  0.43 0.51195
## GLOBAL                NA  7.77 0.05107
```

```
##                      rho  chisq     p
## scoreMedium risk  0.04721 1.4669 0.226
## scoreHigh risk    0.03507 0.7973 0.372
## age10            -0.00685 0.0299 0.863
## GLOBAL                 NA 1.5053 0.681
```

2.2 Cumulative incidences

Non-parametric estimates of the cumulative incidences of relapse and non-relapse mortality for each of the risk scores are shown below.

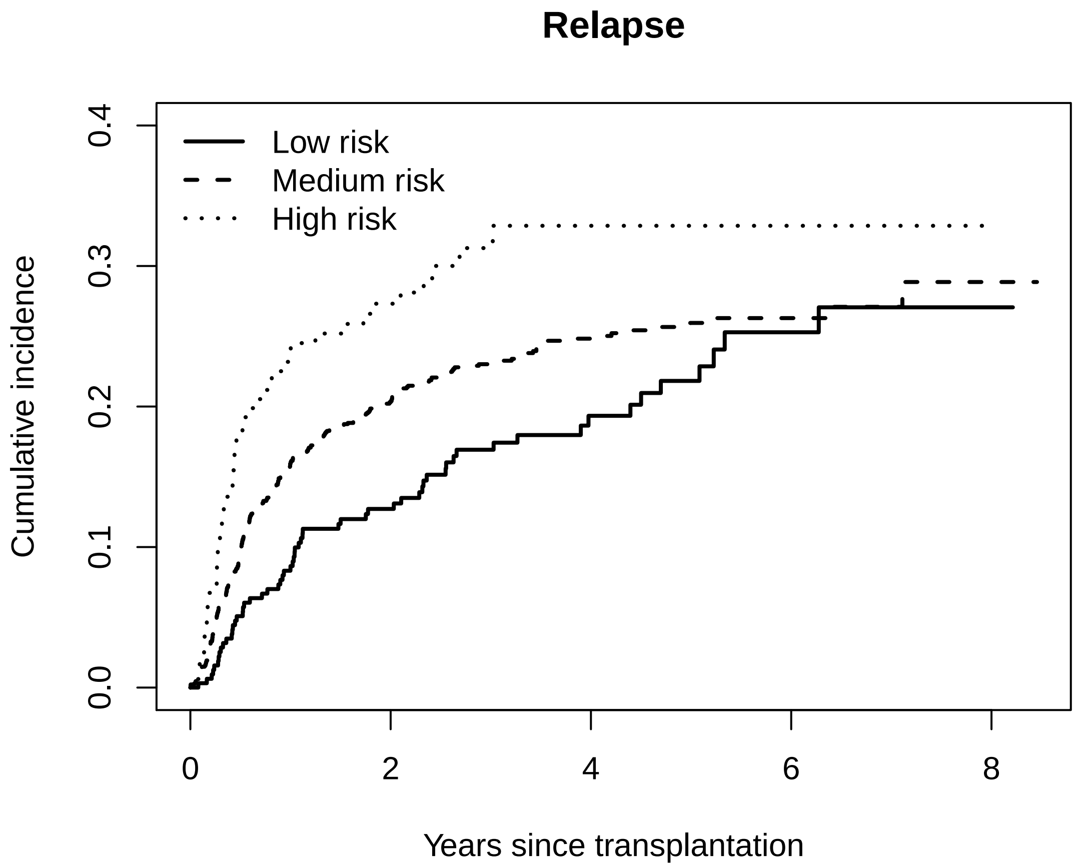
Relapse
Low risk
Medium risk
High risk
Cumulative incidence
0.0
0.1
0.2
0.3
0.4
0
2
4
6
8
Years since transplantation

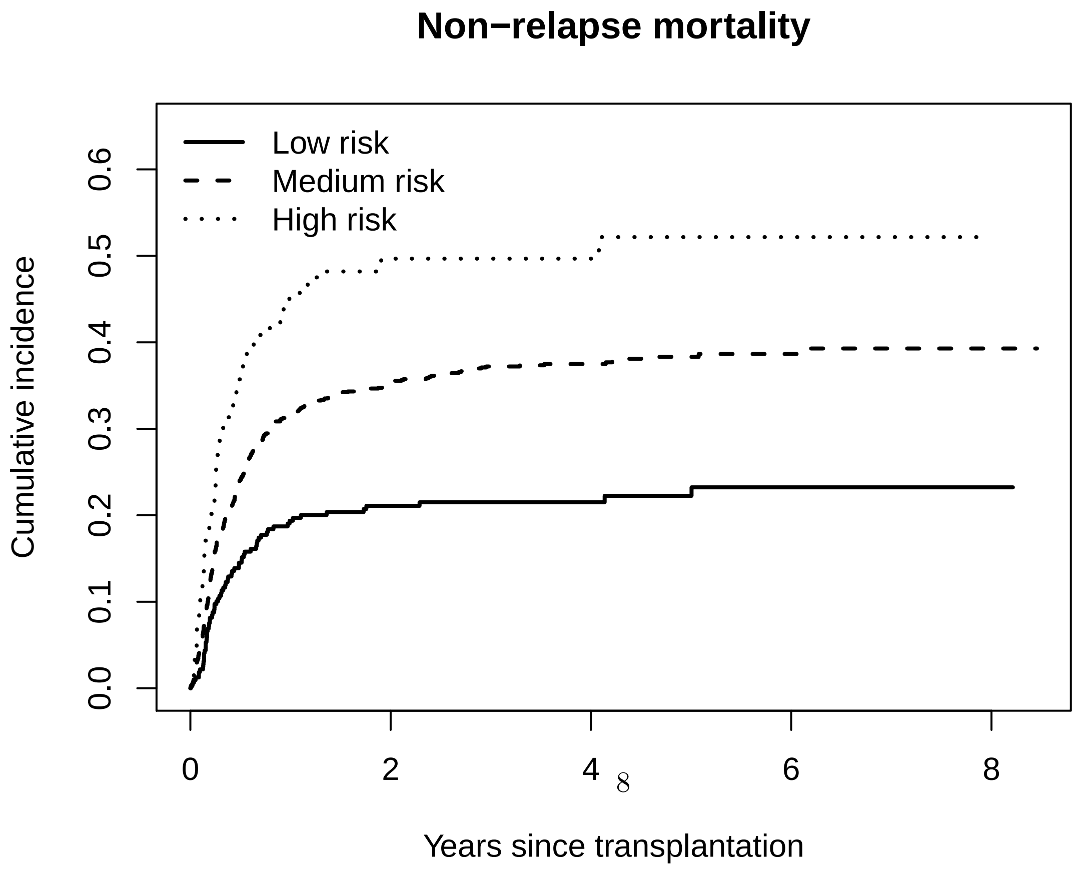
Non−relapse mortality
Low risk
Medium risk
High risk
Cumulative incidence
0.0
0.1
0.2
0.3
0.4
0.5
0.6
0
2
4
6
8
Years since transplantation

The Fine-Gray model can be fitted with the `cmprsk` package. Results are shown first with risk groups only (cause 1, then cause 2).

```
## convergence:  TRUE
## coefficients:
## scoreMedium risk   scoreHigh risk
##           0.2768           0.6046
## standard errors:
## [1] 0.1371 0.1940
## two-sided p-values:
## scoreMedium risk   scoreHigh risk
##           0.0440           0.0018
```

```
## convergence:  TRUE
## coefficients:
## scoreMedium risk   scoreHigh risk
##           0.6201           1.0530
## standard errors:
## [1] 0.1290 0.1663
## two-sided p-values:
## scoreMedium risk   scoreHigh risk
##          1.5e-06          2.5e-10
```

Then with risk groups and age (again cause 1, then cause 2).

```
## convergence:  TRUE
## coefficients:
## scoreMedium risk   scoreHigh risk            age10
##           0.2873           0.6193          -0.0120
## standard errors:
## [1] 0.14730 0.20820 0.05597
## two-sided p-values:
## scoreMedium risk   scoreHigh risk            age10
##           0.0510           0.0029           0.8300
```

```
## convergence:  TRUE
## coefficients:
## scoreMedium risk   scoreHigh risk            age10
##          0.58370          1.00400          0.04109
## standard errors:
## [1] 0.13530 0.17390 0.04195
## two-sided p-values:
## scoreMedium risk   scoreHigh risk            age10
##          1.6e-05          7.8e-09          3.3e-01
```

2.3 Estimation of $r(t)$

We will now estimate the reduction factor non-parametrically for each of the risk scores. That is done using inverse probability of censoring weighting. For that we will need an estimate of the

censoring distribution. Below is the reverse Kaplan-Meier estimate.

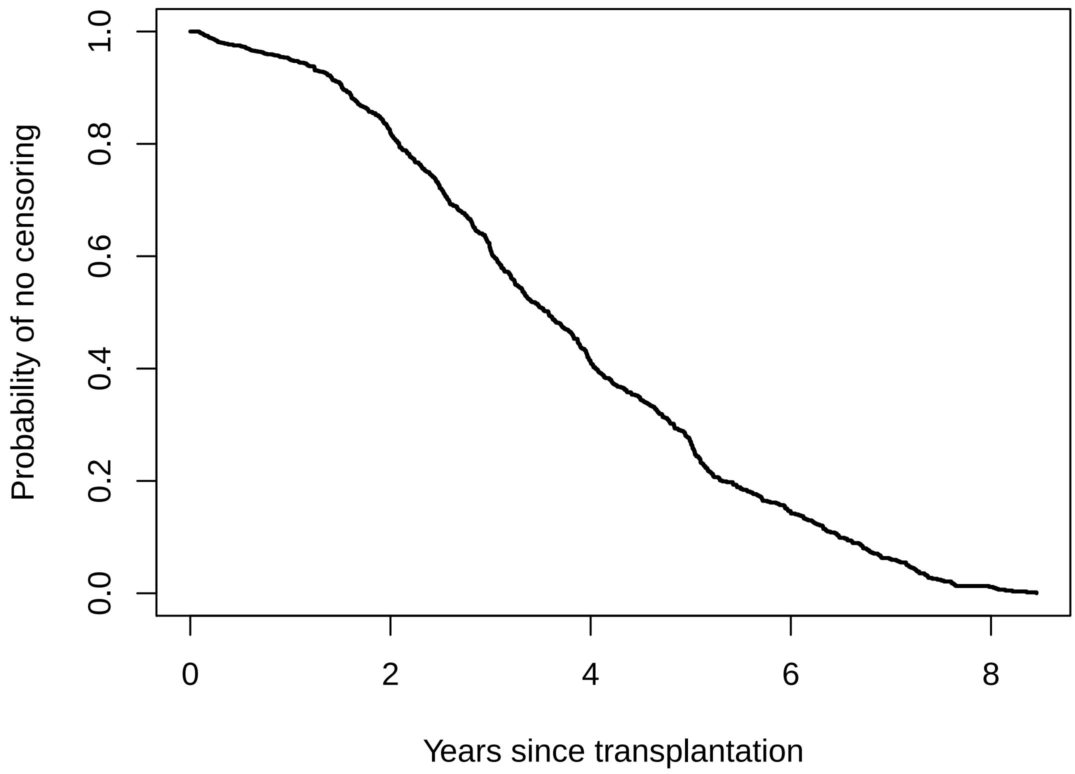

The non-parametric estimates of the reduction factor at 1,...,7 years for the three risk groups are given below (only cause 1).

```
## [1] 0.7870950 0.7573849 0.7428123 0.7481294 0.7491554 0.7414377 0.7631364
```

```
## [1] 0.6230536 0.5627804 0.5246694 0.5011233 0.4704780 0.4496149 0.4215385
```

```
## [1] 0.3950967 0.3117461 0.2001194 0.1709540 0.1809585 0.2314265 0.1773066
```

Plots for both causes are shown below.

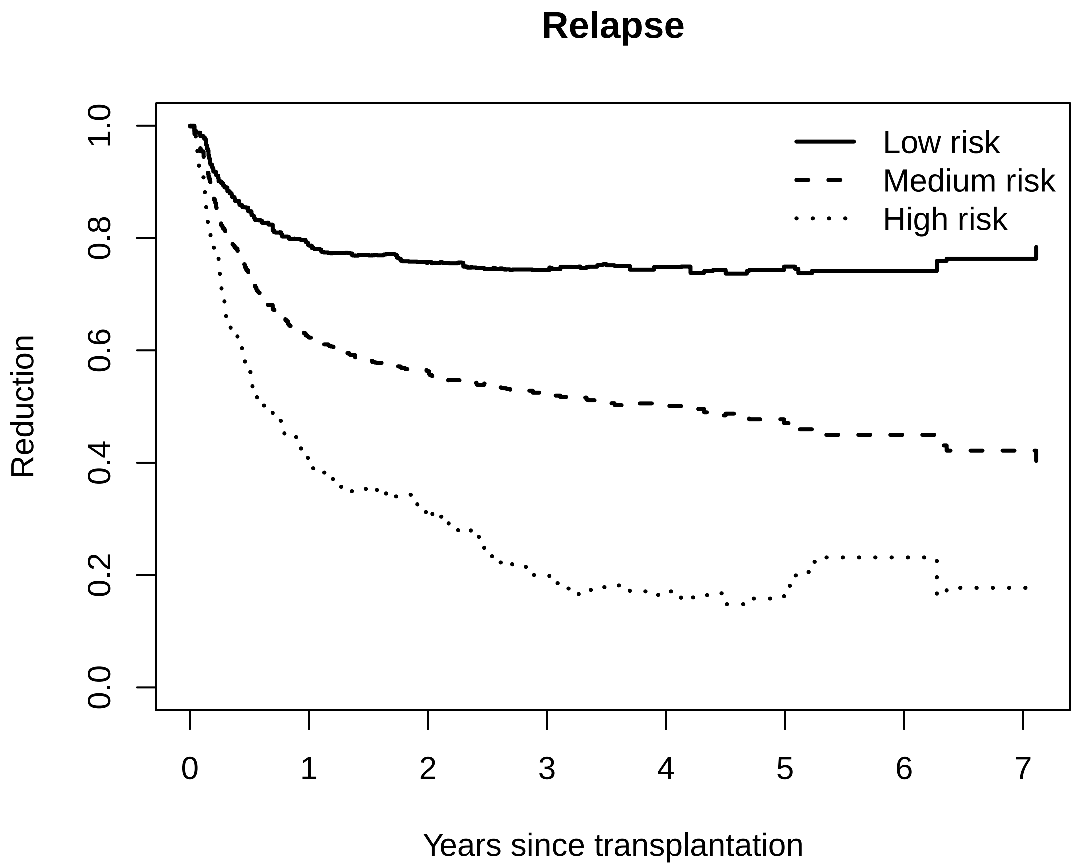

Relapse
Low risk
Medium risk
High risk
Reduction
Years since transplantation
0.0
0.2
0.4
0.6
0.8
1.0
0
1
2
3
4
5
6
7

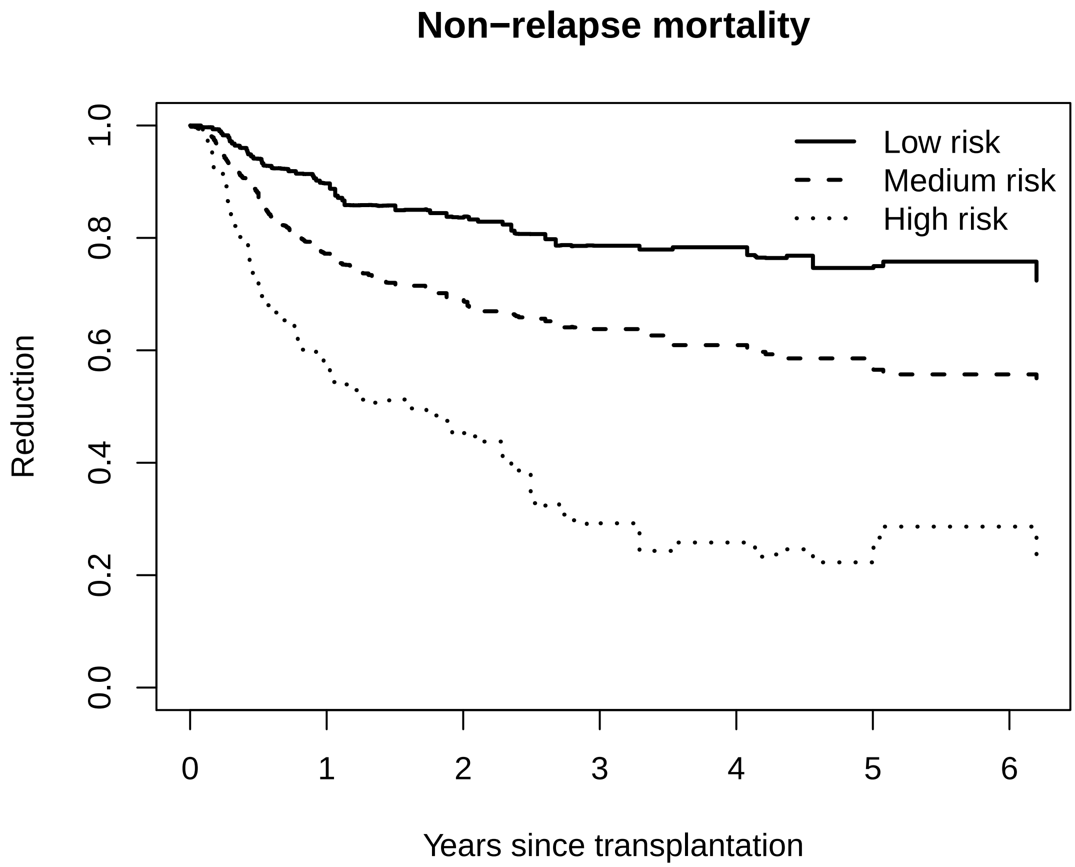

Here are the plots for relapse again, this time showing transformtation of the reduction factor, namely $\log(r(t|x))$ and $\text{cloglog}(r(t|x))$.

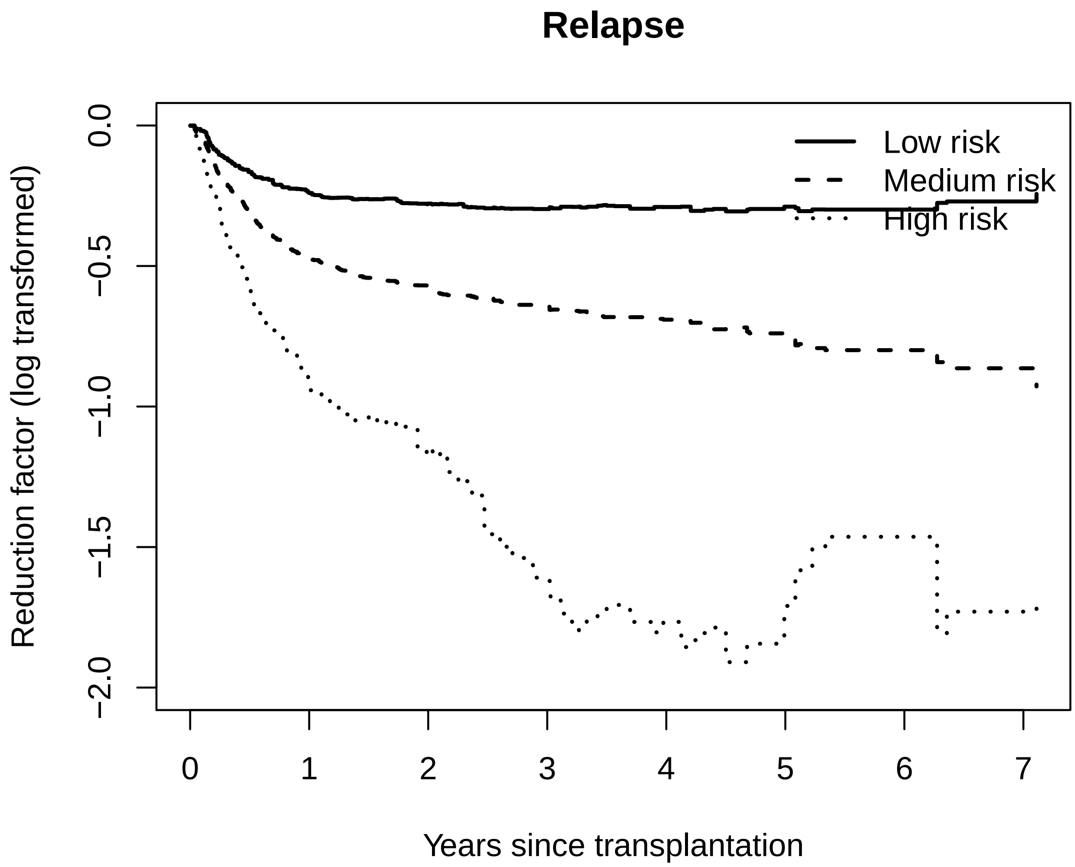
Relapse
Reduction factor (log transformed)
0.0
−0.5
−1.0
−1.5
−2.0
0
1
2
3
4
5
6
7
Years since transplantation
Low risk
Medium risk
High risk

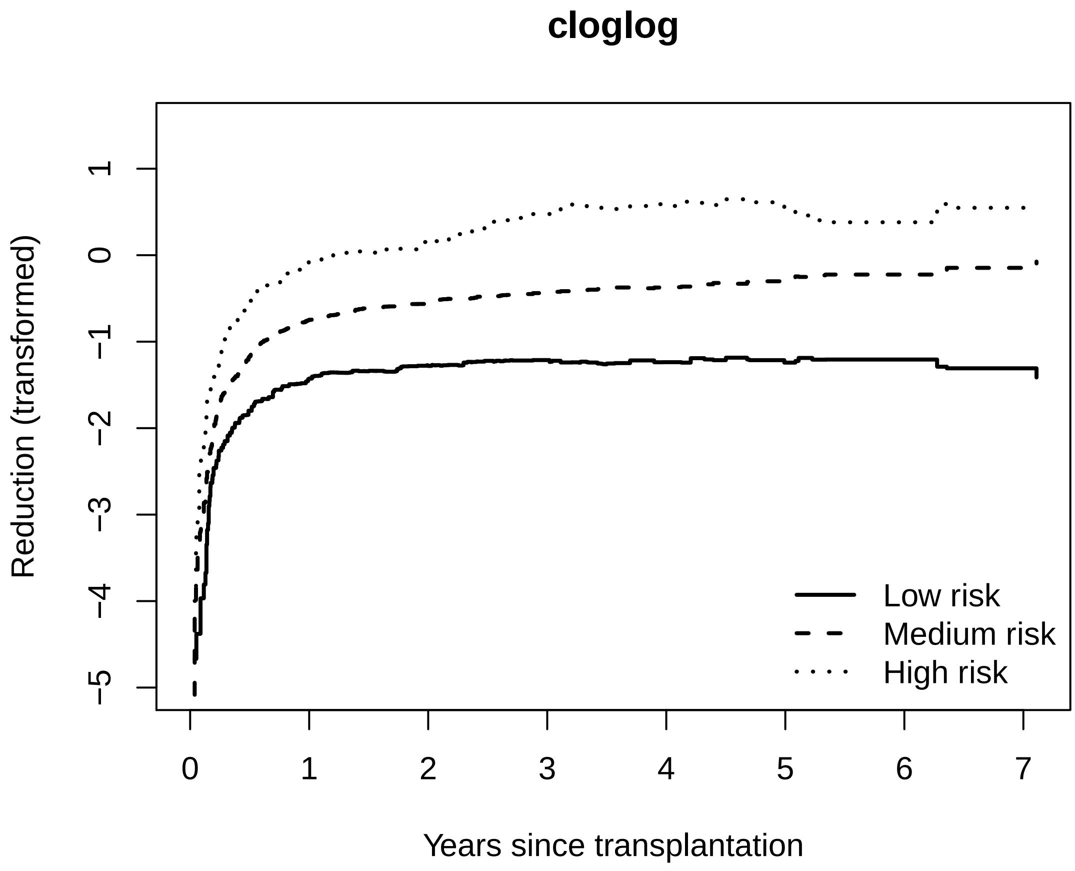

And also the plots for NRM again, showing $\log(r(t|x))$ and $\text{cloglog}(r(t|x))$.

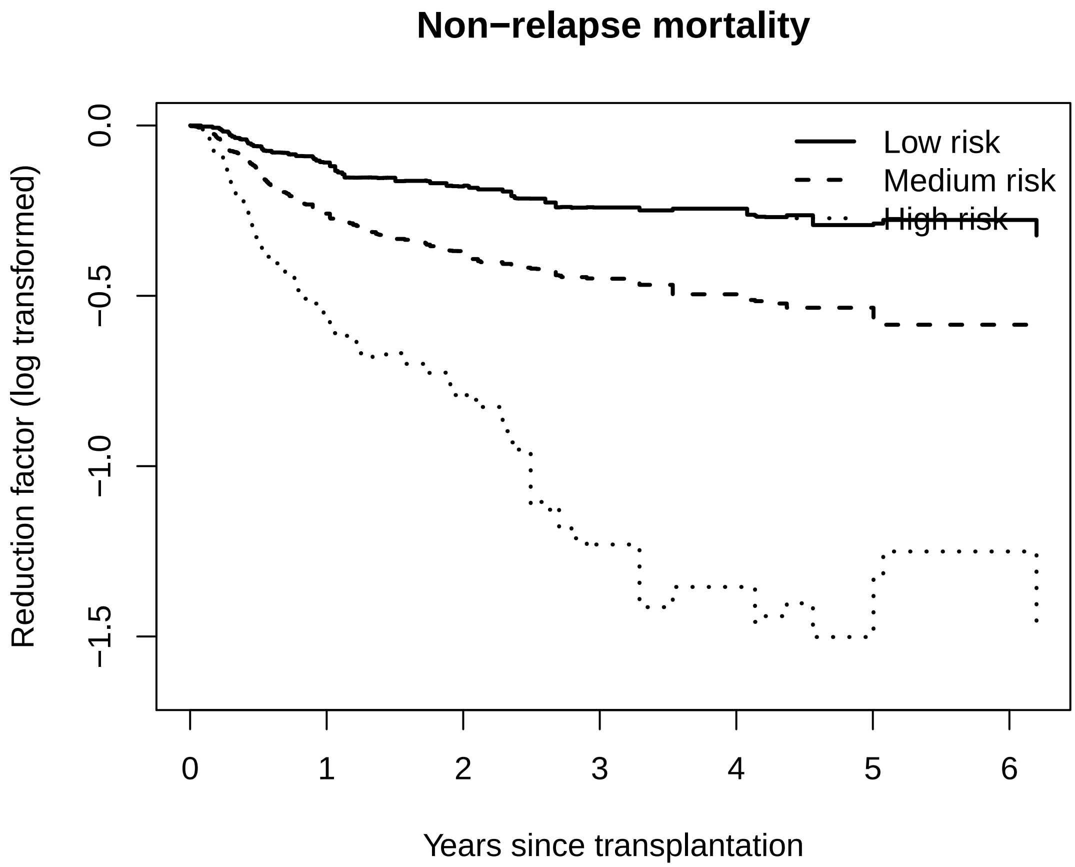
Non−relapse mortality
Low risk
Medium risk
High risk
0.0
−0.5
−1.0
−1.5
Reduction factor (log transformed)
0
1
2
3
4
5
6
Years since transplantation

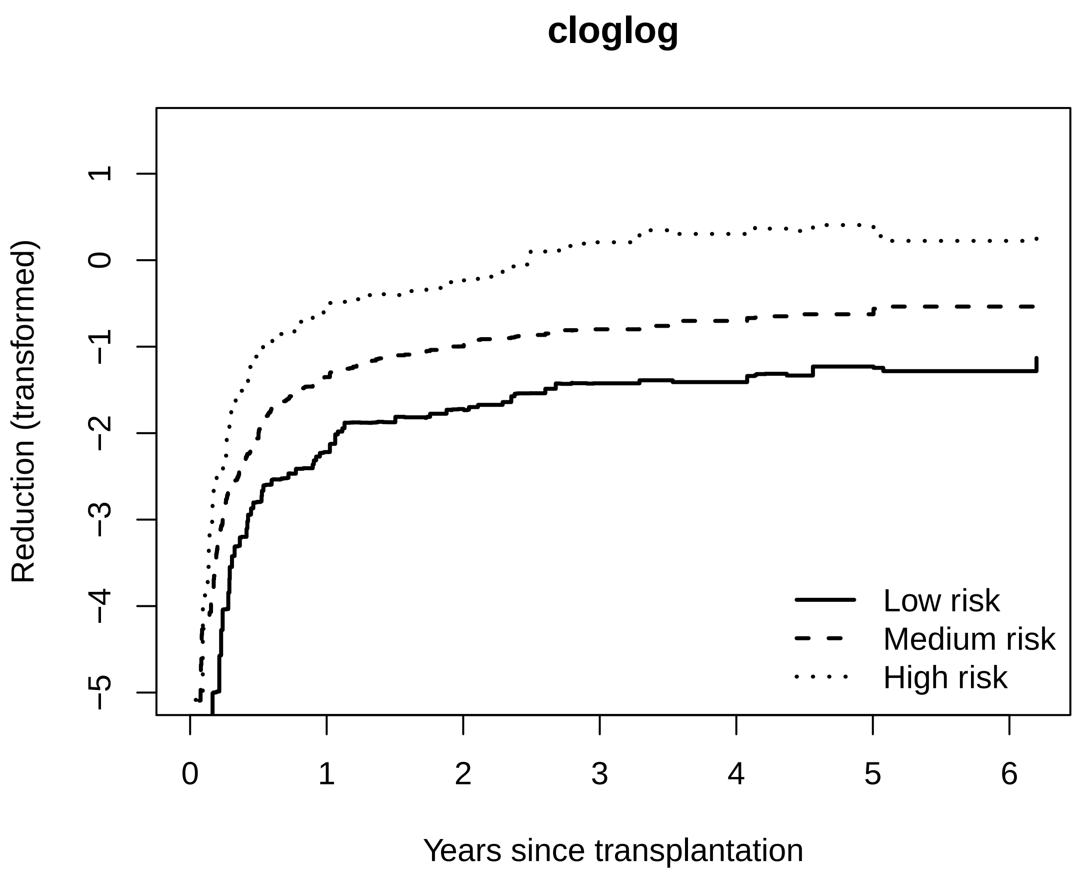

2.3.1 Almost saturated model for $r(t|x)$

The aim here is to quickly fit GLM models for each of the risk sets, with separate intercepts and effects of score and age. If age would not have been there it would have been a completely saturated model, but since age is included as a continuous covariate it is not. But we expect it to be sufficiently rich to capture the behaviour of $r(t|x)$.

2.3.1.1 Relapse

Here is a plot of the coefficients. First the coefficients (contrasts), then the log-transformed and the untransformed reduction factors for `age10`=0 and the three risk groups.

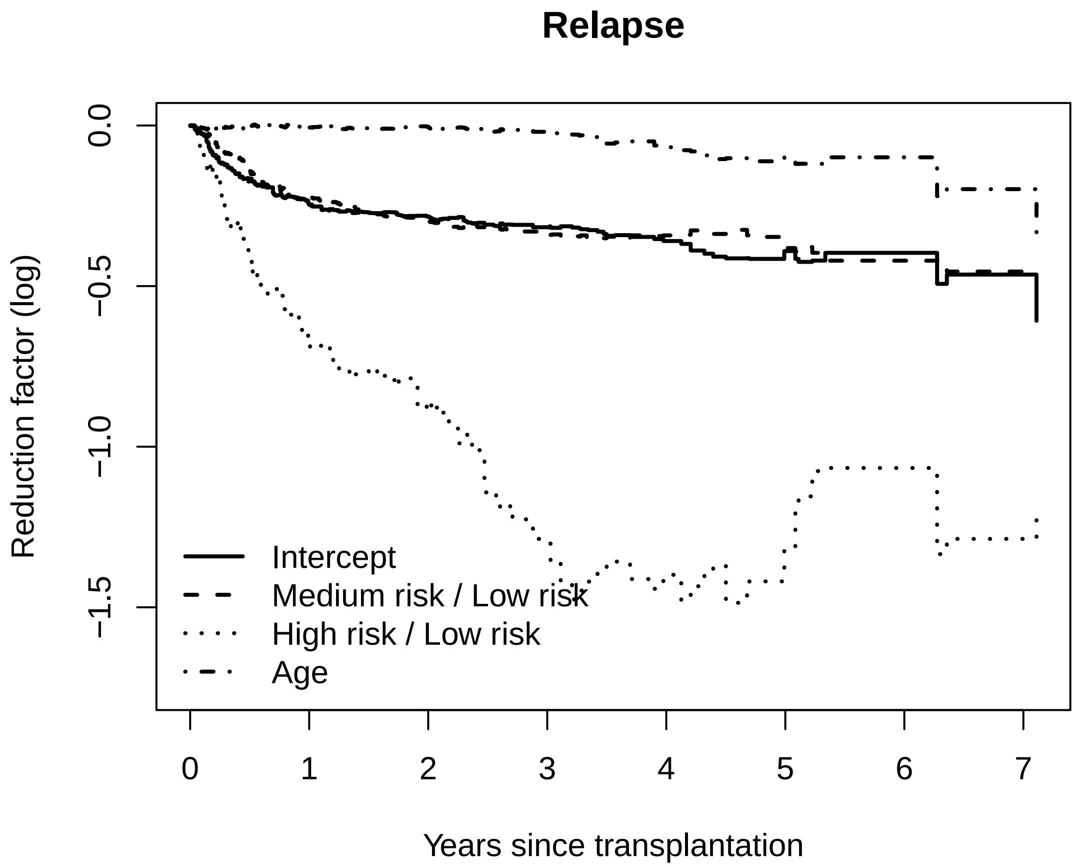

Relapse
Reduction factor (log)
0.0
−0.5
−1.0
−1.5
Intercept
Medium risk / Low risk
High risk / Low risk
Age
0
1
2
3
4
5
6
7
Years since transplantation

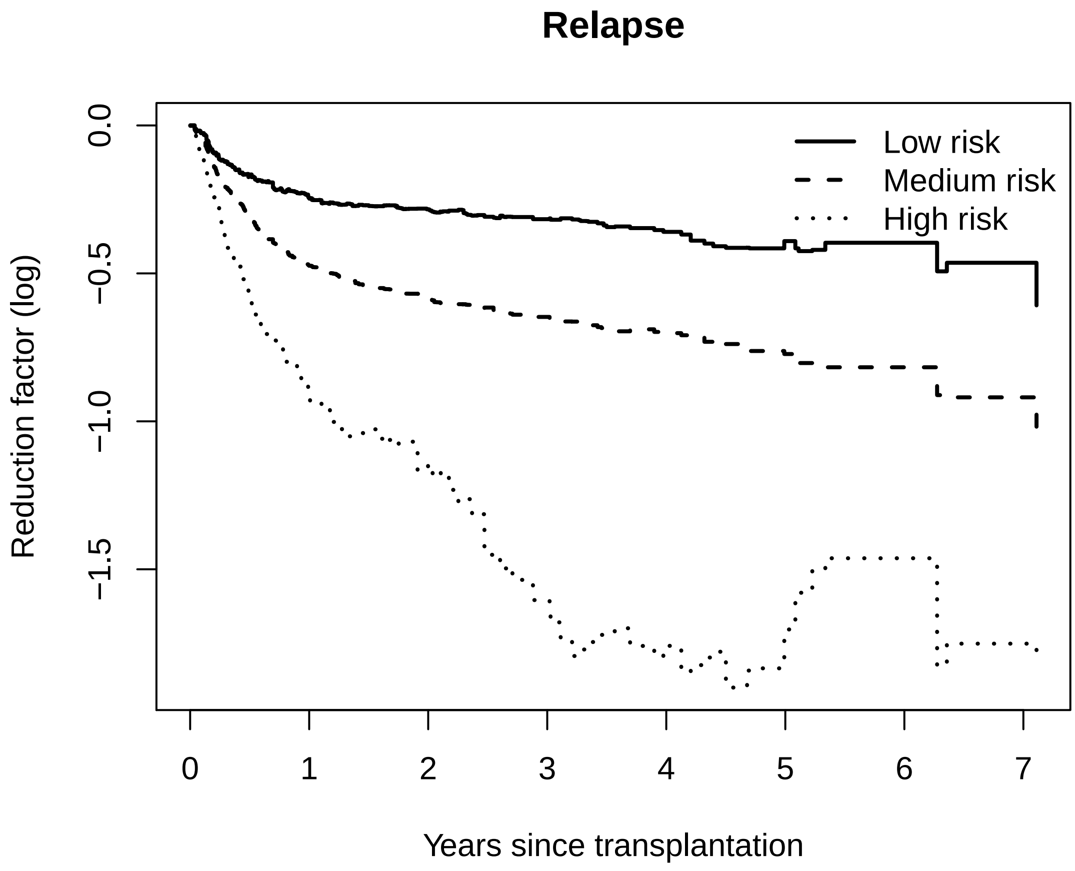

Relapse
Low risk
Medium risk
High risk
0.0
−0.5
−1.0
−1.5
Reduction factor (log)
0
1
2
3
4
5
6
7
Years since transplantation

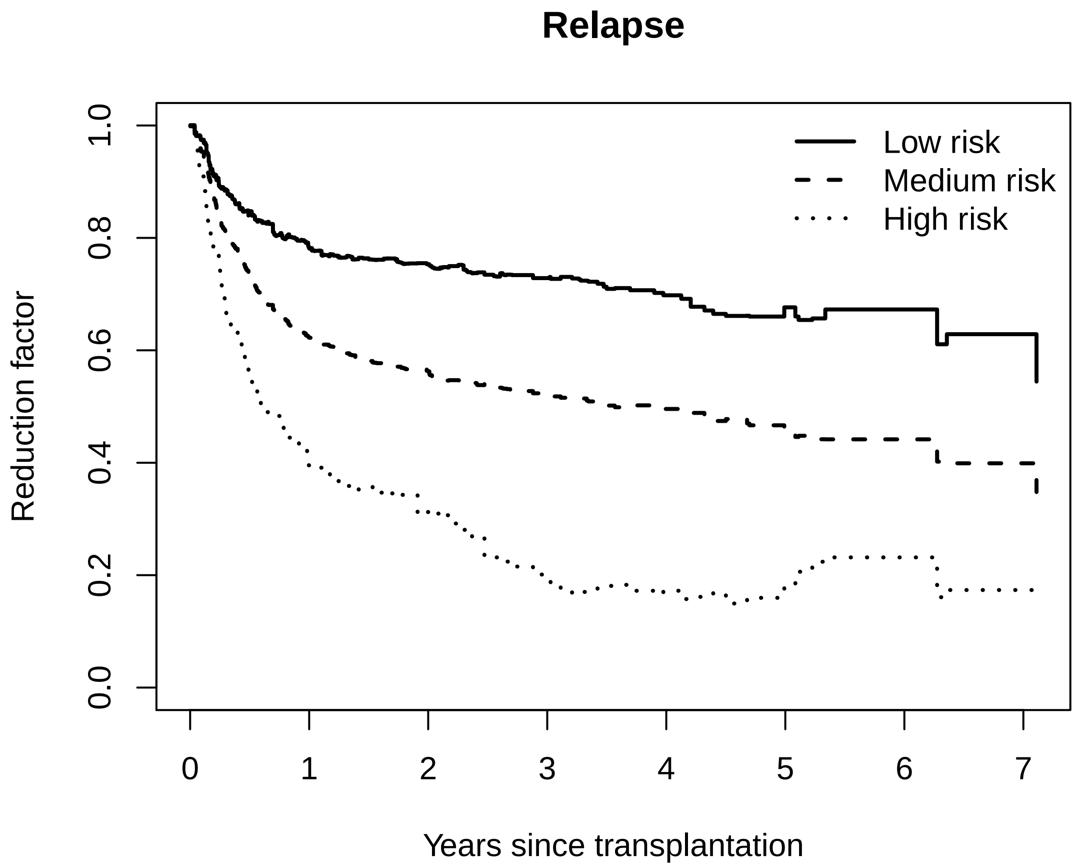

2.3.1.2 Non-relapse mortality

We now repeat estimation of $r(t|x)$ for NRM.

Here is a plot of the coefficients. First the coefficients (contrasts), then the log-transformed and the untransformed reduction factors for `age10`=0 and the three risk groups.

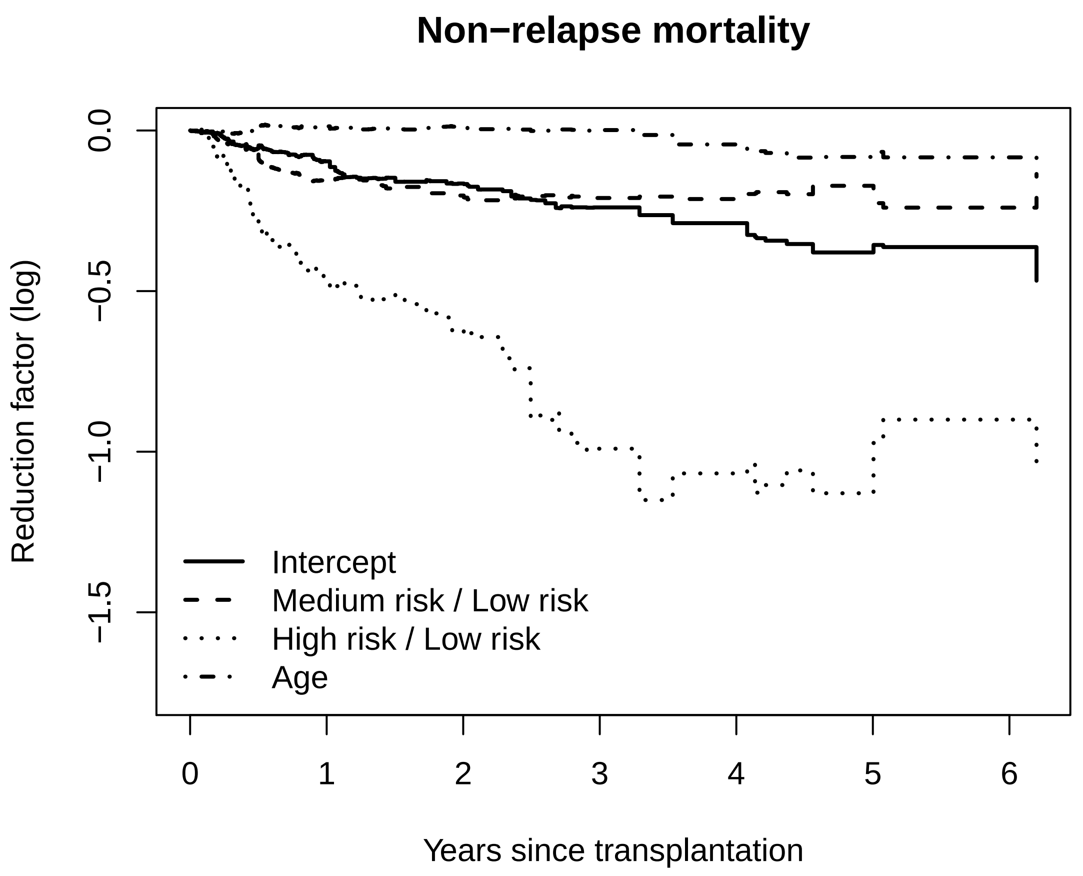

Non−relapse mortality
Reduction factor (log)
0.0
−0.5
−1.0
−1.5
Intercept
Medium risk / Low risk
High risk / Low risk
Age
0
1
2
3
4
5
6
Years since transplantation

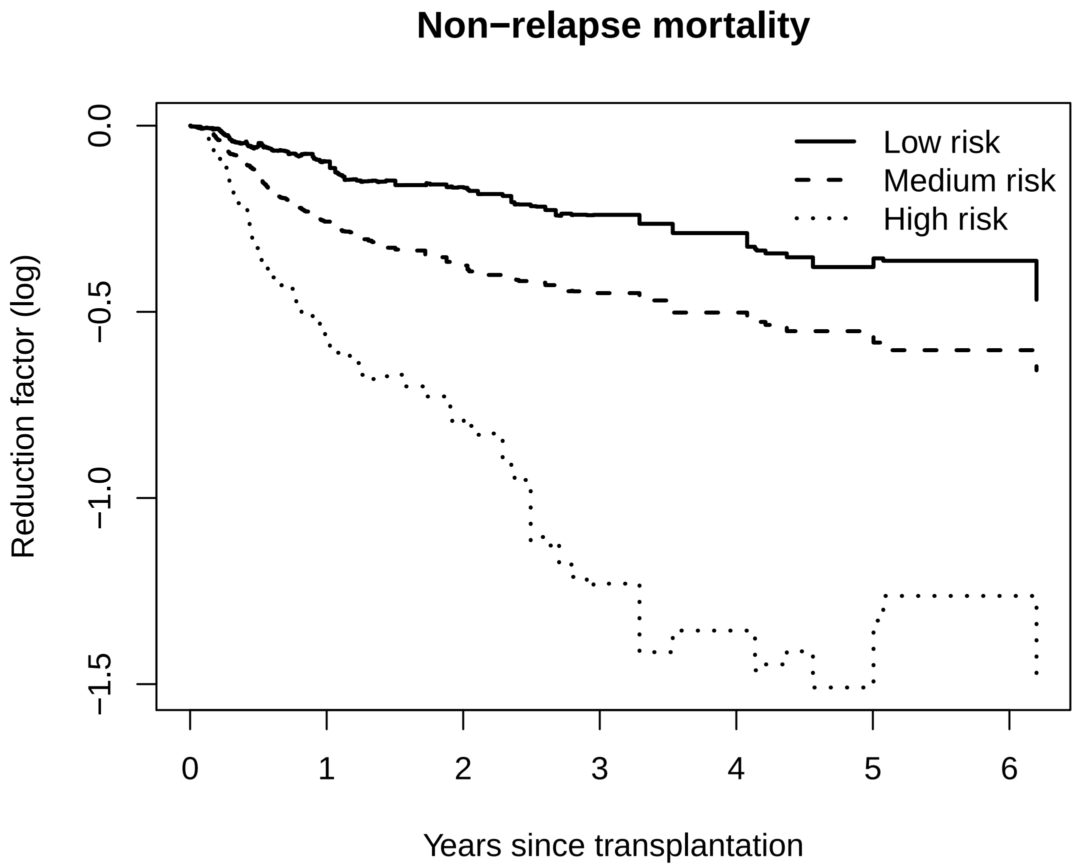
Non−relapse mortality
Reduction factor (log)
0.0
−0.5
−1.0
−1.5
0
1
2
3
4
5
6
Years since transplantation
Low risk
Medium risk
High risk

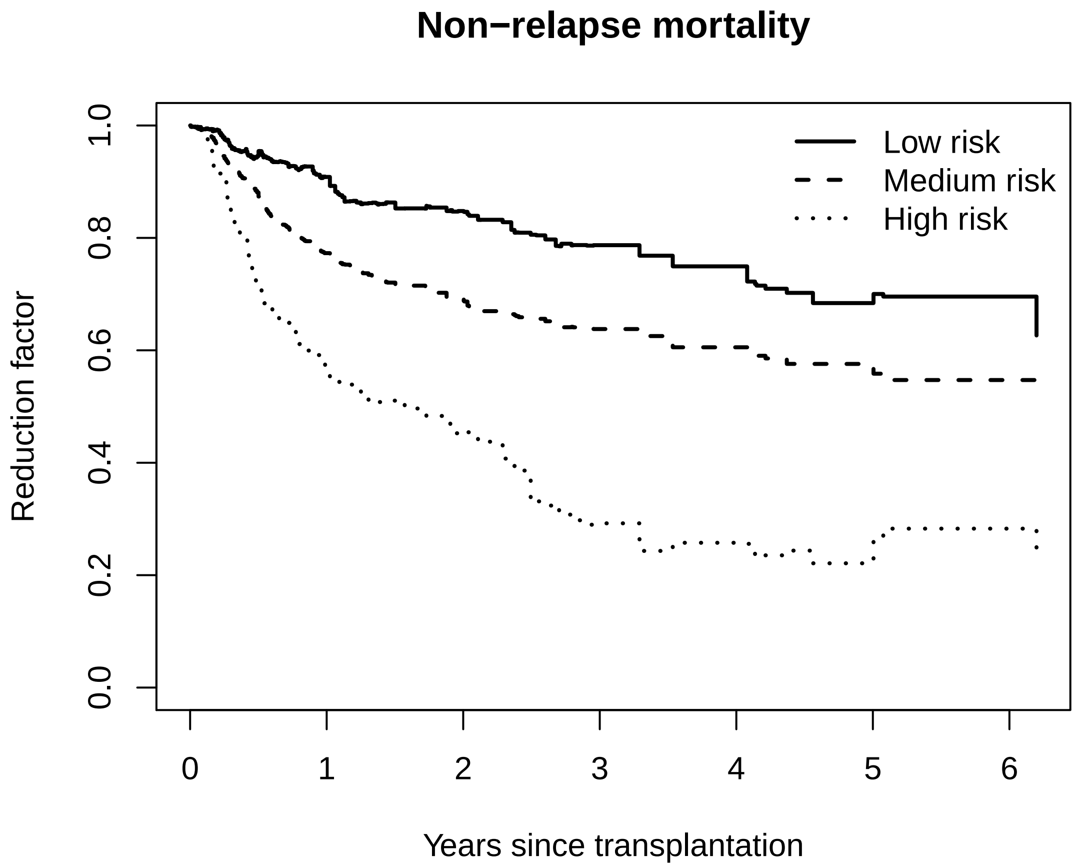

2.4 Retrieving Fine-Gray with cause-specific hazards and r(t|x) offset

Using non-parametric estimates of $r(t|x)$ as offset (logarithmic) in model for cause-specific hazards (risk groups only), gives the following. For cause 1 (relapse):

```
## $par
## [1] 0.2771856 0.6045621
##
## $value
## [1] 2889.611
##
## $counts
## function gradient
##       43       NA
##
## $convergence
## [1] 0
##
## $message
## NULL
```

The estimates look slightly different from the Fine-Gray estimates 0.2767972, 0.6046399 obtained from `cmrpsk`, but they really aren't. The log-likelihoods at the optimum and at the earlier Fine-Gray estimates are given below, as well as their difference.

```
## [1] 2889.611
```

```
## [1] 2889.611
```

```
## [1] -7.253251e-06
```

For cause 2 (NRM):

```
## $par
## [1] 0.619681 1.051235
##
## $value
## [1] 4551.106
##
## $counts
## function gradient
##       47       NA
##
## $convergence
## [1] 0
##
## $message
## NULL
```

Another way of achieving this should be with long format data, and calling `coxph` with $-\log(r(t|x))$ as offset. The advantage would be that it also gives standard error, and it is of interest to see whether these standard errors are the same as obtained by Fine-Gray. First for relapse.

```
## Call:
## coxph(formula = Surv(tstart, time, d1) ~ score + offset(minlogrtx),
##     data = ebmtlong)
##
##   n= 457425, number of events= 421
##
##                    coef exp(coef) se(coef)     z Pr(>|z|)
## scoreMedium risk 0.2768    1.3189   0.1410 1.964   0.0496 *
## scoreHigh risk   0.6046    1.8306   0.1937 3.122   0.0018 **
## ---
## Signif. codes:  0 '***' 0.001 '**' 0.01 '*' 0.05 '.' 0.1 ' ' 1
##
##                  exp(coef) exp(-coef) lower .95 upper .95
## scoreMedium risk     1.319     0.7582     1.001     1.739
## scoreHigh risk       1.831     0.5463     1.252     2.676
##
## Concordance= 0.57  (se = 0.011 )
## Likelihood ratio test= 9.61  on 2 df,   p=0.008
## Wald test            = 9.75  on 2 df,   p=0.008
```

```
## Score (logrank) test = 9.88  on 2 df,   p=0.007
```

One advantage of this approach is that `cox.zph` can be called to test for proportional hazards (on the subdistribution scale!).

Actually, this gives an error `Error in residuals.coxph(fit, "schoenfeld") : NA/NaN/Inf in foreign function call (arg 5)`, not sure whether this is because of the long data structure, because of the offset, or because of something else.

Terry Therneau pointed out that the latest version of the `survival` package includes an implementation of the Fine-Gray method, using weighted Cox regression. Here is the result (including test for the proportional hazards assumption on the subdistribution rates for relapse). It is identical (including standard errors) to the $\log(r(t|x))$ offset with long format data.

```
## [1] 79045    21
```

```
## Call:
## coxph(formula = Surv(fgstart, fgstop, fgstatus) ~ score, data = ebmtdat,
##     weights = fgwt)
##
##   n= 79045, number of events= 421
##
##                     coef exp(coef) se(coef)     z Pr(>|z|)
## scoreMedium risk 0.2768    1.3189   0.1410 1.964   0.0496 *
## scoreHigh risk   0.6046    1.8306   0.1937 3.122   0.0018 **
## ---
## Signif. codes:  0 '***' 0.001 '**' 0.01 '*' 0.05 '.' 0.1 ' ' 1
##
##                  exp(coef) exp(-coef) lower .95 upper .95
## scoreMedium risk     1.319     0.7582     1.001     1.739
## scoreHigh risk       1.831     0.5463     1.252     2.676
##
## Concordance= 0.544  (se = 0.01 )
## Likelihood ratio test= 9.61  on 2 df,   p=0.008
## Wald test            = 9.75  on 2 df,   p=0.008
## Score (logrank) test = 9.88  on 2 df,   p=0.007
```

And this approach *does* provide a test of the proportional hazards assumption (on the subdistribution rate).

```
##                     rho chisq        p
## scoreMedium risk -0.16  10.7 0.001063
## scoreHigh risk   -0.19  15.0 0.000106
## GLOBAL              NA  16.3 0.000284
```

Here is the same approach with EBMT risk score and age (by 10).

```
## Call:
## coxph(formula = Surv(fgstart, fgstop, fgstatus) ~ score + age10,
##     data = ebmtdat, weights = fgwt)
##
##   n= 79045, number of events= 421
```

```
##
##                      coef exp(coef) se(coef)      z Pr(>|z|)
## scoreMedium risk  0.2873    1.3329   0.1485  1.936  0.05293 .
## scoreHigh risk    0.6193    1.8576   0.2042  3.032  0.00243 **
## age10            -0.0120    0.9881   0.0532 -0.225  0.82162
## ---
## Signif. codes:  0 '***' 0.001 '**' 0.01 '*' 0.05 '.' 0.1 ' ' 1
##
##                  exp(coef) exp(-coef) lower .95 upper .95
## scoreMedium risk    1.3329     0.7503    0.9964     1.783
## scoreHigh risk      1.8576     0.5383    1.2448     2.772
## age10               0.9881     1.0121    0.8902     1.097
##
## Concordance= 0.55  (se = 0.014 )
## Likelihood ratio test= 9.66  on 3 df,   p=0.02
## Wald test            = 9.8  on 3 df,   p=0.02
## Score (logrank) test = 9.93  on 3 df,   p=0.02
```

And a test for the proportional hazards assumption.

```
##                      rho  chisq        p
## scoreMedium risk -0.1534 10.202 0.001403
## scoreHigh risk   -0.1805 14.163 0.000168
## age10             0.0128  0.077 0.781471
## GLOBAL                NA 16.403 0.000938
```

Then for non-relapse mortality.

```
## Call:
## coxph(formula = Surv(tstart, time, d2) ~ score + offset(minlogrtx),
##     data = ebmtlong)
##
##   n= 457425, number of events= 641
##
##                     coef exp(coef) se(coef)     z Pr(>|z|)
## scoreMedium risk 0.6197    1.8583   0.1286 4.819 1.44e-06 ***
## scoreHigh risk   1.0518    2.8627   0.1644 6.398 1.58e-10 ***
## ---
## Signif. codes:  0 '***' 0.001 '**' 0.01 '*' 0.05 '.' 0.1 ' ' 1
##
##                  exp(coef) exp(-coef) lower .95 upper .95
## scoreMedium risk     1.858     0.5381     1.444     2.391
## scoreHigh risk       2.863     0.3493     2.074     3.951
##
## Concordance= 0.275  (se = 0.011 )
## Likelihood ratio test= 44.41  on 2 df,   p=2e-10
## Wald test            = 41.28  on 2 df,   p=1e-09
## Score (logrank) test = 43.02  on 2 df,   p=5e-10
```

Here is the `cox.zph` test for proportional hazards on the subdistribution scale for non-relapse mortality.

This gives the same error `Error in residuals.coxph(fit, "schoenfeld") : NA/NaN/Inf in foreign function call (arg 5)`, unfortunately.

Here is the same thing again, using `finegray` from the `survival` package, for NRM. This time we do not get exactly the same result as the long format with $\log(r(t|x))$ as offset, but it is very close indeed.

```
## [1] 29731    21
```

```
## Call:
## coxph(formula = Surv(fgstart, fgstop, fgstatus) ~ score, data = ebmtdat,
##     weights = fgwt)
##
##   n= 29731, number of events= 641
##
##                     coef exp(coef) se(coef)     z Pr(>|z|)
## scoreMedium risk 0.6201    1.8590   0.1286 4.822 1.42e-06 ***
## scoreHigh risk   1.0525    2.8649   0.1644 6.402 1.53e-10 ***
## ---
## Signif. codes:  0 '***' 0.001 '**' 0.01 '*' 0.05 '.' 0.1 ' ' 1
##
##                  exp(coef) exp(-coef) lower .95 upper .95
## scoreMedium risk     1.859     0.5379     1.445     2.392
## scoreHigh risk       2.865     0.3491     2.076     3.954
##
## Concordance= 0.558  (se = 0.009 )
## Likelihood ratio test= 44.47  on 2 df,   p=2e-10
## Wald test            = 41.33  on 2 df,   p=1e-09
## Score (logrank) test = 43.08  on 2 df,   p=4e-10
```

```
##                      rho  chisq     p
## scoreMedium risk  0.0279 0.5000 0.479
## scoreHigh risk   -0.0121 0.0942 0.759
## GLOBAL                NA 1.6866 0.430
```

```
## Call:
## coxph(formula = Surv(fgstart, fgstop, fgstatus) ~ score + age10,
##     data = ebmtdat, weights = fgwt)
##
##   n= 29731, number of events= 641
##
##                      coef exp(coef) se(coef)     z Pr(>|z|)
## scoreMedium risk 0.58371   1.79268  0.13399 4.356 1.32e-05 ***
## scoreHigh risk   1.00399   2.72916  0.17188 5.841 5.18e-09 ***
## age10            0.04109   1.04195  0.04207 0.977    0.329
## ---
## Signif. codes:  0 '***' 0.001 '**' 0.01 '*' 0.05 '.' 0.1 ' ' 1
##
```

```
##                   exp(coef) exp(-coef) lower .95 upper .95
## scoreMedium risk     1.793     0.5578    1.3786     2.331
## scoreHigh risk       2.729     0.3664    1.9486     3.822
## age10                1.042     0.9597    0.9595     1.132
##
## Concordance= 0.561  (se = 0.011 )
## Likelihood ratio test= 45.43  on 3 df,   p=8e-10
## Wald test            = 42.33  on 3 df,   p=3e-09
## Score (logrank) test = 44.09  on 3 df,   p=1e-09
```

```
##                       rho  chisq     p
## scoreMedium risk  0.02799 0.5116 0.474
## scoreHigh risk   -0.00990 0.0628 0.802
## age10            -0.00583 0.0211 0.884
## GLOBAL                 NA 1.6907 0.639
```

3 Cumulative incidences

Throughout, `score` (risk group, categorical, three groups) and `age10` (continuous) are used as covariates. We will produce estimates of the cumulative incidences of relapse, for age10=0, and low, medium and high risk, using the four different methods outlined in Section 4 in the paper. Then later for NRM. These will be produced, sometimes with intermediate output in each of the following four subsections. Results (estimates of the cumulative incidences of relapse, for age10=0, and low, medium and high risk) are stored and will be shown together for comparison in the final subsection.

3.1 Relapse

3.1.1 Multi-state model approach

Event counts:

```
## $Frequencies
##            to
## from        eventfree Relapse  NRM no event total entering
##   eventfree         0     421  641      773           1835
##   Relapse           0       0    0      421            421
##   NRM               0       0    0      641            641
##
## $Proportions
##            to
## from        eventfree   Relapse       NRM  no event
##   eventfree 0.0000000 0.2294278 0.3493188 0.4212534
##   Relapse   0.0000000 0.0000000 0.0000000 1.0000000
##   NRM       0.0000000 0.0000000 0.0000000 1.0000000
```

The Cox models for the two cause-specific hazards are shown below (`x.1` stands for the effect of covariate `x` on cause 1, `x.2` for the effect on cause 2).

```
## Call:
## coxph(formula = Surv(Tstart, Tstop, status) ~ score1.1 + score2.1 +
##     age10.1 + score1.2 + score2.2 + age10.2 + strata(trans),
##     data = ebmtlong)
##
##               coef exp(coef) se(coef)     z        p
## score1.1 0.475652  1.609063 0.149170 3.189  0.00143
## score2.1 1.137886  3.120165 0.204680 5.559 2.71e-08
## age10.1  0.001677  1.001678 0.053187 0.032  0.97485
## score1.2 0.657663  1.930277 0.134376 4.894 9.87e-07
## score2.2 1.205283  3.337705 0.172782 6.976 3.04e-12
## age10.2  0.039921  1.040728 0.042556 0.938  0.34820
##
## Likelihood ratio test=94.5  on 6 df, p=< 2.2e-16
## n= 3670, number of events= 1062
```

Per risk group (at `age10`=0, so age 40), model-based cumulative incidence curves for relapse and NRM are shown below.

Now a plot with the three risk groups (`age10`=0):

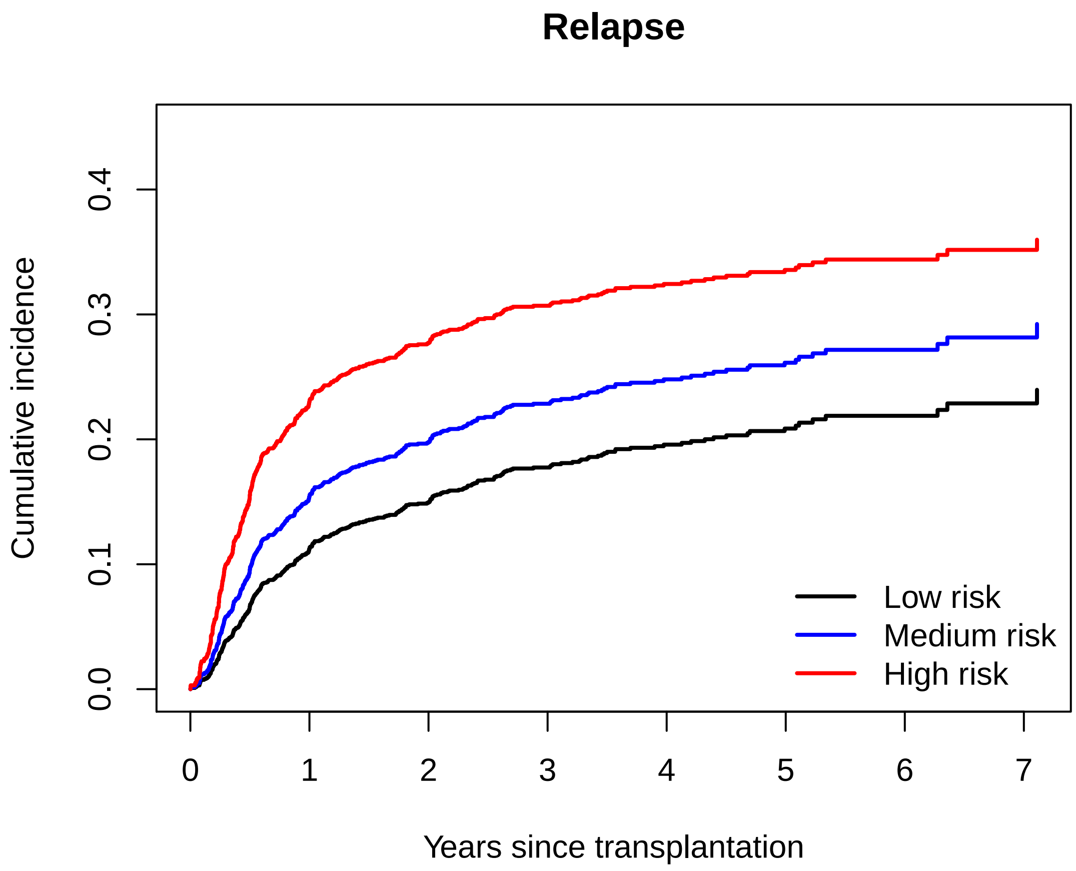

We gather the results (including standard errors) for later use.

3.1.2 Subdistribution rate as product of cause-specific hazard and $r(t|x)$

Using the cause-specific hazards model of the previous subsection, together with the almost saturated GLM of Subsection 2.3.1, we calculate the subdistribution rate for given covariate values x as $\hat{\lambda}(t|x) = \hat{\alpha}_1(t|x)\hat{r}(t|x)$, and from that the cumulative subdistribution hazard $\hat{\Lambda}(t|x) = \sum_{0<s\leq t} \hat{\lambda}(s|x)$, and finally the cumulative incidence function $\hat{F}_1(t|x) = 1 - \exp(-\hat{\Lambda}(t|x))$.

Here we show the relapse cumulative incidence curves for `age10`=0 and the three risk groups.

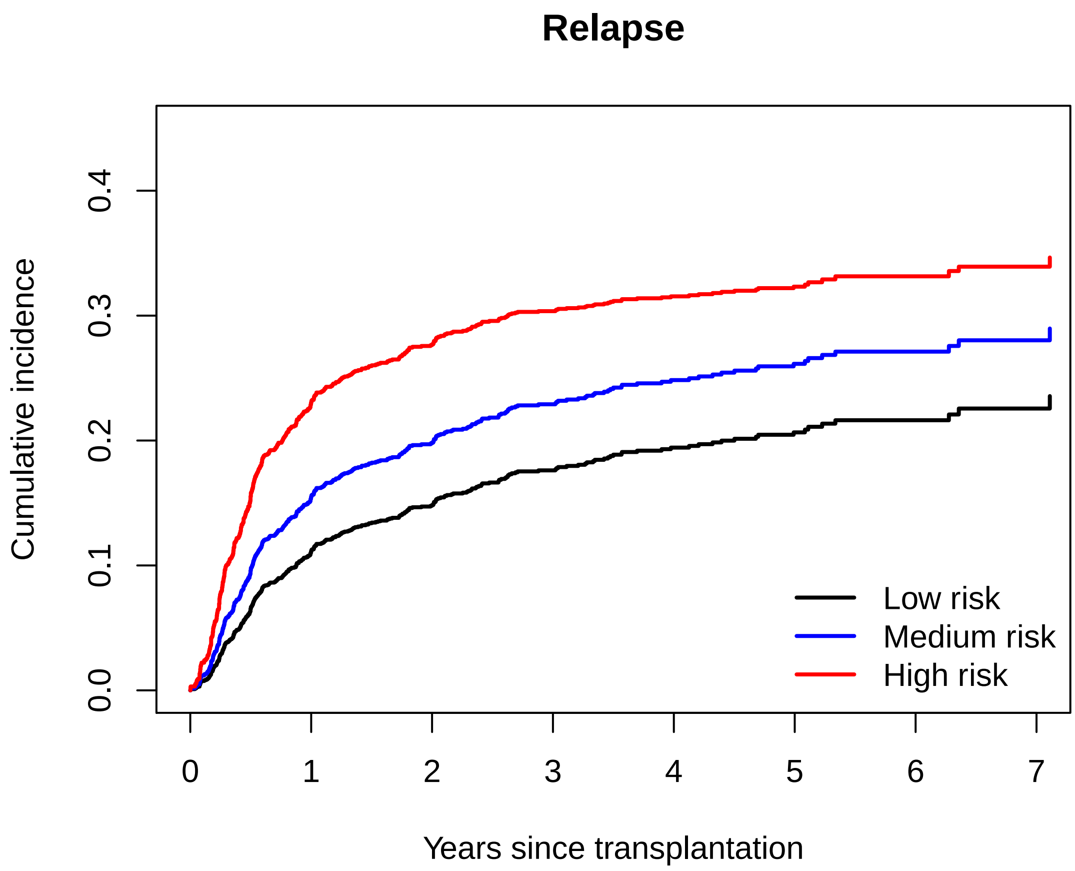

We gather the results in a single data frame.

3.1.3 Fine-Gray

Here, estimates for the cumulative incidences are based on the `predict` function in the `cmprsk` package, using the Fine-Gray results of Section 3.2. First recall the Fine-Gray model of Section 3.2.

```
## convergence:  TRUE
## coefficients:
## scoreMedium risk   scoreHigh risk            age10
##           0.2873           0.6193          -0.0120
## standard errors:
```

```
## [1] 0.14730 0.20820 0.05597
## two-sided p-values:
## scoreMedium risk   scoreHigh risk            age10
##           0.0510           0.0029           0.8300
```

Also here we concentrate on cause 1 (relapse), and we show the relapse cumulative incidence curves for `age10`=0 and the three risk groups.

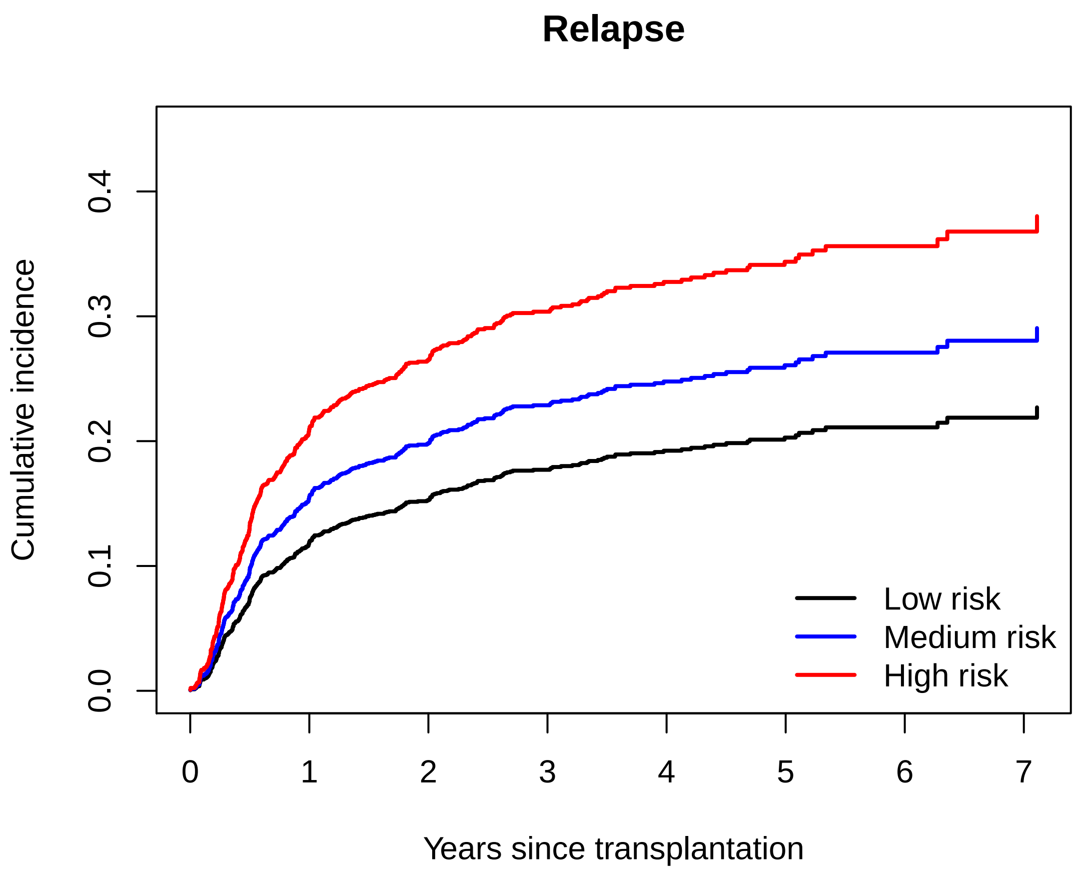

We gather the results in a single data frame.

3.1.4 Fine-Gray through $r(t|x)$

Here we fit a proportional subdistribution rate model, using the usual (cause-specific) log partial likelihood, with -log $\hat{r}(t|x)$ as offset. We no longer expect exactly the same result as in the previous subsection, but probably something quite close.

```
## $par
## [1]  0.28220121  0.61787014 -0.00641563
##
## $value
## [1] 2889.144
```

```
##
## $counts
## function gradient
##       66       NA
##
## $convergence
## [1] 0
##
## $message
## NULL
```

```
## [1] 2889.144
```

The baseline hazard has been saved on the way, and the cumulative incidence curves can now be plotted.

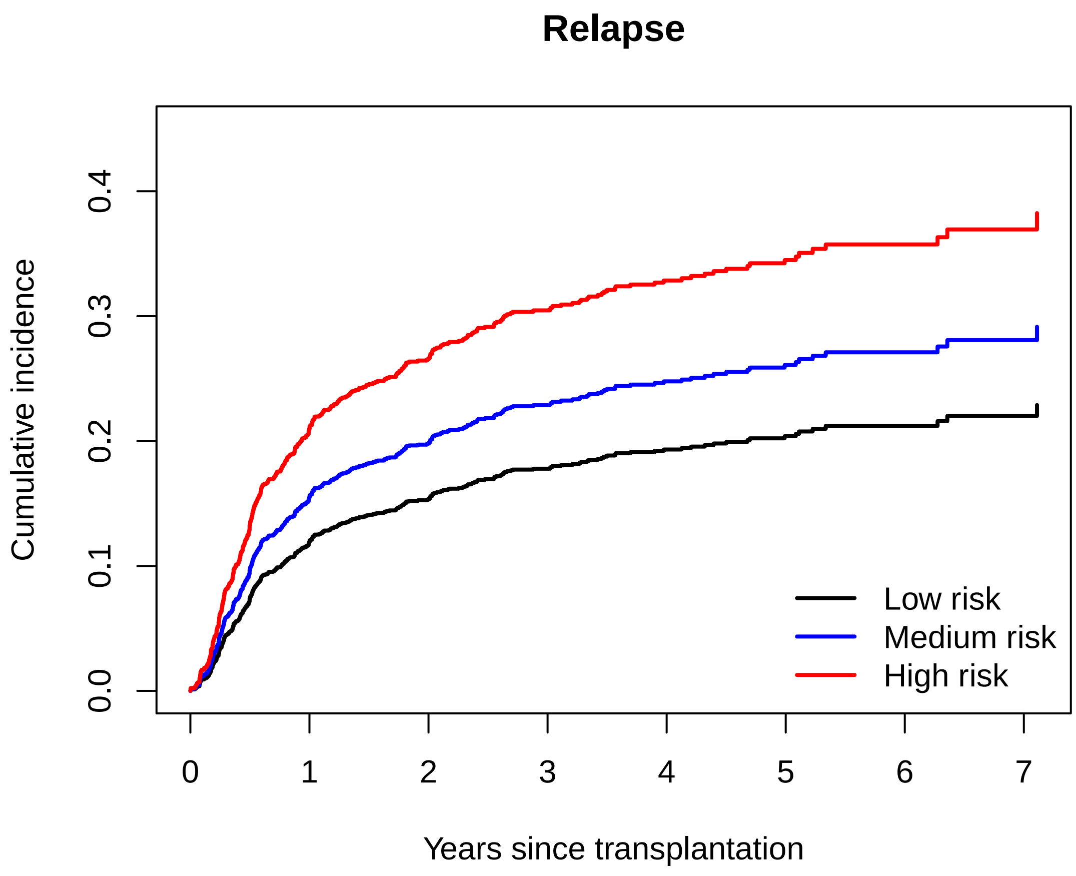

We gather the results in a single data frame.

3.1.5 Comparison

All the plots together:

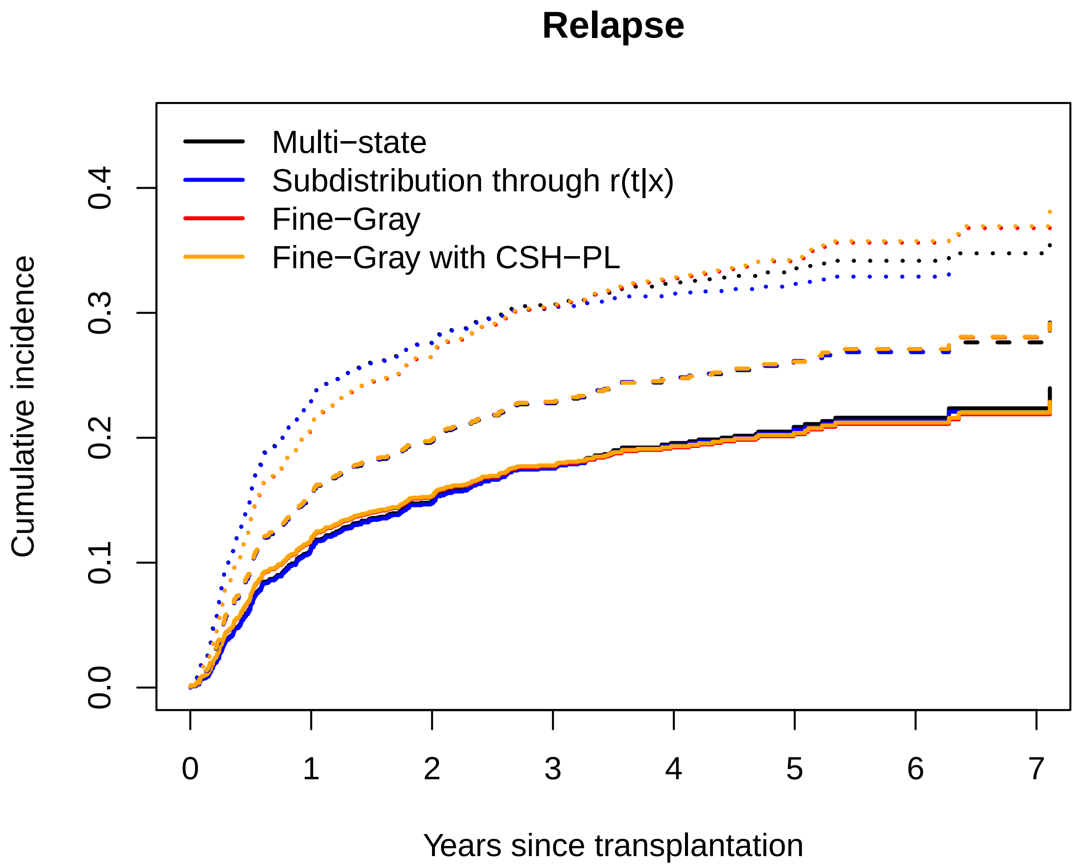

The black and white version in the paper:

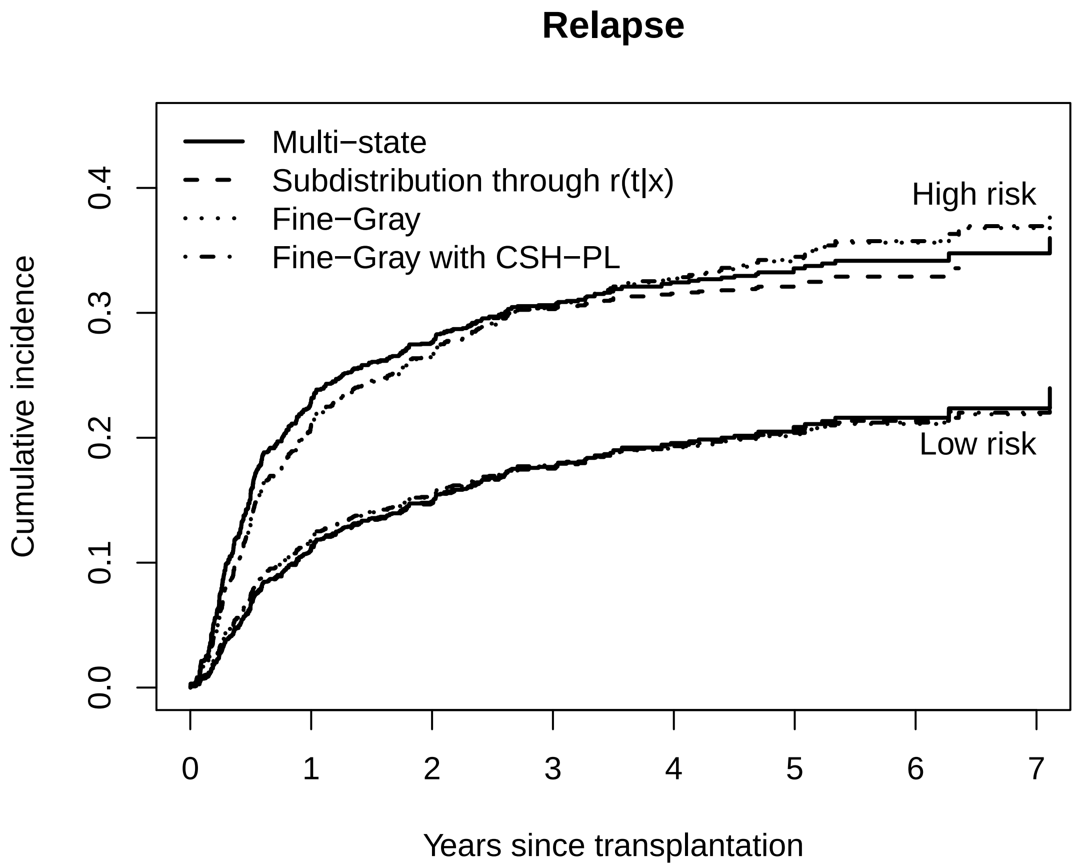

3.2 Non-relapse mortality

3.2.1 Multi-state model approach

Plot with the three risk groups (`age10`=0):

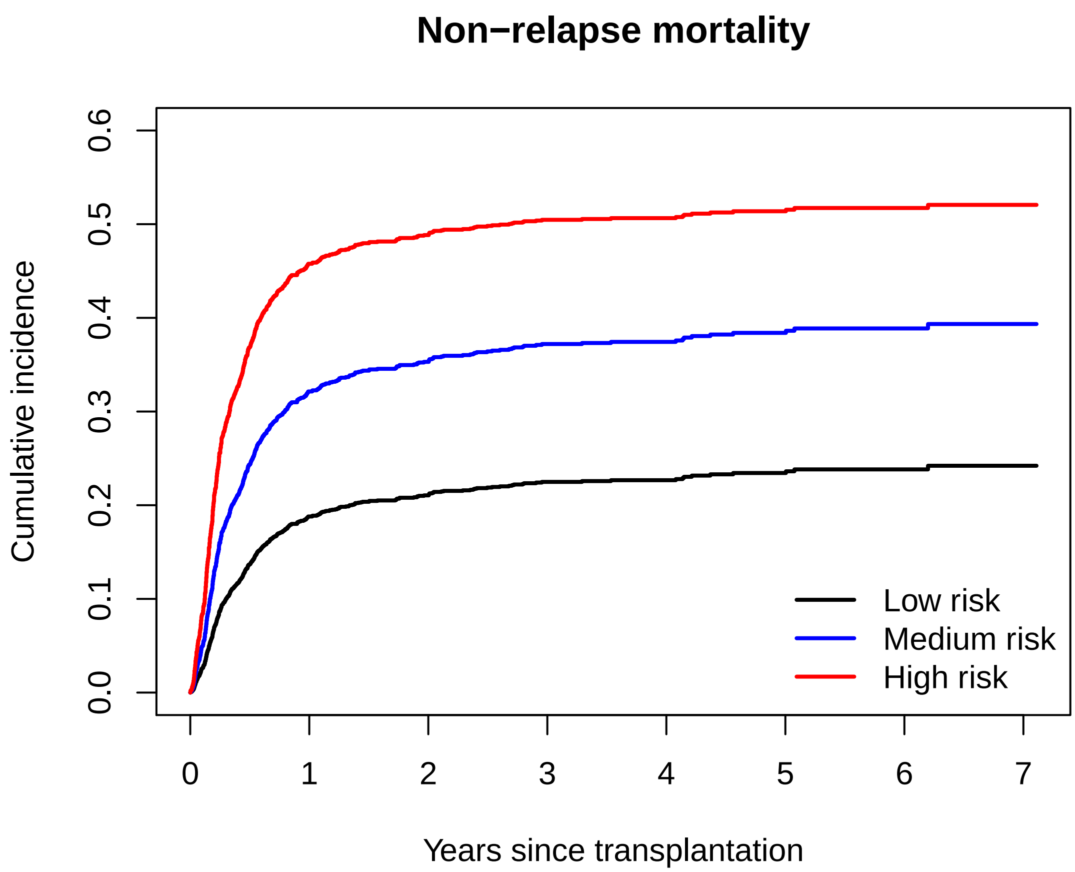

3.2.2 Subdistribution rate as product of cause-specific hazard and $r(t|x)$

The non-relapse mortality cumulative incidence curves for `age10`=0 and the three risk groups.

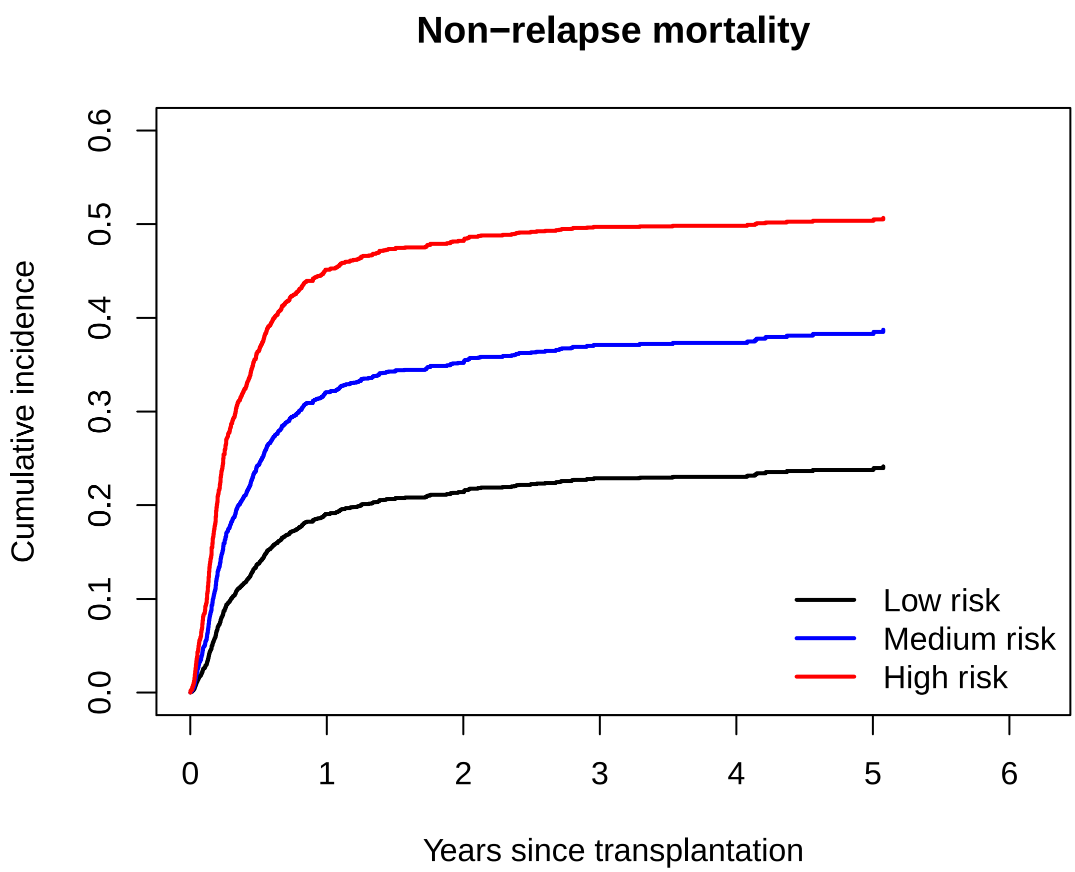

We gather the results in a single data frame.

3.2.3 Fine-Gray

Here, estimates for the cumulative incidences are based on the `predict` function in the `cmprsk` package, using the Fine-Gray results of Section 3.2. First recall the Fine-Gray model of Section 3.2.

```
## convergence:  TRUE
## coefficients:
## scoreMedium risk   scoreHigh risk           age10
##          0.58370          1.00400         0.04109
## standard errors:
## [1] 0.13530 0.17390 0.04195
## two-sided p-values:
## scoreMedium risk   scoreHigh risk           age10
##          1.6e-05          7.8e-09         3.3e-01
```

Now we concentrate on cause 2 (NRM), and show the NRM cumulative incidence curves for `age10`=0 and the three risk groups.

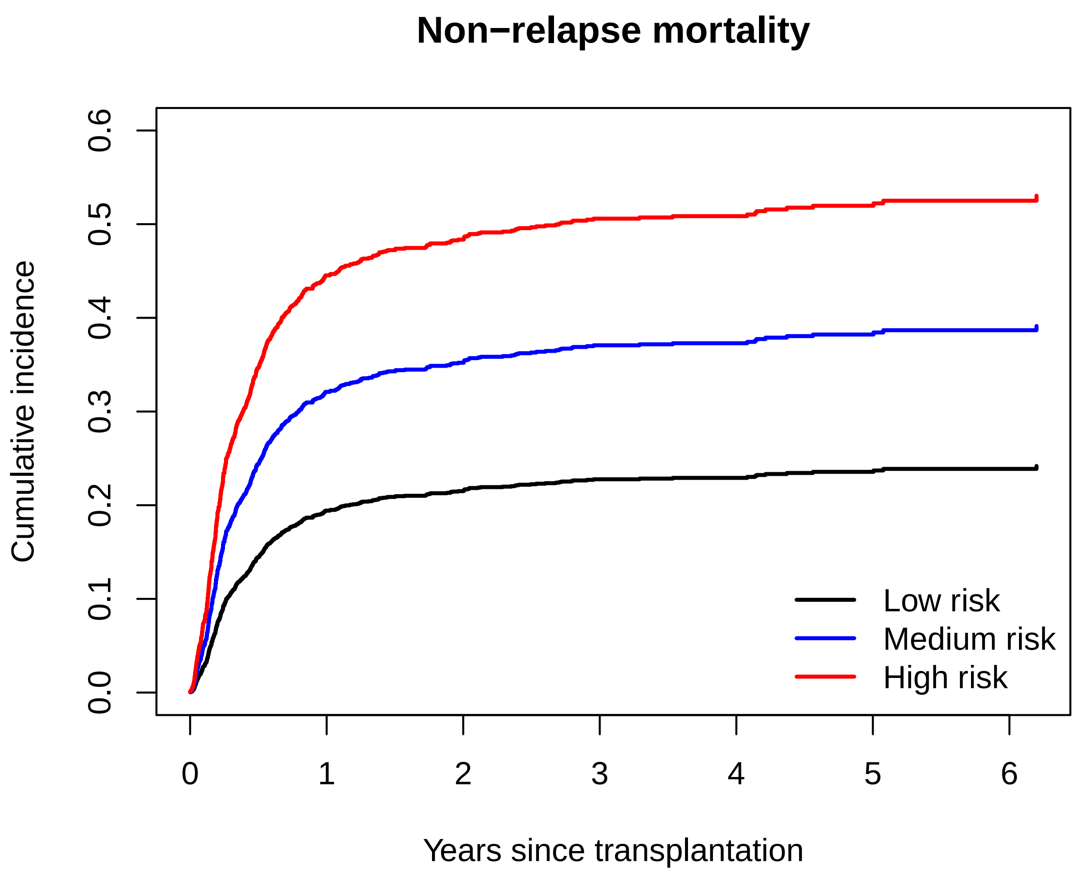

We gather the results in a single data frame.

3.2.4 Fine-Gray through $r(t|x)$

Here we fit a proportional subdistribution rate model, using the usual (cause-specific) log partial likelihood, with -log $\hat{r}(t|x)$ as offset. We no longer expect exactly the same result as in the previous subsection, but probably something quite close.

```
## $par
## [1] 0.58464381 1.00558004 0.03993351
##
## $value
## [1] 4550.234
##
## $counts
## function gradient
##       70       NA
##
## $convergence
## [1] 0
```

```
##
## $message
## NULL
```

```
## [1] 4550.234
```

Again the baseline hazard has been saved on the way, and the cumulative incidence curves can now be plotted.

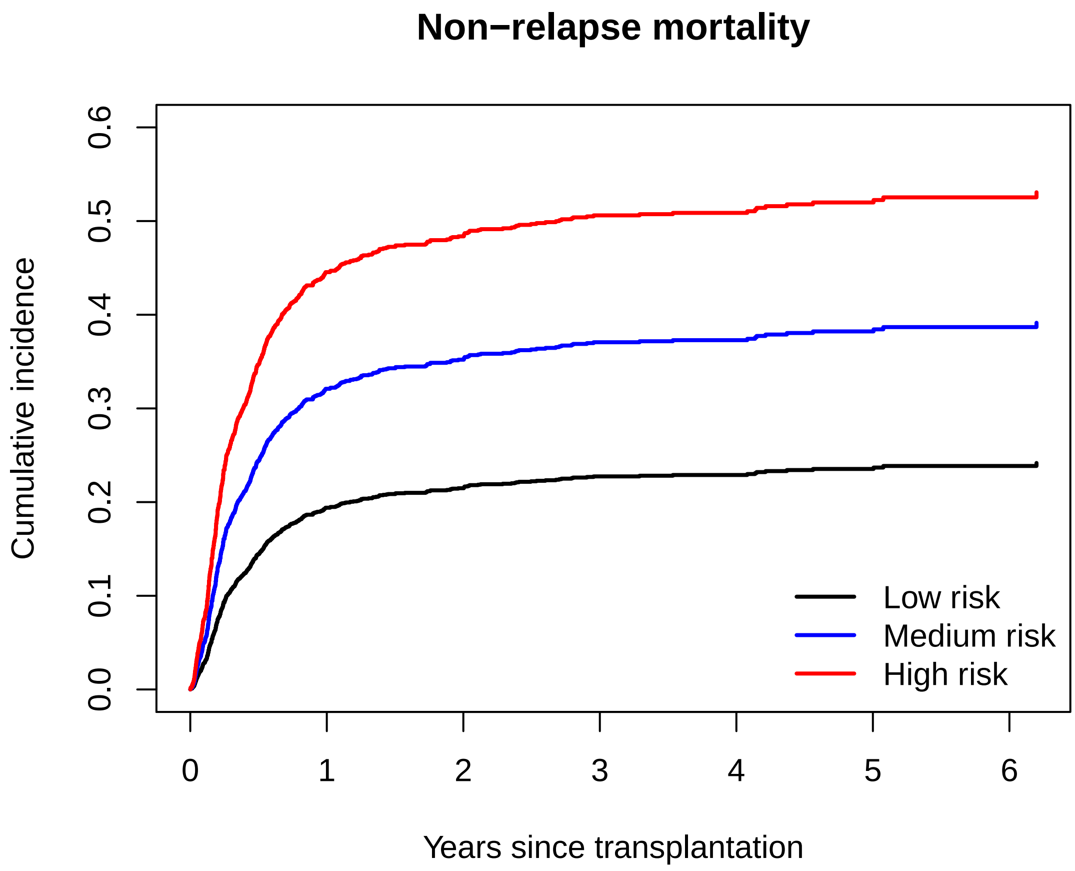

We gather the results in a single data frame.

3.2.5 Comparison

All the plots together:

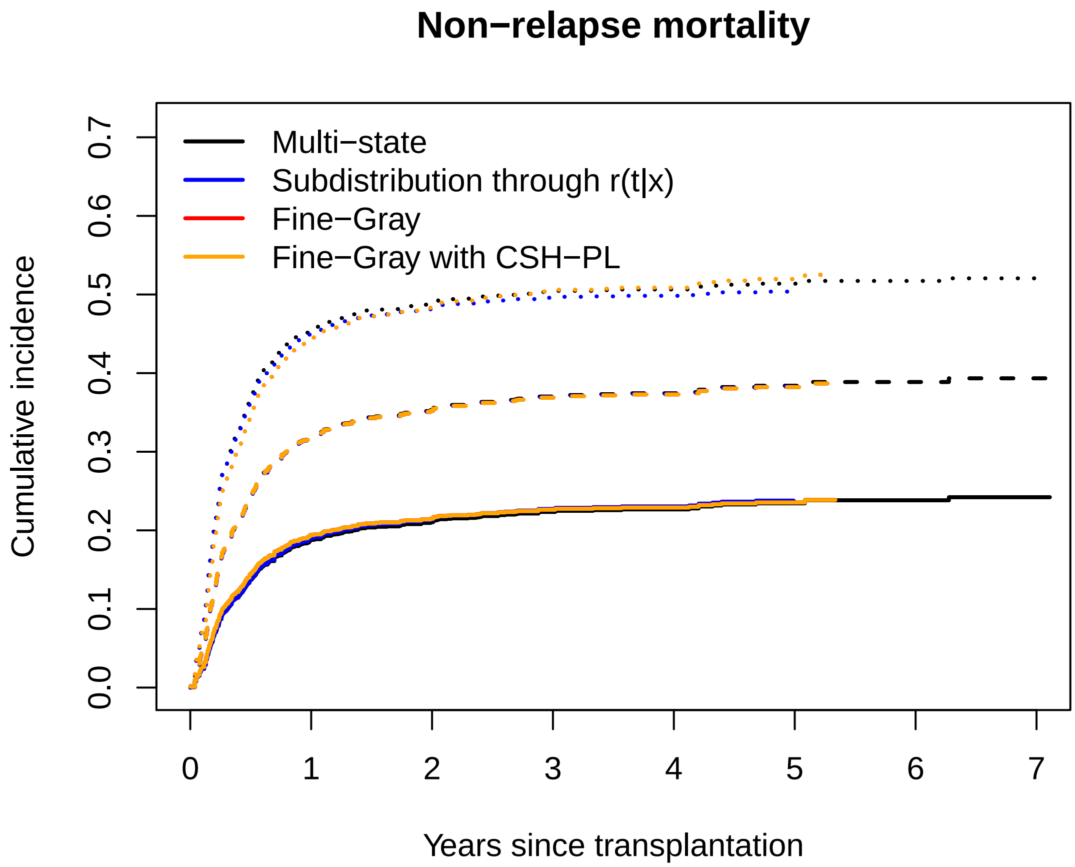

The black and white version in the paper:

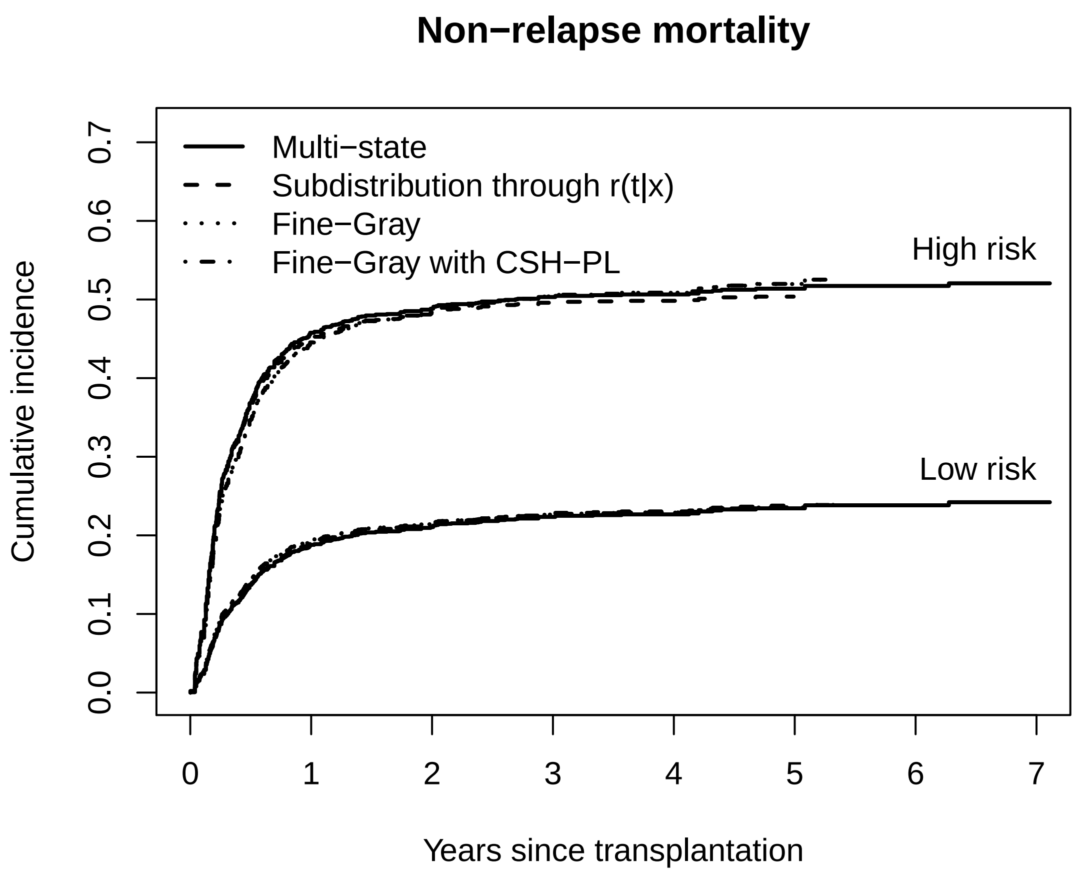

4 Asymptotic standard errors for the cumulative incidences

This is only done for relapse; code for NRM is similar.

4.1 Relapse

4.1.1 Multi-state approach

The standard errors have already been collected in Section 3.1.1.

4.1.2 Subdistribution rate as product of cause-specific hazard and $r(t|x)$

Everything needed for $r(t|x)$ is stored in `coefsRel`; the other thing needed is the vector of regression coefficients for the cause-specific hazards of relapse, which is stored in `c1a`.

It turns out that much of the code is very similar to what is already available in the function `lpl` (for relapse). We copy much of that code and use what we need. Some preliminary settings that are also useful for the standard errors of the other approaches are provided first.

The code in `lpl` uses `d` as data frame, hence we have defined `d` as `ebmt1`. The following output provides (as check) the standard errors of $\hat{\beta}_{\text{CS}}$, computed along the way. The plot shows the resulting standard errors of the cumulative incidences for low, medium and high risk (and `age10`=40).

```
## [1] 0.14916992 0.20467988 0.05318443
```

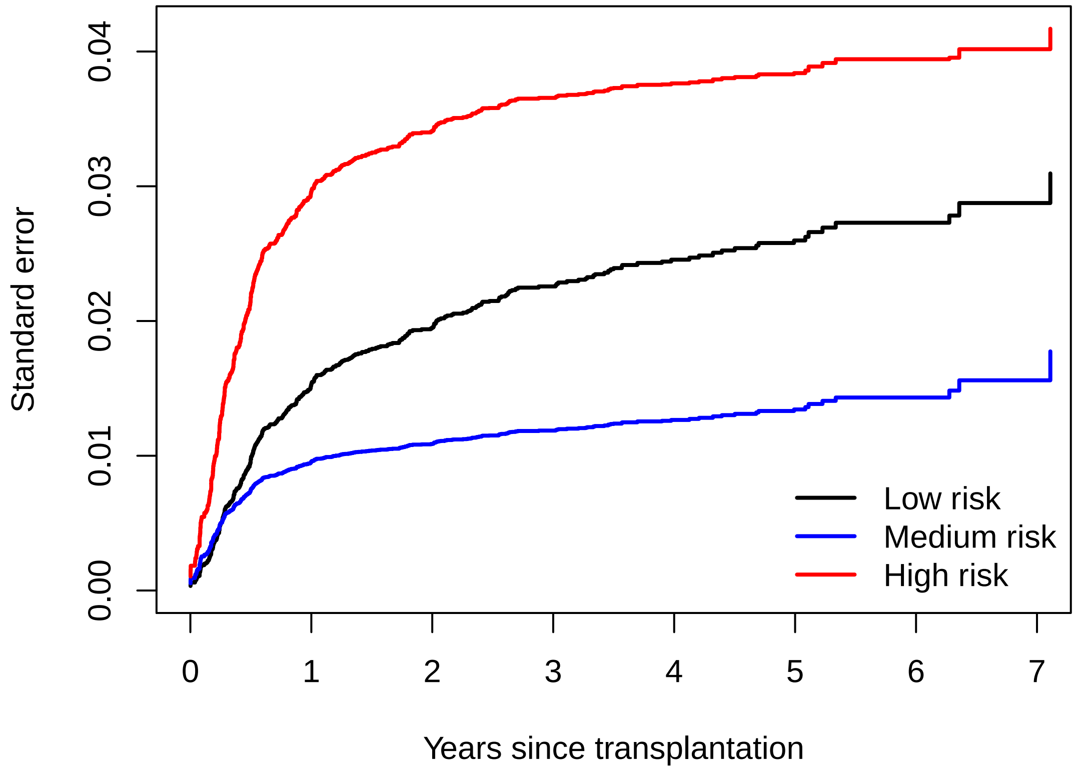

4.1.3 Fine-Gray

The code again is quite similar to that of the previous subsection. The weights are different (inverse probability of censoring weights), and the risk set is the Fine-Gray risk set. The following output provides (as check) the standard errors of $\hat{\beta}_{\text{FG}}$, computed along the way. The plot shows the resulting standard errors of the cumulative incidences for low, medium and high risk (and `age10`=40).

```
## [1] 0.14845708 0.20423822 0.05320406
```

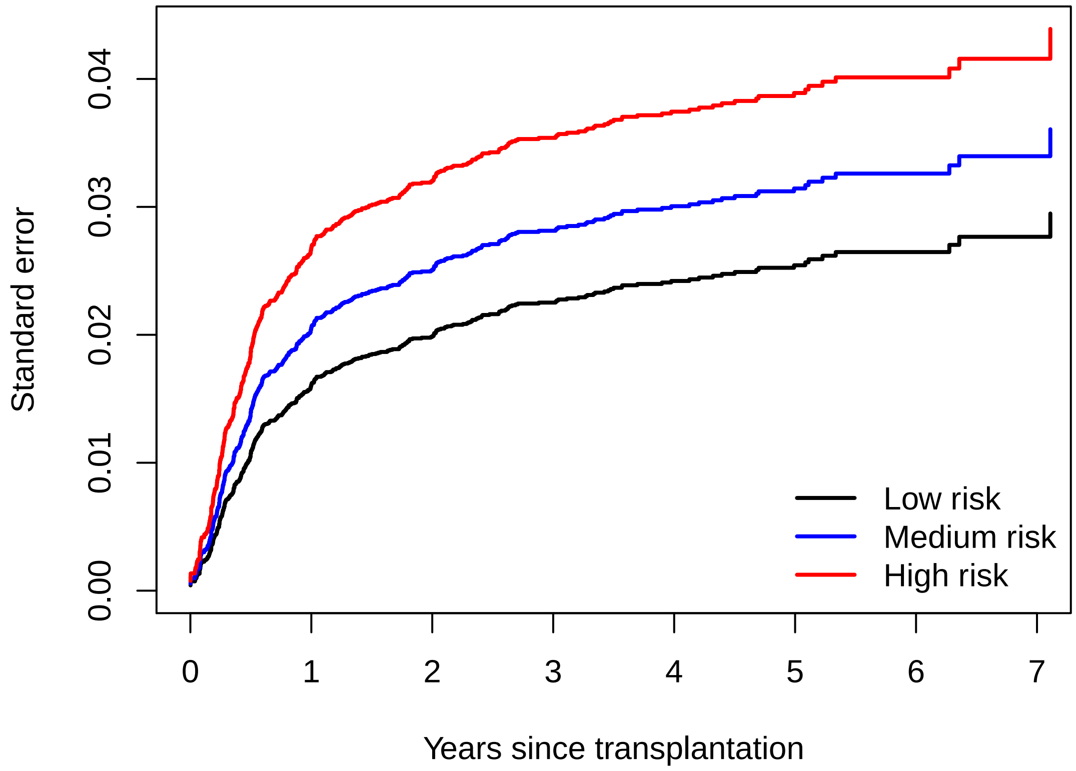

4.1.4 Fine-Gray through $r(t|x)$

The estimated regression coefficients $\tilde{\beta}_{RM}$ are stored in `optfgrtxRel`, $r(t|x)$ again in `coefsRel`.

The code again turns out to be quite similar to that of the previous subsection, and it uses the natural risk sets again. The following output provides (as check) the standard errors of $\tilde{\beta}_{\text{FG}}$, computed along the way. The plot shows the resulting standard errors of the cumulative incidences for low, medium and high risk (and `age10`=40).

```
## [1] 0.14895257 0.20316373 0.05316295
```

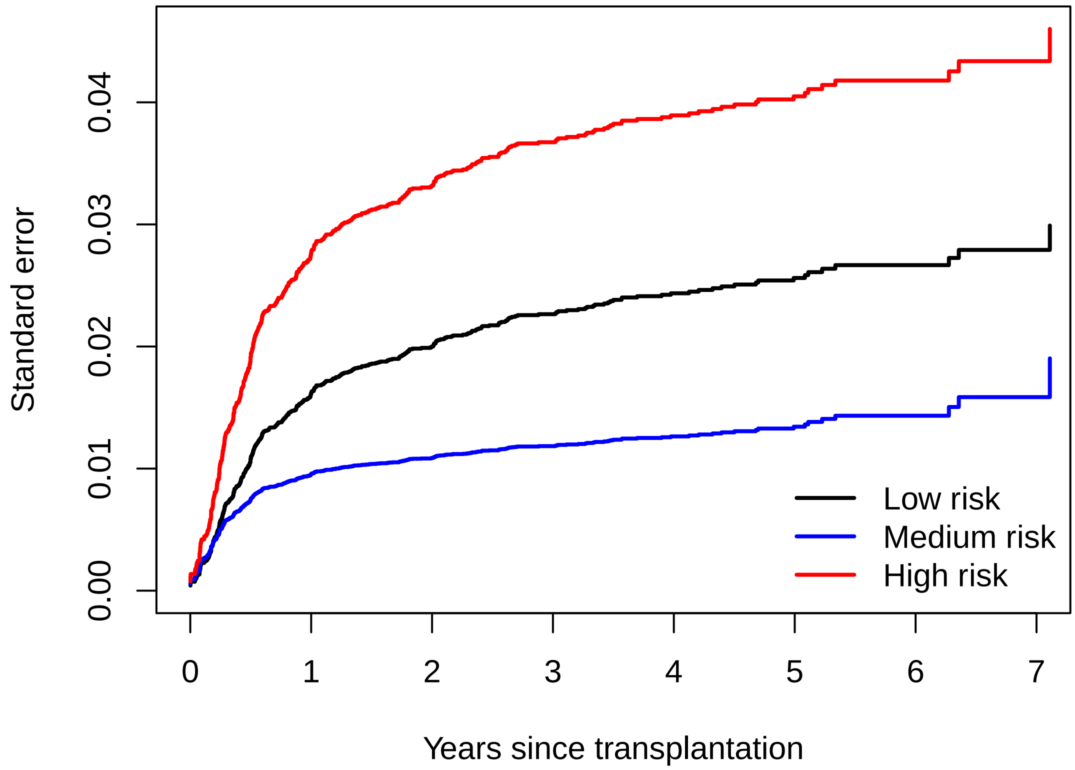

4.1.5 Comparison

Let's gather all the information and plot the results of the standard errors. Actually, to see whether we have gather the information correctly, we will start with plotting the cumulative incidence estimators of the four models.

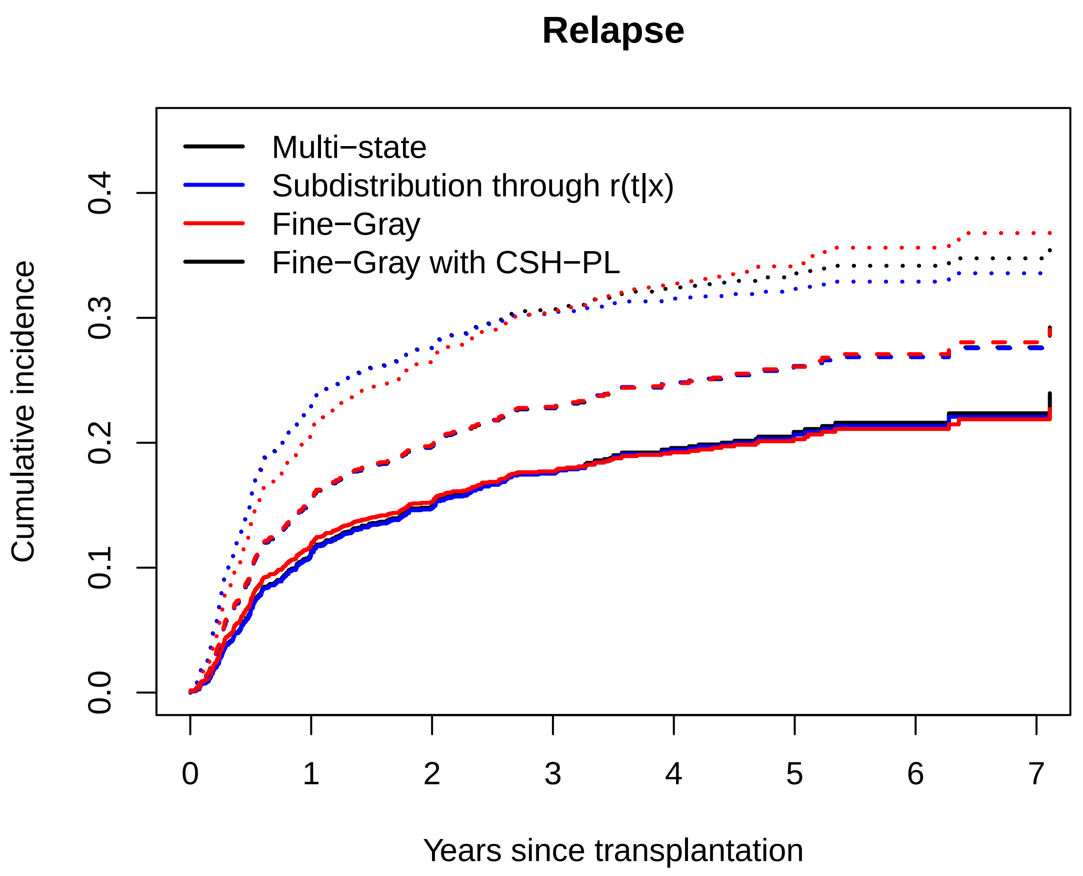

And here are the corresponding standard errors, for the high and low risk groups only.

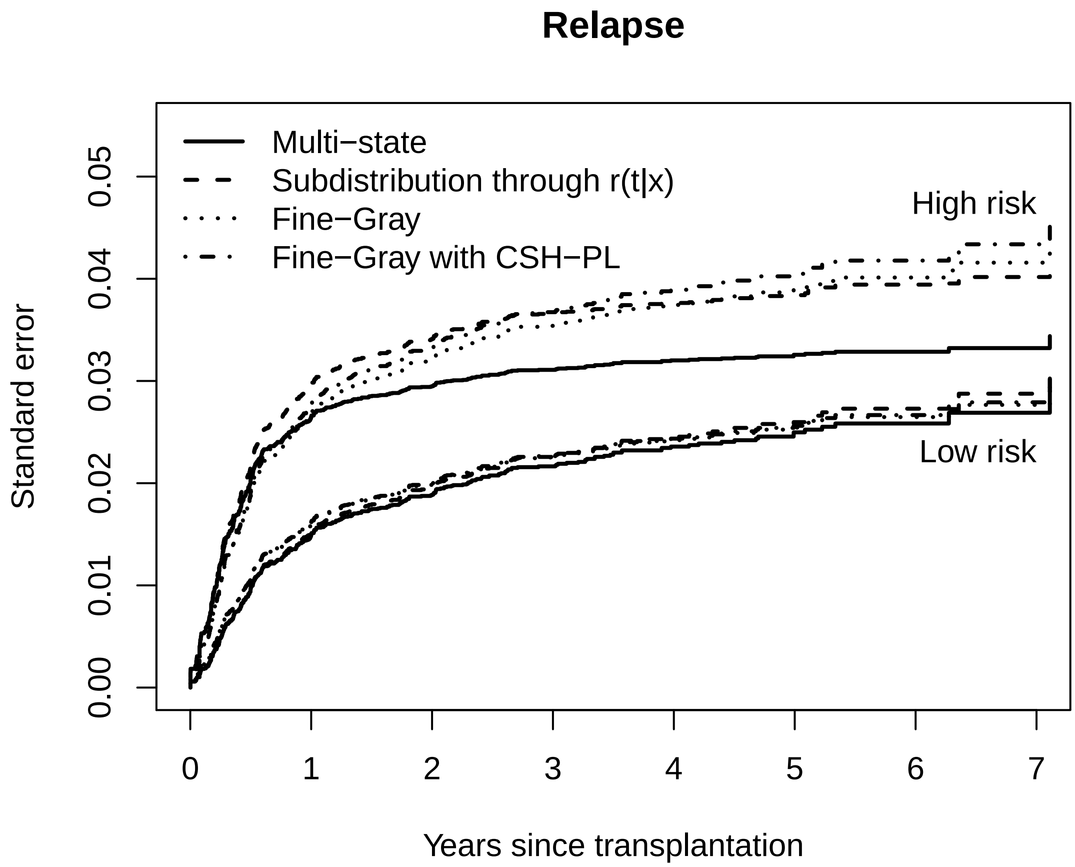

Relapse
Multi−state
Subdistribution through r(t|x)
Fine−Gray
Fine−Gray with CSH−PL
High risk
Low risk
Standard error
0.00
0.01
0.02
0.03
0.04
0.05
0
1
2
3
4
5
6
7
Years since transplantation
