# Supplementary material for: On the relation between the cause‐specific hazard and the subdistribution rate for competing risks data: The Fine–Gray model revisited
Source: Biom J. 2020 Mar 4;62(3):790–807. doi: 10.1002/bimj.201800274 (PMC7216972; doi:10.1002/bimj.201800274)
Supplement: Supplementary file 1 — Supporting Information [file BIMJ-62-790-s001.zip › Visualize_rtx_new.pdf]

Visualize r(t|x)

Hein Putter

11 April, 2019

Set-up

I have taken a model with two competing risks, where the (baseline) cause-specific hazard of both causes is given by $a_j(t) = abt^{b-1}$, for $a, b > 0$, corresponding to a Weibull distribution with parameters a and b. We choose three values of the shape parameter b, namely $b = 0.5$ (a decreasing hazard), $b = 1$ (constant hazard), and $b = 2$ (increasing hazard) and choose the corresponding rate parameters a in such a way that $S(2.5; a, b) = 0.05$, with $S(t; a, b) = \exp(-\int_0^t h(s; a, b)ds)$ the survival function corresponding to $h(t; a, b)$. This leads to the values 1.895, 1.198, and 0.479, for a, when $b = 0.5, 1, 2$, respectively. We consider a competing risks situation with two causes, where the cause-specific hazards of cause 1 and 2 are both chosen from these three Weibull hazards, with $b = 0.5, 1, 2$ and corresponding a values. We denote the hazard with $b = 0.5$ as "Early risk", with $b = 1$ as "Middle risk", and with $b = 2$ as "Late risk".

No covariates

The reduction factor $r(t)$ is obtained by numerically solving the differential equation, Equation (4) in the paper. This results in the following plot.

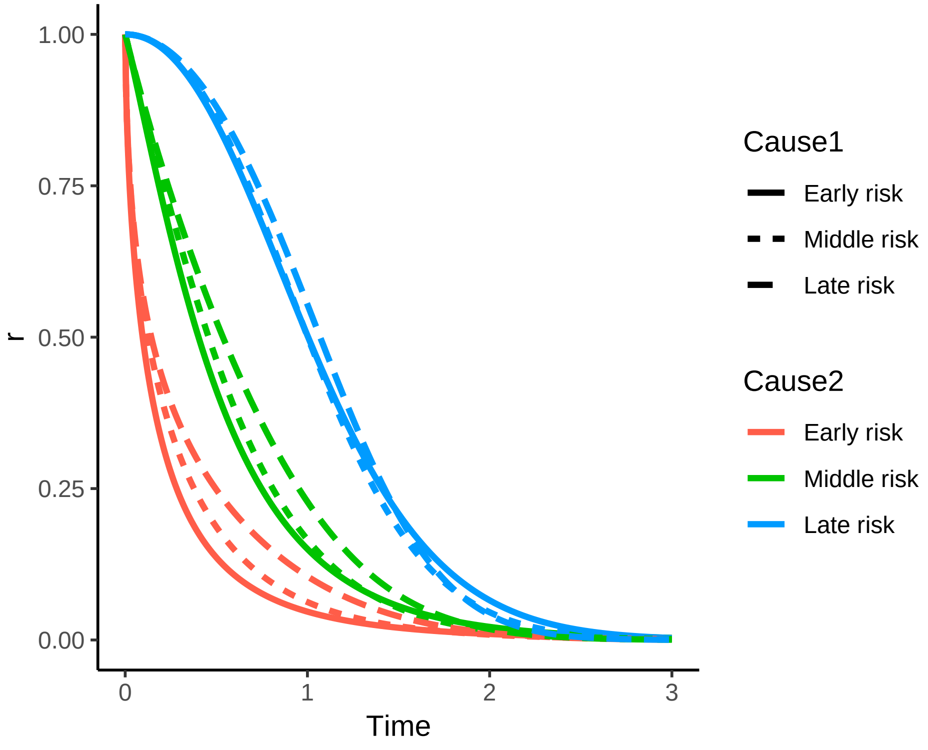

Covariates

A single covariate x is considered, and parameters β_1 and β_2 quantify the effect of x on the cause-specific hazards of cause 1 and 2, respectively. So the cause-specific hazard of cause j is given by

$$h_j(t \mid x) = a_j \exp(\beta_j x) b_j t^{b_j - 1}.$$

The formula for $r(t \mid x)$, when $b_1 = b_2 = b$, is given by

$$r(t \mid x) = \frac{\exp(-(a_1 e^{\beta_1 x} + a_2 e^{\beta_2 x}) t^b)}{\frac{a_2 e^{\beta_2 x}}{a_1 e^{\beta_1 x} + a_2 e^{\beta_2 x}} + \frac{a_1 e^{\beta_1 x}}{a_1 e^{\beta_1 x} + a_2 e^{\beta_2 x}} \exp(-(a_1 e^{\beta_1 x} + a_2 e^{\beta_2 x}) t^b)}.$$

When $b_1 \neq b_2$ numerical solutions need to be found.

We choose Weibull hazards as baseline hazards, for the cause-specific hazards of cause 1 and 2.

The plots are prepared using lattice.

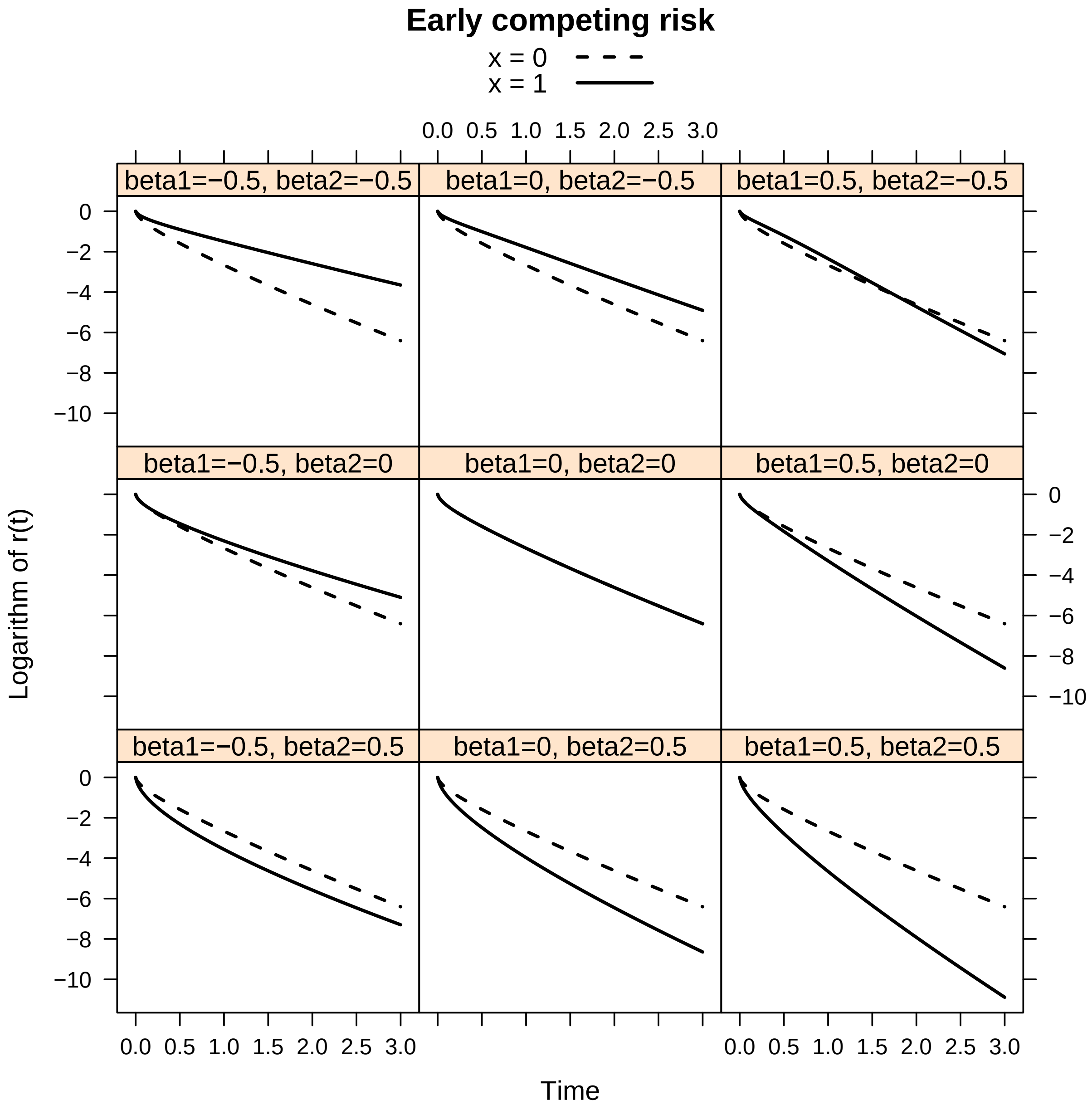
Early competing risk
x = 0
x = 1
beta1=−0.5, beta2=−0.5
beta1=0, beta2=−0.5
beta1=0.5, beta2=−0.5
beta1=−0.5, beta2=0
beta1=0, beta2=0
beta1=0.5, beta2=0
beta1=−0.5, beta2=0.5
beta1=0, beta2=0.5
beta1=0.5, beta2=0.5
Logarithm of r(t)
Time
0.0 0.5 1.0 1.5 2.0 2.5 3.0
0 −2 −4 −6 −8 −10

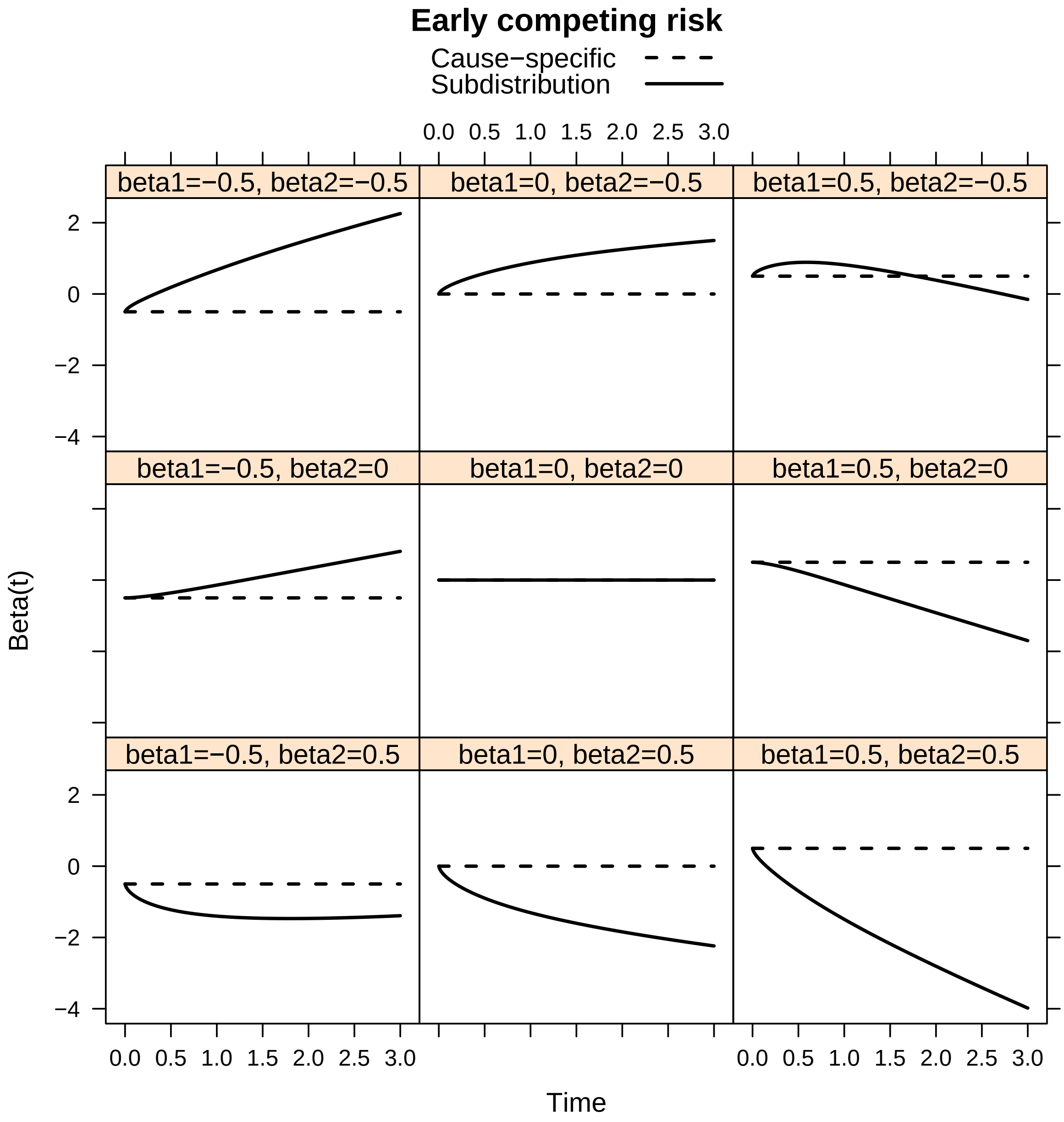

Early competing risk
Cause−specific
Subdistribution
beta1=−0.5, beta2=−0.5
beta1=0, beta2=−0.5
beta1=0.5, beta2=−0.5
beta1=−0.5, beta2=0
beta1=0, beta2=0
beta1=0.5, beta2=0
beta1=−0.5, beta2=0.5
beta1=0, beta2=0.5
beta1=0.5, beta2=0.5
Beta(t)
Time
0.0 0.5 1.0 1.5 2.0 2.5 3.0
2 0 −2 −4

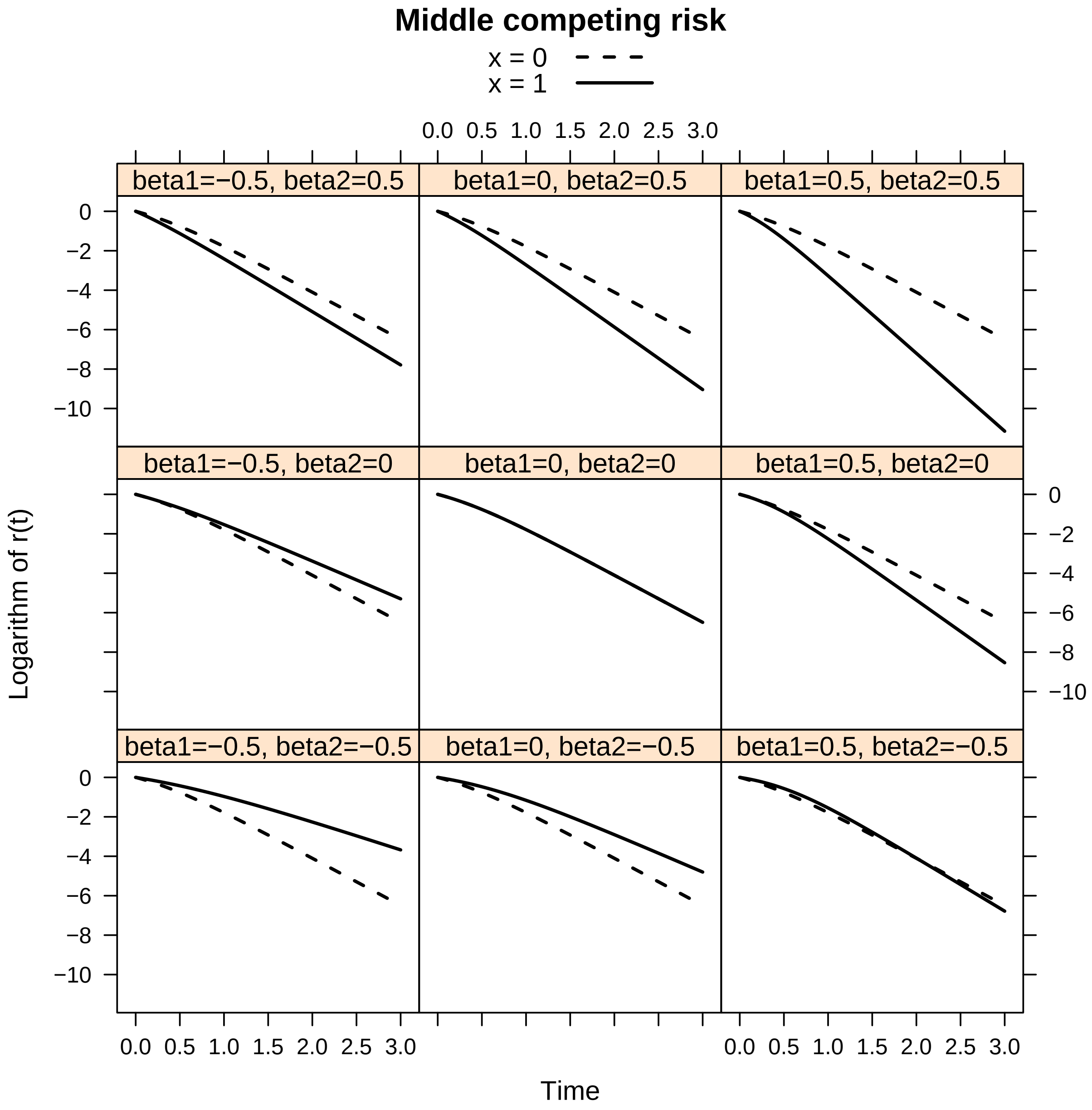

Middle competing risk
x = 0
x = 1
beta1=−0.5, beta2=0.5
beta1=0, beta2=0.5
beta1=0.5, beta2=0.5
beta1=−0.5, beta2=0
beta1=0, beta2=0
beta1=0.5, beta2=0
beta1=−0.5, beta2=−0.5
beta1=0, beta2=−0.5
beta1=0.5, beta2=−0.5
Logarithm of r(t)
Time
0.0 0.5 1.0 1.5 2.0 2.5 3.0
0 −2 −4 −6 −8 −10

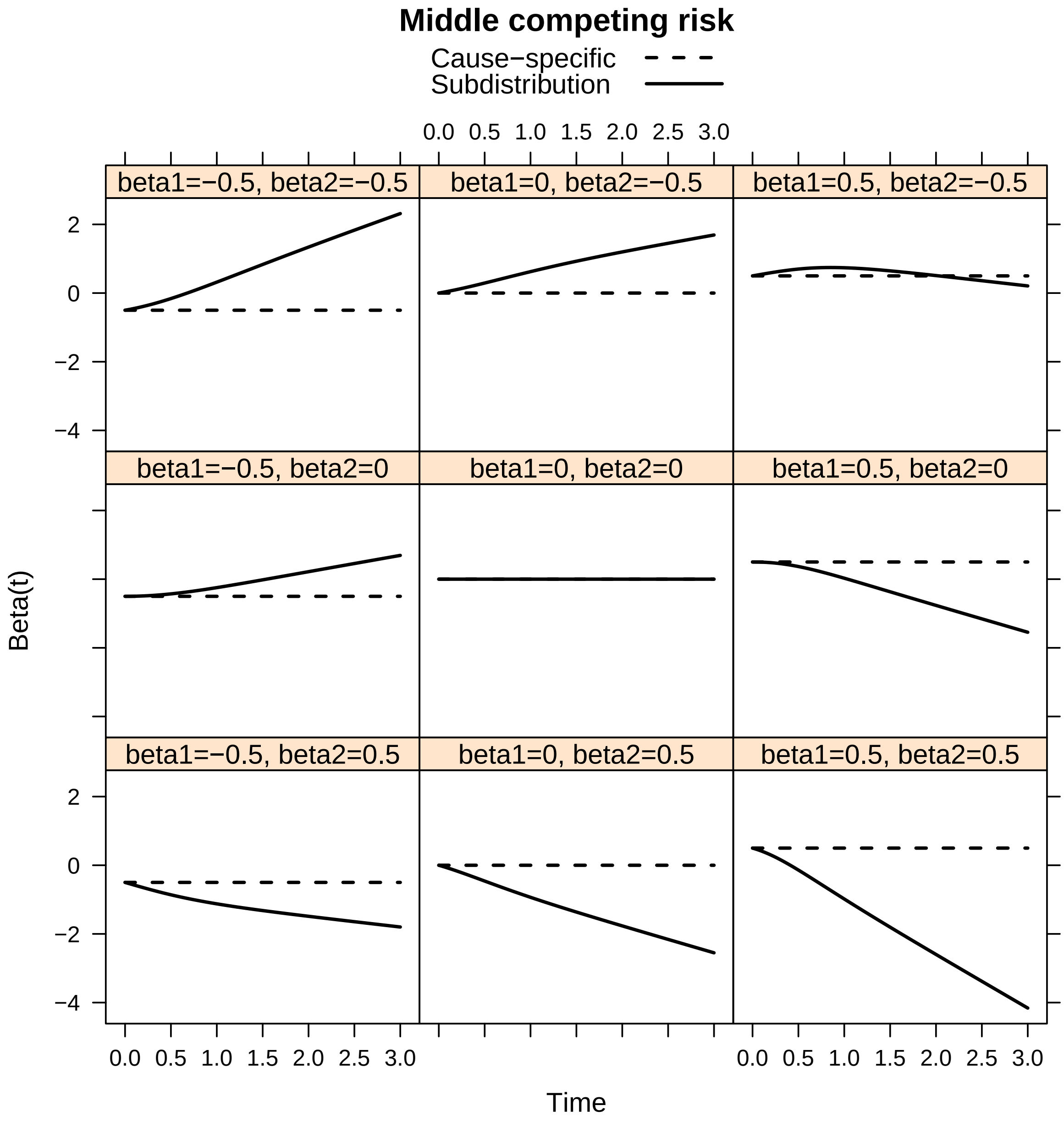
Middle competing risk
Cause−specific
Subdistribution
beta1=−0.5, beta2=−0.5
beta1=0, beta2=−0.5
beta1=0.5, beta2=−0.5
beta1=−0.5, beta2=0
beta1=0, beta2=0
beta1=0.5, beta2=0
beta1=−0.5, beta2=0.5
beta1=0, beta2=0.5
beta1=0.5, beta2=0.5
Beta(t)
Time
0.0 0.5 1.0 1.5 2.0 2.5 3.0
2 0 −2 −4

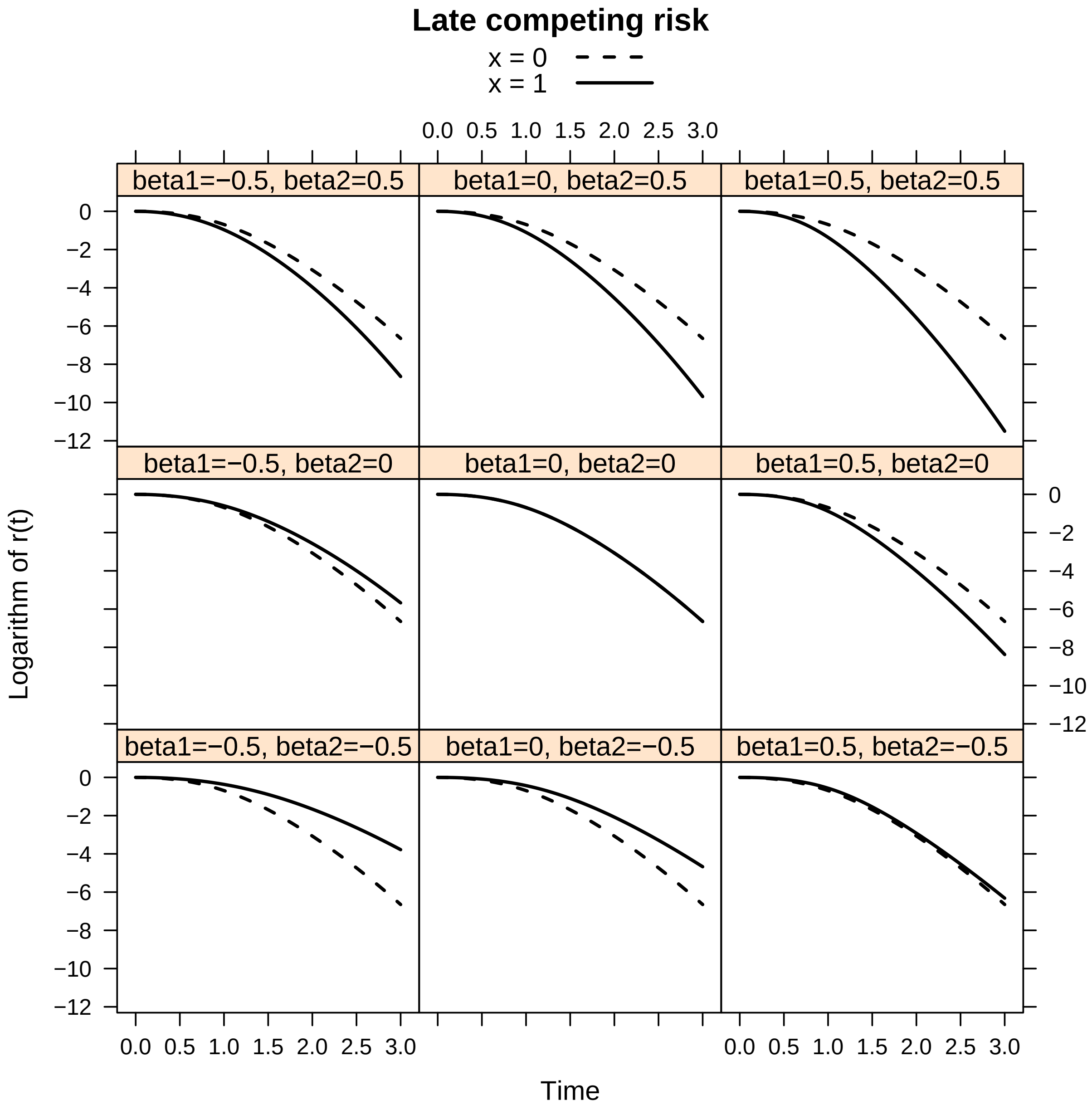

Late competing risk
x = 0
x = 1
beta1=−0.5, beta2=0.5
beta1=0, beta2=0.5
beta1=0.5, beta2=0.5
beta1=−0.5, beta2=0
beta1=0, beta2=0
beta1=0.5, beta2=0
beta1=−0.5, beta2=−0.5
beta1=0, beta2=−0.5
beta1=0.5, beta2=−0.5
Logarithm of r(t)
Time
0.0 0.5 1.0 1.5 2.0 2.5 3.0
0 −2 −4 −6 −8 −10 −12

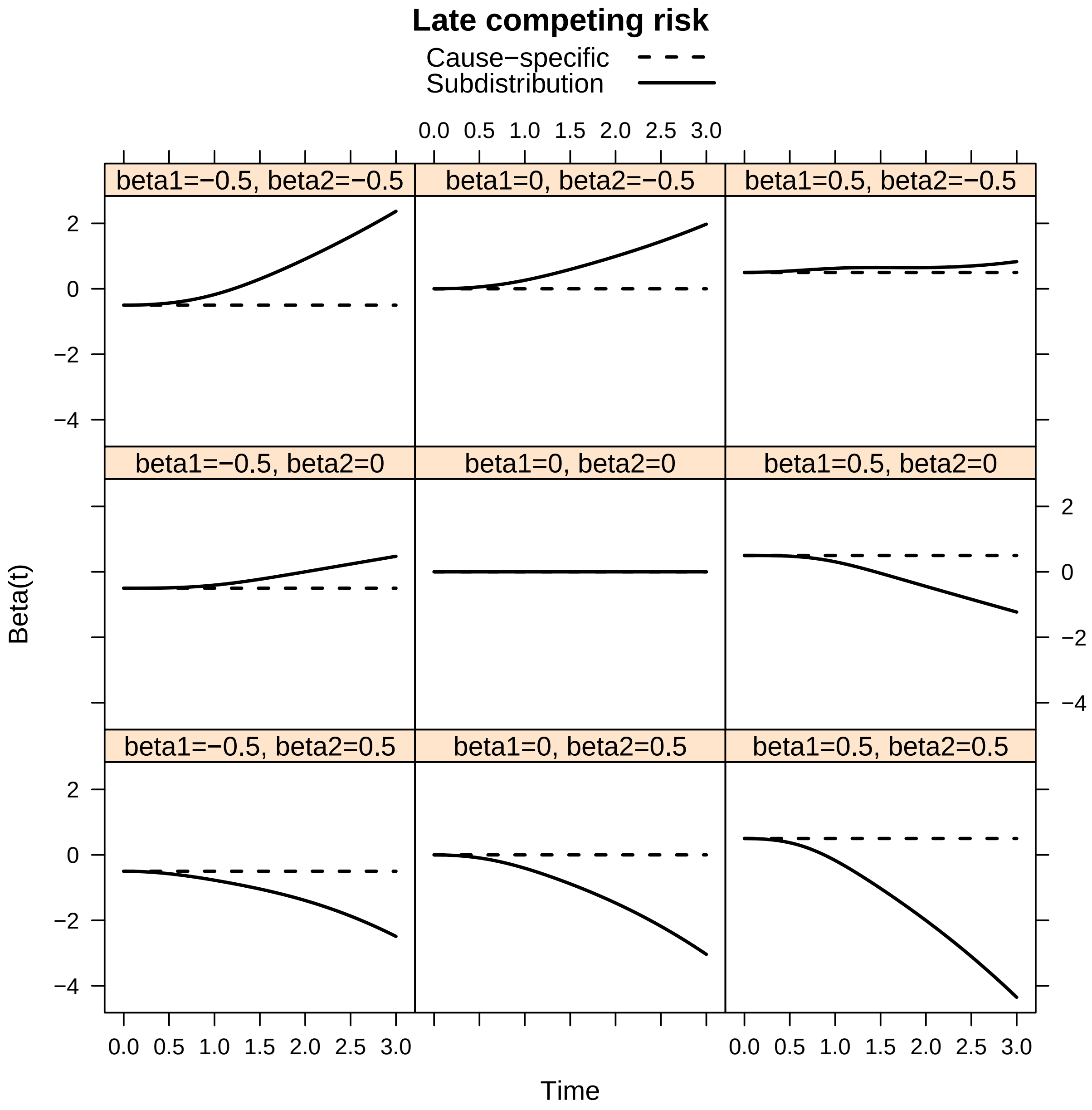

Late competing risk
Cause−specific
Subdistribution
beta1=−0.5, beta2=−0.5
beta1=0, beta2=−0.5
beta1=0.5, beta2=−0.5
beta1=−0.5, beta2=0
beta1=0, beta2=0
beta1=0.5, beta2=0
beta1=−0.5, beta2=0.5
beta1=0, beta2=0.5
beta1=0.5, beta2=0.5
Beta(t)
Time
0.0 0.5 1.0 1.5 2.0 2.5 3.0
2 0 −2 −4
